# Natural Variation of the *RICE FLOWERING LOCUS T 1* Contributes to Flowering Time Divergence in Rice

**DOI:** 10.1371/journal.pone.0075959

**Published:** 2013-10-01

**Authors:** Eri Ogiso-Tanaka, Kazuki Matsubara, Shin-ichi Yamamoto, Yasunori Nonoue, Jianzhong Wu, Hiroko Fujisawa, Harumi Ishikubo, Tsuyoshi Tanaka, Tsuyu Ando, Takashi Matsumoto, Masahiro Yano

**Affiliations:** Agrogenomics Research Center, National Institute of Agrobiological Sciences, Kannondai Tsukuba, Ibaraki, Japan; Nanjing Agricultural University, China

## Abstract

In rice (*Oryza sativa* L.), there is a diversity in flowering time that is strictly genetically regulated. Some *indica* cultivars show extremely late flowering under long-day conditions, but little is known about the gene(s) involved. Here, we demonstrate that functional defects in the florigen gene *RFT1* are the main cause of late flowering in an *indica* cultivar, Nona Bokra. Mapping and complementation studies revealed that sequence polymorphisms in the *RFT1* regulatory and coding regions are likely to cause late flowering under long-day conditions. We detected polymorphisms in the promoter region that lead to reduced expression levels of *RFT1*. We also identified an amino acid substitution (E105K) that leads to a functional defect in Nona Bokra RFT1. Sequencing of the *RFT1* region in rice accessions from a global collection showed that the E105K mutation is found only in *indica*, and indicated a strong association between the *RFT1* haplotype and extremely late flowering in a functional *Hd1* background. Furthermore, SNPs in the regulatory region of *RFT1* and the E105K substitution in 1,397 accessions show strong linkage disequilibrium with a flowering time–associated SNP. Although the defective E105K allele of *RFT1* (but not of another florigen gene, *Hd3a*) is found in many cultivars, relative rate tests revealed no evidence for differential rate of evolution of these genes. The ratios of nonsynonymous to synonymous substitutions suggest that the E105K mutation resulting in the defect in *RFT1* occurred relatively recently. These findings indicate that natural mutations in *RFT1* provide flowering time divergence under long-day conditions.

## Introduction

The appropriate flowering time is important for reproductive success in plants. Flowering time is controlled by developmental regulation and environmental conditions, such as daylength (photoperiod) and temperature [1,2]. Rice (*Oryza sativa* L.), a facultative short-day (SD) plant, flowers when the days become shorter than a critical daylength [3]. Rice is grown in tropical, subtropical and temperate regions, and variation in flowering time (called “heading date” in rice) allows it to adapt to different climate conditions. The high level of flowering time variation is also one of the most important factors in rice breeding. A number of flowering time QTLs and genes have been identified and characterized by QTL analysis using natural variation in rice. *Hd1/Se1, Ehd1, Ghd7/Lhd4, DTH8/Ghd8/Lhd1/Hd5, Hd6, Hd16, Hd17* and *Hd3a* have been identified as rice flowering time genes [4-20]. *Hd1*, which has several alleles, promote flowering under SD and represses it under long-day (LD) conditions [4]. The loss of *Hd1* function, which results in a decrease in photoperiodic response, has enabled expansion of the cultivation area of rice [21-26]. *Hd1* is the main source of flowering time diversity in cultivated rice [23]. *Ehd1* encodes a B-type response regulator. *Ehd1* is a key promoter of flowering under both SD and LD conditions [6]. Cultivars that have a defective *Ehd1* allele (*ef1*) have only been identified in Taiwan so far [6,27,28]. The *ef1* allele is useful for long vegetative growth period in low and mid-low latitude areas [29]. *Ghd7* encodes a CCT domain protein, which is a strong repressor of *Ehd1* expression [7,30]. *Ghd7* acts as a repressor of flowering under LD conditions, and the loss-of-function mutations in this gene cause early flowering under LD conditions [7,8]. Such defective *Ghd7* alleles are found in high-latitude areas in China and Japan [7,31]. *DTH8* encodes a putative HAP3 subunit of the CCAAT box–binding transcription factor, and is an ortholog of *AtHAP3b* in *Arabidopsis* [9-12]. *DTH8* promotes flowering under SD and represses it under LD conditions [10]. Many frame-shift mutations in *DTH8* cause a weak photoperiod response and early flowering in Asian cultivated rice [9-12]. Cultivars with double defects in *Lhd4* and *Hd5*, grown at the northern limit of the rice cultivation area in Japan, show extremely early flowering [32]. *Hd6* encodes the alpha subunit of casein kinase II, and represses flowering indirectly via Hd1 under LD conditions [13,14]. *Hd6* causes a strong photoperiod response and late flowering [15,16]. Recently, variation in *Hd16* and *Hd17* was found among *japonica* cultivars [17-19]. *Hd16* controls flowering by regulating Ghd7 activity [18]. Since *Hd16* loss-of-function plants show moderately early flowering, the non-functional allele has been used to breed cultivars able to grow in a wide range of areas in Japan [18]. *Hd17* encodes the ELF3-like protein [19], and is allelic to *Ef7* and *Hd3b* [33,34]. A single-nucleotide polymorphism (SNP) in *Hd17*, found as a natural mutation in *japonica* cv. Koshihikari, contributes to flowering variation via regulation of *Ghd7* [19]. The flowering time genes mentioned above act in one or more pathway(s) that regulate flowering time by controlling the florigen genes *Hd3a* and *RICE FLOWERING LOCUS T 1* (*RFT1*) [21,22,35,36].

Florigen was originally described as a product of *FLOWERING LOCUS T* (*FT*) in *Arabidopsis* [37]. FT and its orthologs in other plants are long-distance mobile floral stimuli that move from leaves to the shoot apex [37-40]. They are members of the phosphatidylethanolamine-binding protein (PEBP) family. Duplications and divergence of PEBP genes have produced three subfamilies in angiosperms: *FT, TFL1* and *MFT1* [41]. Variation of *FT-*like genes contributes to flowering time variation in *Arabidopsis* and several crops [42-47]. In rice, 19 PEBP genes have been described, and 13 of them are *FT-*like genes. *Hd3a, RFT1* and *FTL* have the ability to promote flowering [35], and *Hd3a* and *RFT1* are considered to be rice florigen genes because double *RFT1-Hd3a* RNAi plants do not flower [36]. The expression of *Hd3a* and *RFT1* is detected in leaf blades, and respective GFP fusion proteins have been detected in the shoot apical meristem and vascular tissue [39,40]. *Hd3a* RNAi plants delay flowering under SD, but not under LD conditions. In contrast, *RFT1* RNAi plants delay flowering under LD, but not under SD conditions. Thus, Hd3a and RFT1 function as florigens under SD and LD conditions, respectively [48]. Although *Hd3a* and *RFT1* are tandemly duplicated, highly homologous *FT*-like genes, they are regulated differently. Both are regulated by *Ehd1*, whereas *Hd3a* is also regulated by *Hd1* [40,48]. *RFT1* is also regulated by the SDG724 histone methyltransferase [49]. Thus, *Hd3a* and *RFT1* gene functions and regulation are relatively well understood. However, there is little experimental evidence regarding the contributions of natural mutations in *FT*-like genes in rice flowering time variation [20,23].

A region close to *Hd3a* and *RFT1* has been detected as a flowering time QTL among various cultivars and wild rice accessions [20,50-53]. Kojima et al. [20] provided evidence for natural mutations in *Hd3a* in cultivars Nipponbare (Nip) (*O. sativa* ssp. *japonica* ) and Kasalath (Kasa) (*O. sativa* ssp. *indica* ) using near-isogenic lines (NILs) and transgenic plants carrying the *NipHd3a* genomic region. Although both *KasaHd3a* and *NipHd3a* are functional and accelerate flowering, the effect of *KasaHd3a* is stronger, and its expression levels are slightly higher than those of *NipHd3a*, probably because of the differences in the regions encoding the C-termini (P179N) or polymorphism(s) in the 3′ untranslated regions (UTRs) [20]. Kojima et al. [20] also noted that *RFT1* promoted flowering. Dung et al. [53] reported the flowering time QTL *se-pat* in the *RFT1/Hd3a* region from *indica* cv. Patpaku. Sano et al. [54] reported the flowering time QTL enhancer of *Se1* (*En-Se1*) in this region from *Oryza* *rufipogon* (W593) and showed that *En-se1* strongly represses flowering in the presence of *Se1* (*Hd1*). Hagiwara et al. [50] resolved the *se-pat* and *en-se1* regions into three QTLs (*Hd3b, RFT1* and *Hd3a*), and showed that the nucleotide diversity in *RFT1* is higher than in *Hd3a*. Hagiwara et al. [50] and Ebana et al. [51] predicted two causal polymorphisms in the *Hd3a* and *RFT1* translated regions by comparing their sequences. Uga et al. [52] detected a flowering time QTL around the *Hd3a*/*RFT1* genomic regions by using an F_2_ population and backcross progenies from a cross between extremely late-flowering cv. Nona Bokra (ssp. *indica*) and early-flowering cv. Koshihikari (ssp. *japonica*), grown under natural-daylength field (ND) conditions. Although the *Hd3a* and *RFT1* genomic regions have been frequently detected by QTL analysis, no loss-of-function alleles have been reported in rice florigen genes. Takahashi et al. [23] found six types of *Hd3a* alleles in 64 cultivars from a rice core collection, some of them carrying three nonsynonymous substitutions. They also showed that the *Hd3a* promoter activity is similar in all alleles, and there is no clear relationship between *Hd3a* nucleotide sequence variation and flowering time variation. An SNP close to *Hd3a* and *RFT1*, strongly associated with the flowering time variation, was reported by a genome-wide association study that used a global collection of 950 rice varieties [55]. This suggests that the *Hd3a* and *RFT1* genomic region is involved in rice flowering time variation.

The purpose of this study was to identify the causal gene of extremely late flowering. We performed fine mapping by using progenies derived from a cross between Koshihikari and SL520 [56], a line containing an introgressed segment from Nona Bokra on chromosome 6 (Chr. 6). By using transformation and expression analysis, we also achieved molecular identification of a QTL for extremely late flowering that mapped to the *RFT1* and *Hd3a* region. Here we show that Nona Bokra *RFT1* is a defective gene and the main causal gene for extremely late flowering and no flowering under ND and LD conditions, respectively. Furthermore, by comparing the flowering times of different *RFT1* haplotypes, we show that the defective *RFT1* plays an important role in ‘extremely’ late flowering in rice. Therefore, we conclude that *RFT1* can have a considerable effect on the diversity of flowering time.

## Results

### Delimitation of a candidate genomic region for a late-flowering time QTL on the short arm of chromosome 6

**Figure 1:**
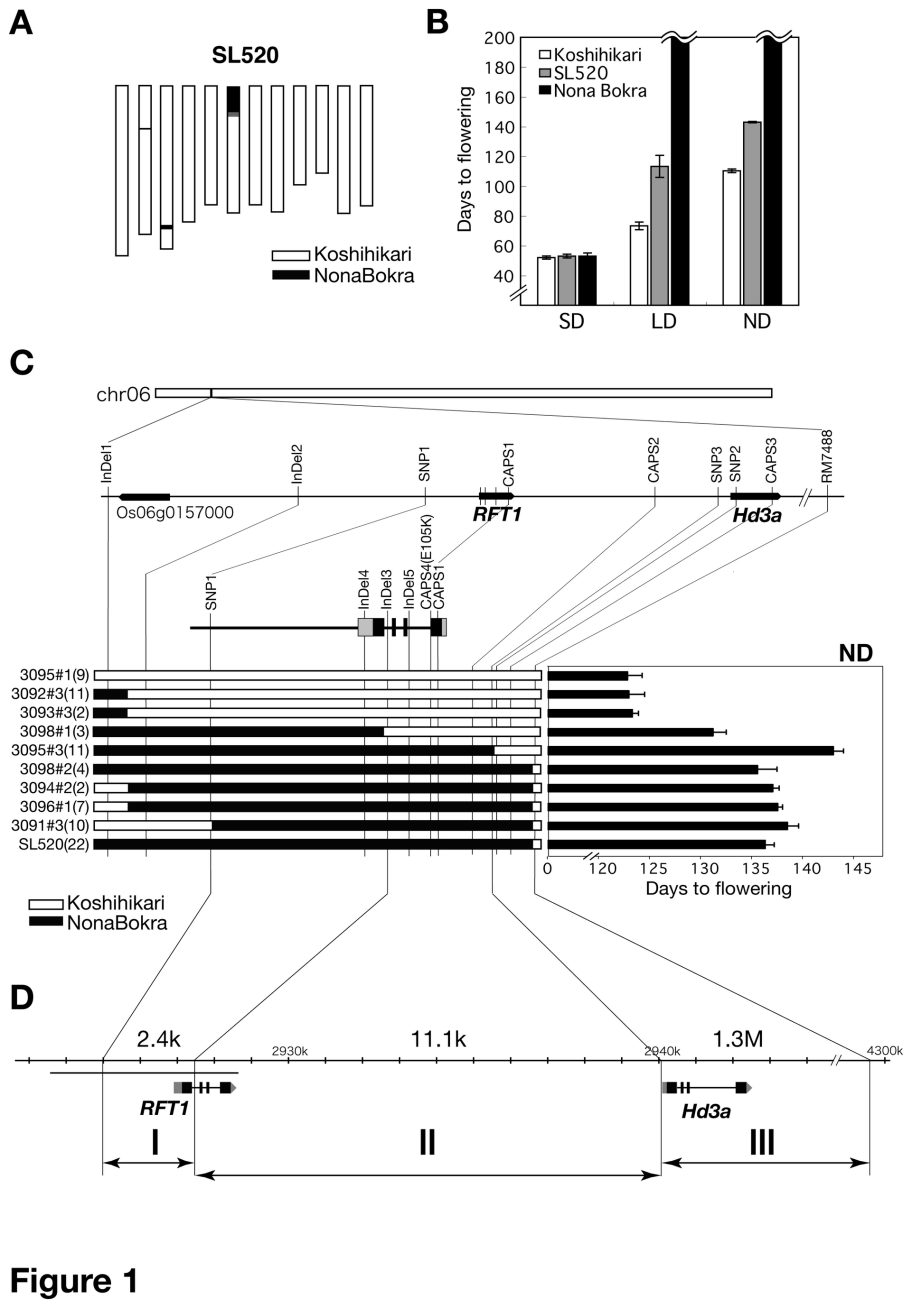
Comparison of SL520 with its parent lines Koshihikari and Nona Bokra, and delimitation of the candidate genomic regions for *RFT1* and *Hd3a*. (A) Graphical genotype of SL520. The white and black regions denote the regions homozygous for Koshihikari and Nona Bokra alleles, respectively. (B) Flowering time of SL520, Koshihikari and Nona Bokra under short-day (SD:10L/14D), long-day (LD:14.5L/9.5D) and natural field (ND) conditions in Tsukuba, Japan in 2009. Each bar represents mean ± SD (n = 10). (C) Delimitation of the candidate genomic regions for *RFT1* and *Hd3a*. Left: Graphical genotypes of the *RFT1* and *Hd3a* regions in 10 lines, in which recombination occurred between InDel1 and RM7488. Right: Days to flowering under ND conditions. Each bar represents mean ± SD. The number of plants is indicated in parentheses. (D) Division of the *RFT1*/*Hd3a* region into three subregions. The 2.4-kb, 11.1-kb and 1.3-Mb candidate regions were defined by linkage analysis. The effect of the Nona Bokra allele on flowering time is shown below each region. The underlined 5-kb (chr. 6: 2,923,569–2,928,437; see Figure S4) fragment was used for complementation analysis.

To identify the causal gene(s) on the short arm of Chr. 6 that are involved in a delay in flowering time, we performed high-resolution mapping of a QTL associated with flowering time. We selected the chromosome segment substitution line SL520 [56], which contains an introgressed segment on Chr. 6 (including the target QTL region) from cv. Nona Bokra in the genetic background of cv. Koshihikari (Figure 1A). SL520 showed late flowering under LD and ND conditions, but not under SD conditions (Figure 1B), which suggested that this line can be used to develop a population for QTL mapping on the short arm of Chr. 6. We used a candidate gene approach with the F_2_ progeny from a cross between Koshihikari and SL520. On the basis of the previous studies [52], we assumed that *RFT1* and *Hd3a* could be candidates for the QTL. Out of 2,750 F_2_ plants, we selected those with recombination in or near *RFT1* and *Hd3a*, and scored their flowering time (Figure S1). As we expected, some plants showed association between the genotype of the *RFT1/Hd3a* region and flowering time. But despite carrying the Nona Bokra segment in the short arm of Chr. 6, some plants showed early flowering under ND conditions (Figure S2A). This indicated that a Nona Bokra segment at a position other than the short arm of Chr. 6 affected flowering time in the F_2_ population. Therefore, we surveyed the whole genome genotype by using 1001 SSR markers (Table S1) to define the genetic background of SL520 in detail, and found two additional small Nona Bokra segments in Chr. 2 and 3 (Figure S2B). We investigated the genotypes of these two regions using F_2_ populations. Genetic interaction between the *RFT1/Hd3a* region and the small segment in Chr. 3 was found under ND and LD conditions (Figure S2C, D). The position of the small segment in Chr. 3 was consistent with *Hd16* [18]. The presence of the functional *Hd16* allele from Nona Bokra made it possible to observe the effect of *RFT1/Hd3a* (Figure S2C, D). Thus, *Hd16* had to be fixed for the Nona Bokra allele to enable fine mapping of the *RFT1/Hd3a* region. Ten F_2_ plants with homozygous *Hd16* and the recombinant chromosome within *RFT1* and *Hd3a* region were selected (Figure S1). In ten F_3_ lines, we selected homozygous plants and measured their flowering time under ND conditions (Figure 1C). The flowering time was categorized into four classes: similar to the control line (3095#1), and flowering 10, 15 and 20 days later than the control line (Figure 1C). Similar flowering time segregation was also observed under LD conditions (Figure S3). Therefore, we suggested that at least three genomic regions in *RFT1* and *Hd3a* are likely to be involved in late flowering in Nona Bokra. Region I (2.4 kb) was defined by two DNA markers, SNP1 and InDel3, and included the promoter region (2,283 bp from the transcription start site) and the 1^st^ exon of *RFT1*. Region II (11.1 kb) was defined by InDel3 and SNP3, and included the 1^st^ intron of *RFT1* and the entire *Hd3a* promoter region (−30 bp from ATG). Region III (1.3 Mb) was defined by SNP3 and RM7488, and included the entire *Hd3a* coding region (Figure 1D).

### Nona Bokra *RFT1* is mainly responsible for late flowering or lack of flowering under LD conditions

**Figure 2:**
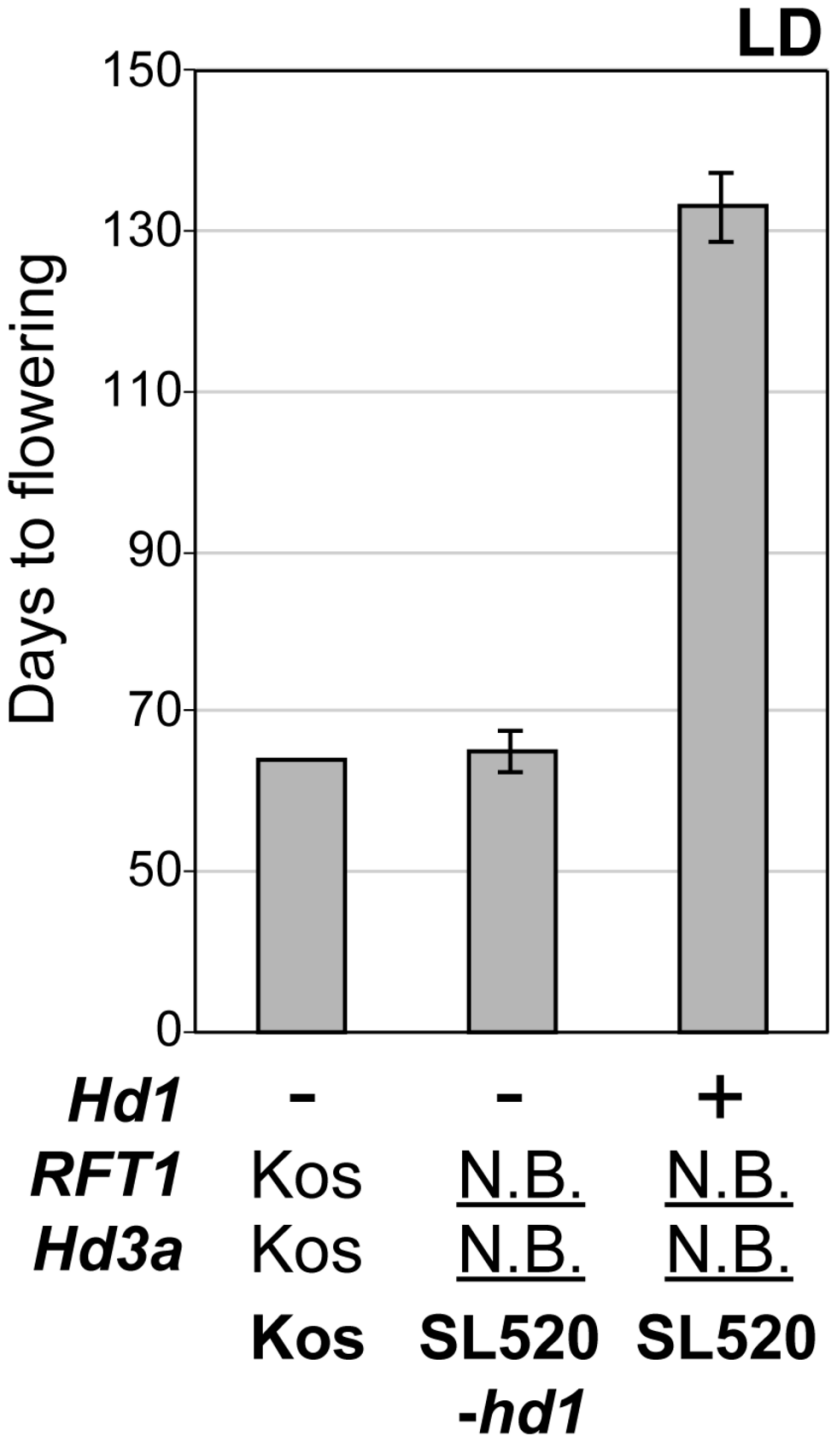
Genetic interaction between *Hd1* and the *RFT1/Hd3a* region under LD conditions. Days to flowering of Koshihikari, SL520-*hd1* and SL520 under LD (14.5L/9.5D) conditions. *Hd1, RFT1* and *Hd3a* genotypes are shown. *Hd1*+ and - indicate functional or nonfunctional allele. Kos and N.B. indicate Koshihikari and Nona Bokra allele. Each bar represents mean ± SD (n = 5).

Among the three predicted regions within the *RFT1*/*Hd3a* region, Regions I and II of the Nona Bokra allele delayed flowering compared to the Koshihikari allele, but Region III caused earlier flowering (Figure 1C, S3). As *RFT1* and *Hd3a* encode florigens, we assumed that delayed flowering was due to a malfunction of one of them. We assessed whether *Hd3a* was functional by measuring flowering time in a nonfunctional *Hd1* background. To develop the necessary line (SL520-*hd1*), SL520 was crossed with a nonfunctional *Hd1* near-isogenic line (Figure S1). Whereas late flowering of SL520 could be observed in a functional *Hd1* background, the flowering time of SL520-*hd1* was almost the same as that of Koshihikari (Figure 2). This indicated that the Nona Bokra Hd3a protein had normal function. Furthermore, sequence analysis of the entire coding and regulatory *Hd3a* regions (8 kb) showed that they were identical to those in Kasalath (data not shown). The *Hd3a* allele of Kasalath is functional, and causes earlier flowering than the Nipponbare allele (which is identical to that of Koshihikari) [20]. Thus, we concluded that the causal gene(s) for late flowering of SL520 are in the *RFT1* region.

**Figure 3:**
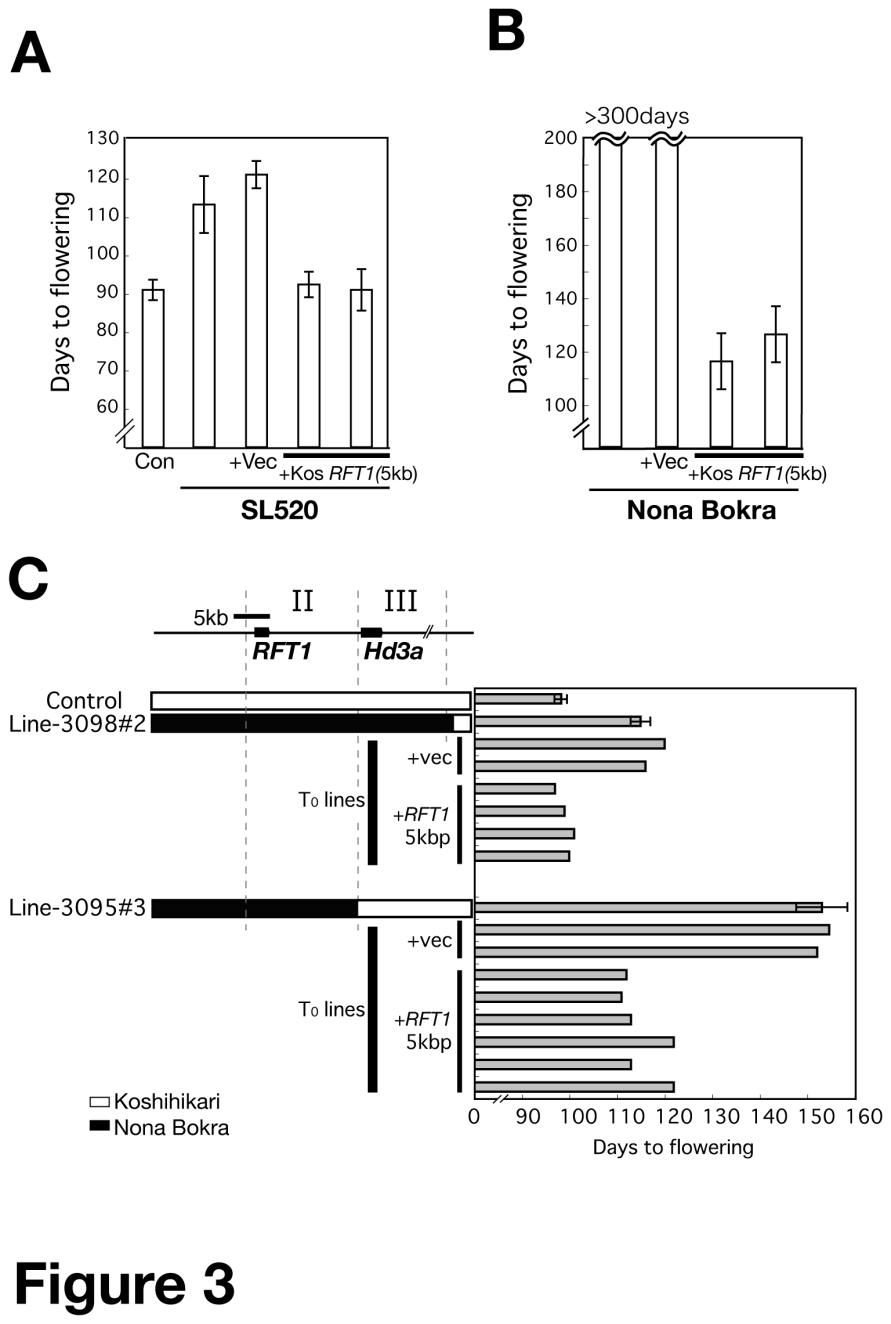
Genetic complementation test and effect of genetic background. (A, B) Flowering time of control (Con, see Figure S1), SL520 (A) or Nona Bokra (B), and T_2_ plants harboring the empty vector (+Vec) or the Koshihikari *RFT1* genomic fragment (+*KosRFT1* 5 kb; see Figure 1D) in SL520 (A) or Nona Bokra (B) under LD conditions. Each bar represents the mean ± SD (n = 3–5). (C) Genetic complementation test using F_3_ lines derived from a cross between Koshihikari and SL520 (Figure S1) under LD conditions. Graphical genotypes of *RFT1*/*Hd3a* regions are indicated. Lines 3098#2 and 3095#3 have Nona Bokra and Koshihikari *Hd3a* homozygous alleles, respectively. For transgenic lines (T_0_), each bar represents an individual plant. Control and genotypes of other regions are shown in Figure S1.

To examine the function of *RFT1*, we carried out a complementation analysis by introducing the *RFT1* genomic region (5 kb) of Koshihikari into SL520 and Nona Bokra backgrounds. T_2_ plants homozygous for a transgene containing the *RFT1* genomic region showed early flowering (similar to Koshihikari) compared to the vector-transformed control plants (Figure 3A). Plants carrying the Koshihikari *RFT1* genomic region in the Nona Bokra background could flower (after 120 days) under LD conditions (Figure 3B). These results indicated that the causal gene for late flowering was located in Regions I and II, and thus could be *RFT1* (Figure 1D). In order to test the genetic effect of Region III, we transformed two progeny lines, 3098#2 and 3095#3 (selected from the cross between SL520 and Koshihikari, Figure S1) with Koshihikari *RFT1*. Both *RFT1* and *Hd3a* regions were of Nona Bokra origin in 3098#2, whereas in 3095#3 only the *RFT1* region was from Nona Bokra (Figure 3C). Both 3098#2 and 3095#3 lines transformed with Koshihikari *RFT1* showed earlier flowering than respective vector-transformed controls, but 3095#3 showed later flowering than 3098#2. These results demonstrate that Koshihikari *RFT1* functions to promote flowering (Figure 3). Furthermore, the flowering time difference between 3098#2 and 3095#3 indirectly suggests that genomic Region III from Koshihikari is associated with late flowering, that *RFT1* (Regions I and II) from Nona Bokra is involved in late flowering, and that Region III from Nona Bokra is involved in early flowering.

### A single amino acid substitution (E105K) in *RFT1* causes a defect in promoting flowering

**Figure 4:**
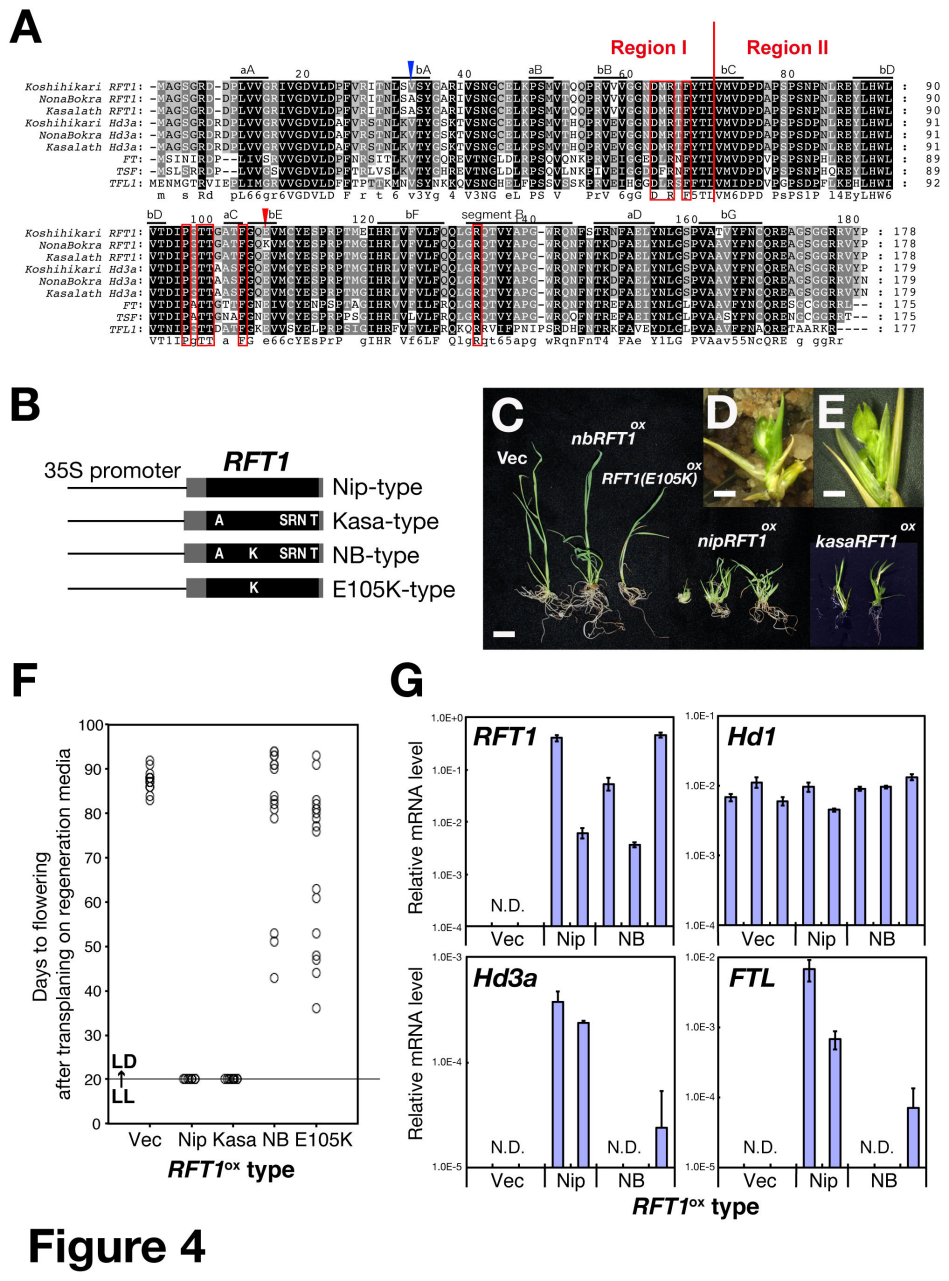
Amino acid sequences of RFT1 and Hd3a and phenotypes of RFT1 overexpressors. (A) Comparison of amino acid sequences of rice and Arabidopsis FT-like proteins. Rice RFT1 and Hd3a (from three cultivars as indicated), and Arabidopsis FT, TSF and TFL1 are shown. Conserved amino acids are shaded in black, dark gray or light gray depending on the level of conservation. The blue and red arrows indicate the V33A and E105K sites, respectively. The red boxes indicate the binding interface with 14-3-3 protein [57]. (B) 35S:*RFT1* constructs. *RFT1* alleles used: Nip, Nipponbare; Kasa, Kasalath; NB, Nona Bokra. E105K, Nipponbare allele with the introduced point mutation. Black and gray boxes denote ORF and UTR, respectively. Amino acids differing from those in Nipponbare are shown as white letters. (C) Regenerated plants (cv. Nipponbare) transformed with 35S:*RFT1* constructs or with the empty vector (Vec). Bar = 1 cm. (D, E) 35S:*nipRFT1* (D) and 35S:*kasaRFT1* (E) plants at higher magnifications. Bar = 2 mm. (F) Flowering time of 20 T_0_ plants overexpressing each of the 35S:*RFT1* constructs or vector control under LL (continuous light) and LD conditions. Day 0 corresponds to the date of transplanting onto regeneration media under LL conditions. On day 20, counting of the flowering plants was started, and plates were transferred to LD. Each open ellipse indicates an individual plant. (G) Expression levels of *RFT1*, *Hd3a*, *FTL* and *Hd1* expression in T_0_ plants transformed with the 35S:*RFT1* constructs or vector under LL conditions. N.D. indicates that transcripts were not detected. Samples were taken 20 days after transplanting onto regeneration media. Samples from five plants were mixed together. Each bar represents the mean ± SD (technical replicates n=3). Expression values were plotted on a log_10_ scale. *Ehd1* was not detected (data not shown).

To further define the functional nucleotide polymorphisms (FNPs) in Regions I and II, we compared the deduced amino acid sequences of Koshihikari, Kasalath and Nona Bokra RFT1 with those of FT-like proteins of *Arabidopsis* , and found that six amino acids are different between Koshihikari and Nona Bokra. Among them, one substitution, E105K, was observed only in Nona Bokra RFT1, but not in any other proteins, including *Arabidopsis* FT-like proteins (Figure 4A). To verify this candidate FNP, we developed Nipponbare (*nipRFT1ox*), Kasalath (*kasaRFT1ox*) and Nona Bokra (*nbRFT1ox*) lines overexpressing *RFT1* (Figure 4B). As *RFT1* nucleotide sequences were identical in Koshihikari and Nipponbare (Figure S4), we used Nipponbare cDNA to make wild-type and artificially mutated (E105K) *RFT1* genes under control of the constitutive 35S promoter (Figure 4B). T_0_ transgenic *nipRFT1ox* and *kasaRFT1ox* plants showed extremely early-flowering phenotypes in a growth chamber even under continuous light (LL) conditions (Figure 4C–F), whereas *nbRFT1ox, nipRFT1*(*E105K*)*ox* and control plants did not (Figure 4F). Some *nbRFT1ox* and *nipRFT1*(*E105K*)ox lines flowered earlier than the control lines under LD conditions (Figure 4F). In addition, *nipRFT1ox* plants had increased expression of other *FT*-like genes, *Hd3a* and *FTL*, despite the absence of *Ehd1* expression (data not shown) and no changes in *Hd1* expression under LL conditions (Figure 4G). This effect was less pronounced in the case of *nbRFT1ox* compared to *nipRFT1ox* plants. These results demonstrate that *nbRFT1* and *nipRFT1*(*E105K*) are almost all functionally defective, and suggest that the E to K substitution is the main reason for the functional defect and for the causal variation in region II.

We also evaluated causal variation in Region I. Region I contained the 1^st^ exon of *RFT1* (Figures 1D, 4A), and one nonsynonymous substitution leading to the V33A variation between Koshihikari (V) and Nona Bokra (A) (Figure 4A, B). However, Kasalath also had A in this position (Figure 4A, B), and *KasaRFT1* could promote flowering (Figure 4C, F); therefore, causal variation in Region I was unlikely to be due to this substitution. This suggests that the causal variation in Region I is in the regulatory region of *RFT1*.

### Expression patterns of *RFT1* and *Hd3a* under SD, LD and ND conditions

The flowering time correlates with the expression levels of *Hd3a* under SD, but not *RFT1* [23]. Therefore, we re-examined the relationship between flowering time and the expression levels of *RFT1* and *Hd3a* not only under SD, but also under LD and ND conditions using 24 cultivars (Figure S5A–D and Table S2). The expression of both *RFT1* and *Hd3a* correlated with flowering time under SD (*R* ^2^ = –0.46 and -0.58, respectively), LD (*R* ^2^ = –0.69 and -0.44) and ND (*R* ^2^ = –0.64 and -0.51) conditions (Figure S5D). Nona Bokra showed low expression levels of *RFT1* and *Hd3a* under LD and ND conditions, and the correlation coefficient increased when the data for Nona Bokra under ND conditions were removed (Figure S5C, D).

**Figure 5:**
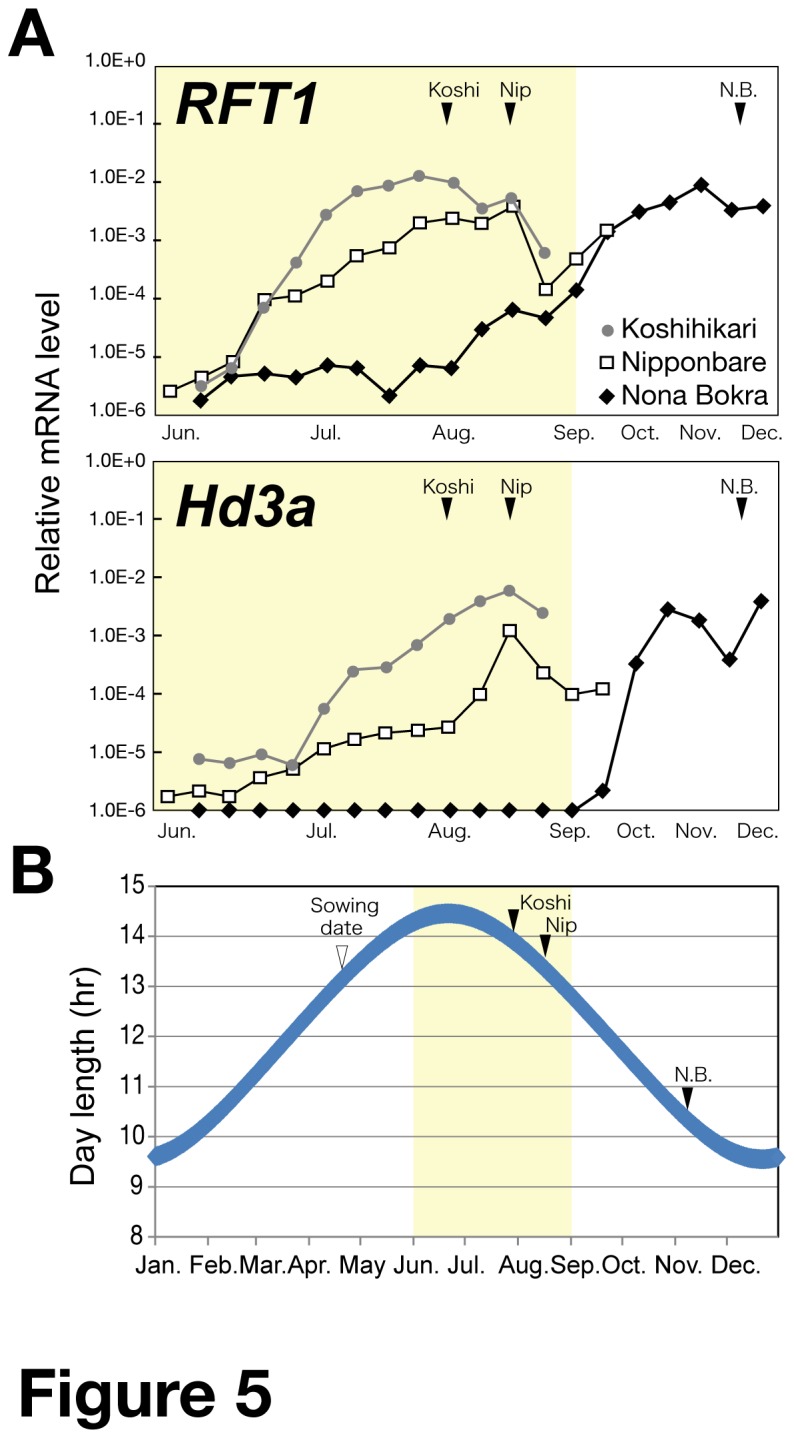
The mRNA levels of *RFT1* and *Hd3a* in Koshihikari, Nipponbare and Nona Bokra during the growing period in a natural field. (A) Expression levels of *RFT1* and *Hd3a* in top leaves. Expression values were plotted on a log_10_ scale. Black triangles indicate the flowering date for each cultivar. (B) Changes in daylength at the growing location. Open triangle indicates the sowing date for each cultivar. Yellow boxes represent the period of long-day conditions (>13 h daylength).

To investigate how *RFT1* and *Hd3a* are regulated in Nona Bokra, we grew Nona Bokra, Koshihikari and Nipponbare (as a control) in a paddy field (ND conditions), and sampled their leaves from June to flowering time: late July (Koshihikari), early August (Nipponbare) and mid-November (Nona Bokra) (Figure 5). In Koshihikari and Nipponbare, *RFT1* and *Hd3a* mRNA levels increased gradually from mid-June despite relatively long days under ND conditions (Figure 5). These expression patterns were supported by microarray data in the RiceXPro database [58] (Figure S6). In Nona Bokra, *RFT1* levels increased very slowly from August to November (the cultivar’s flowering time). Although high levels of *RFT1* transcript were detected in Nona Bokra in early October, flowering was not observed until the end of November (Figure 5). *Hd3a* expression in Nona Bokra was strongly repressed from June to early September, and was induced when the daylength became less than 13 h. *Hd3a* expression patterns clearly showed photoperiodic response under ND conditions in Nona Bokra (Figure 5). These results revealed that expression patterns of *RFT1* and *Hd3a* in Nona Bokra were very different from those of Koshihikari and Nipponbare under ND conditions.

### A region in the short arm of chromosome 6 affects *RFT1* and *Hd3a* expression patterns

**Figure 6:**
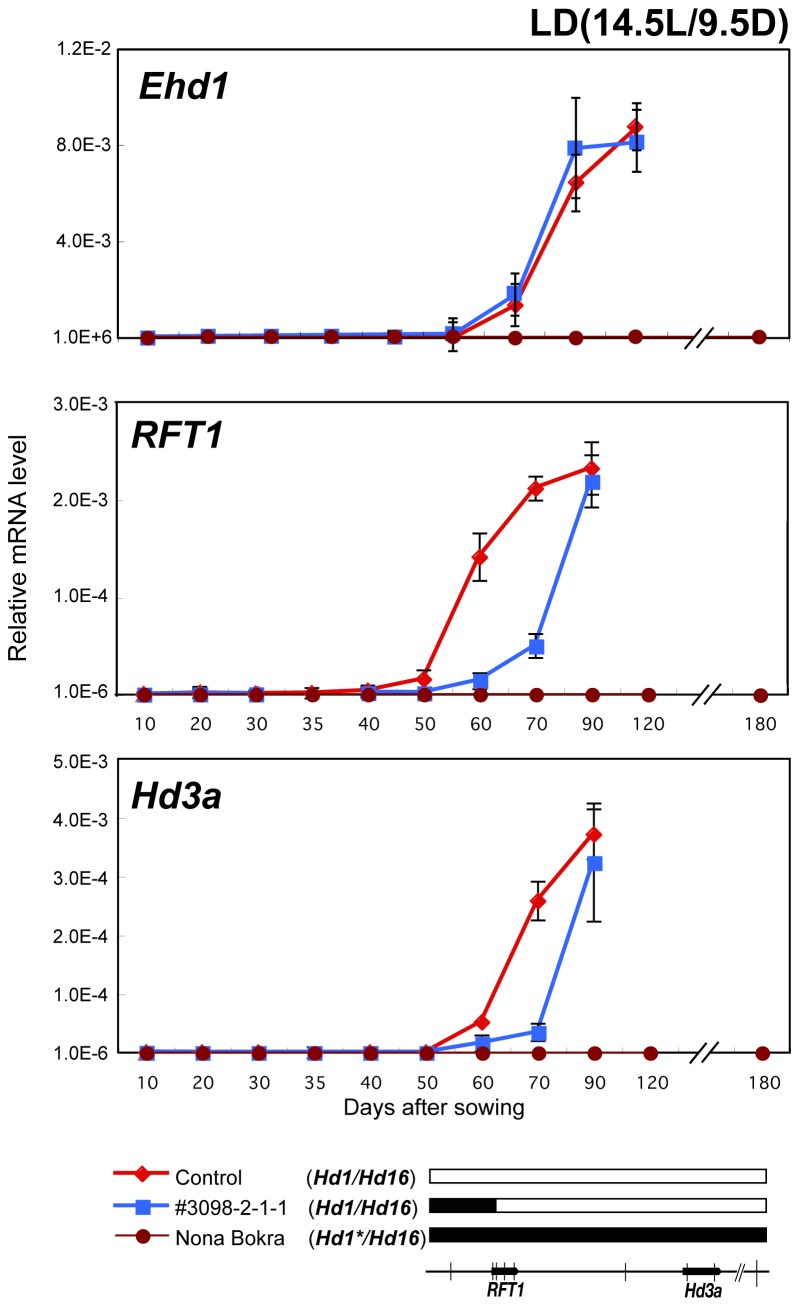
mRNA levels of *Ehd1*, *RFT1* and *Hd3a* in control, #3098-2-1-1 and Nona Bokra growing under LD conditions. Graphical genotypes around *RFT1* and *Hd3a* are shown below (for graphical genotypes of the whole genomes, see Figure S1). White and black boxes represent Koshihikari and Nona Bokra origin, respectively. All lines have functional *Hd1* and *Hd16*. Hd1* indicates strong allele [52]. *Ehd1*, *RFT1* and *Hd3a* expression was below the detection limit in Nona Bokra.

We investigated the effect of the short arm of Chr. 6 on *RFT1* and *Hd3a* expression under LD conditions. As *RFT1* and *Hd3a* expression is regulated by several flowering time genes, detecting expression differences by regulatory variation was very difficult. Therefore, the SL line SL520-#3098 (with Nona Bokra allele from the distal short arm of Chr. 6 to the 1^st^ intron of *RFT1*) derived from the cross between SL520 and Koshihikari was used for the expression analysis (Figure 6, S1). In spite of similar expression levels of *Ehd1*, the induction of *RFT1* and *Hd3a* expression in SL520-#3098 was delayed in comparison with the control line (Figure 6), suggesting that the short arm of Chr. 6 affects the induction of *RFT1* and *Hd3a* expression. Furthermore, in Nona Bokra, *Ehd1* expression was not detected under LD conditions (Figure 6). This result indicated that *Ehd1* was repressed by other flowering time genes in Nona Bokra, but not in Koshihikari. As a consequence, *RFT1* and *Hd3a* were not induced, and no flowering occurred in Nona Bokra under LD conditions (Figure 1B).

### Nona Bokra and similar haplotypes of *RFT1* from a rice core collection are strongly associated with extremely late flowering under ND and LD conditions

**Figure 7:**
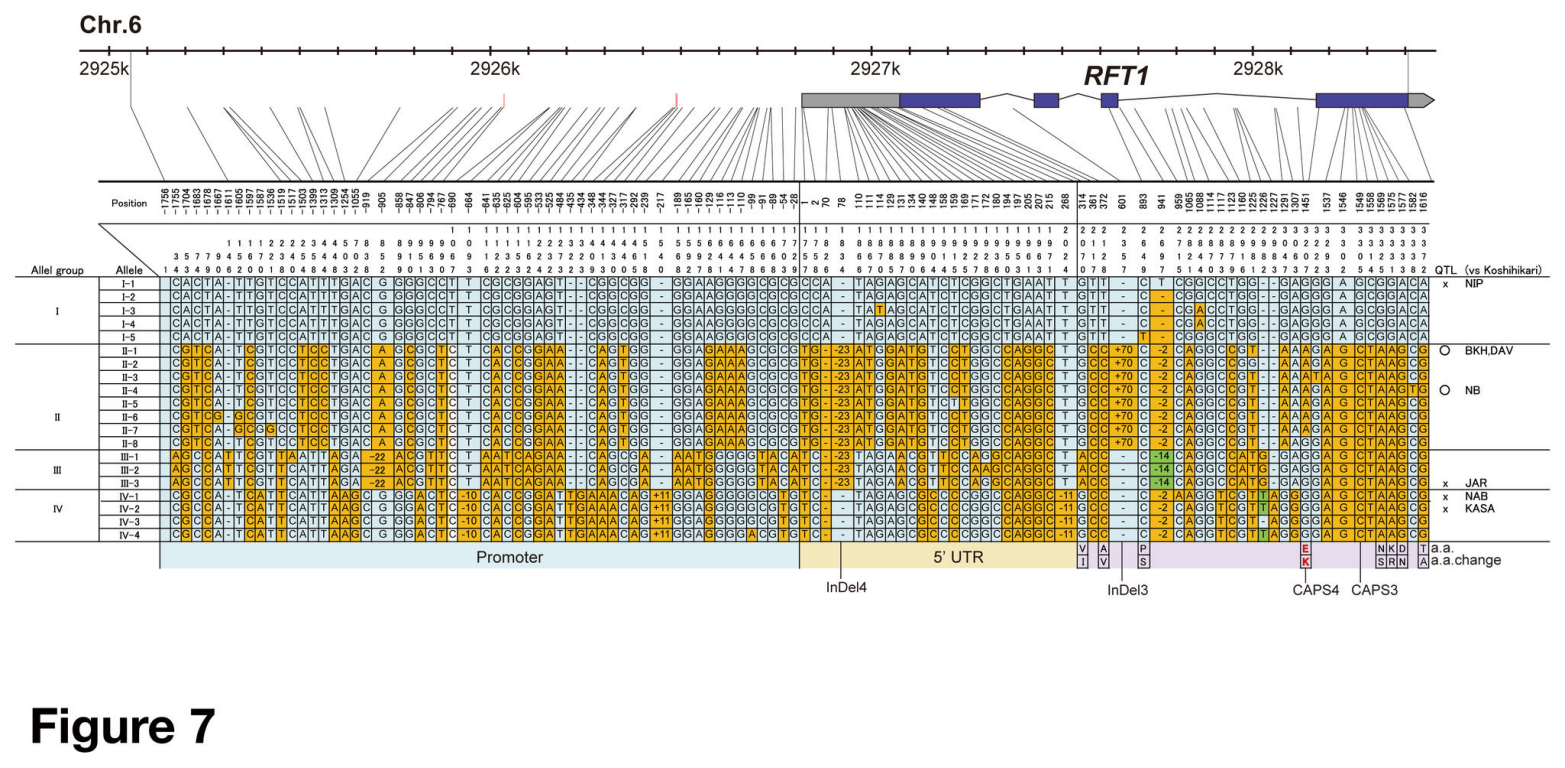
RFT1 allele haplotypes. RFT1 gene regions of plants from the rice core collection were compared with those of Nipponbare (NIP). BKH, Bhei Khei; DAV, Davao; NB, Nona Bokra; JAR, Jarjan; NAB, Naba; KASA, Kasalath. Site numbering and physical positions are based on the Nipponbare sequence (RAP-DB build 5.0 [85]). Nucleotide substitutions are highlighted in orange. Red vertical bars indicate ARR1 binding elements. Gray and purple boxes represent UTRs and ORF of RFT1, respectively. Amino acid changes are indicated below the alignment. O or X on the right indicate whether a QTL was detected or not, respectively, in this region in the population derived from the cross between Koshihikari and particular cultivars.

The available data suggested that *RFT1* is involved in extremely late flowering [50-52]. To evaluate this hypothesis, we investigated the nucleotide polymorphism in the regulatory and coding regions of *RFT1* (3.5 kb) among 66 cultivars from the rice core collection [59,60]. We identified 20 haplotypes and classified them into four groups (I to IV) according to their similarity (Figure 7). Group I contained *indica* and *japonica* (including Koshihikari) cultivars (Figure S7). Most *japonica* cultivars had the group I allele. Group II contained only *indica* cultivars (including Nona Bokra). Groups III and IV were mixtures of *indica* and *japonica* cultivars (Figure S7). We found eight nonsynonymous substitutions in the *RFT1* coding sequence in the rice core collection. Nona Bokra *RFT1* (group II-4) had six nonsynonymous substitutions, including the E105K FNP site. Two other substitutions were found in groups I and III. Group I-5 RFT1 (cv. Khau Mac Kho [KMK]) had a P160S substitution (Figure 7). A flowering time QTL was not detected in *RFT1/Hd3a* regions between Koshihikari and cv. KMK [51]. Group III RFT1 had a V12I substitution. Cv. Badari Dhan (BAD) in group III had functional *Hd1*, but its flowering time was moderately increased under LD (80 days) and ND (107 days) conditions (Table S3). In addition, no flowering time QTL was detected in the *RFT1/Hd3a* regions between Koshihikari and cv. Jarjan (in group III-3) (Figure 7, S8). These data suggest that the *RFT1* allele encoding the P160S and V12I substitutions has normal function.

In the *RFT1* regulatory region (1,756 bp upstream of the transcription start site), we found three ARR1 binding sites (Figure 7, S5). The ARR1 binding element is a candidate site for GARP, a DNA binding motif of Ehd1 [6], but there were no polymorphisms in the ARR1 binding sites in the rice core collection. We sequenced an additional regulatory region (−3,254 to −1,755 bp) of *RFT1* in Nona Bokra, and found three additional ARR1 binding sites and three CCAAT boxes (Figure S4). DTH8 can potentially bind the CCAAT boxes in the *RFT1* regulatory region [9-12], but there were no polymorphisms in ARR1 binding and CCAAT box sites in Nona Bokra. We found 43 SNPs and one insertion in region I in Nona Bokra, but no polymorphism was found in recognizable potential *cis*-element sites (Figure S4).

**Figure 8:**
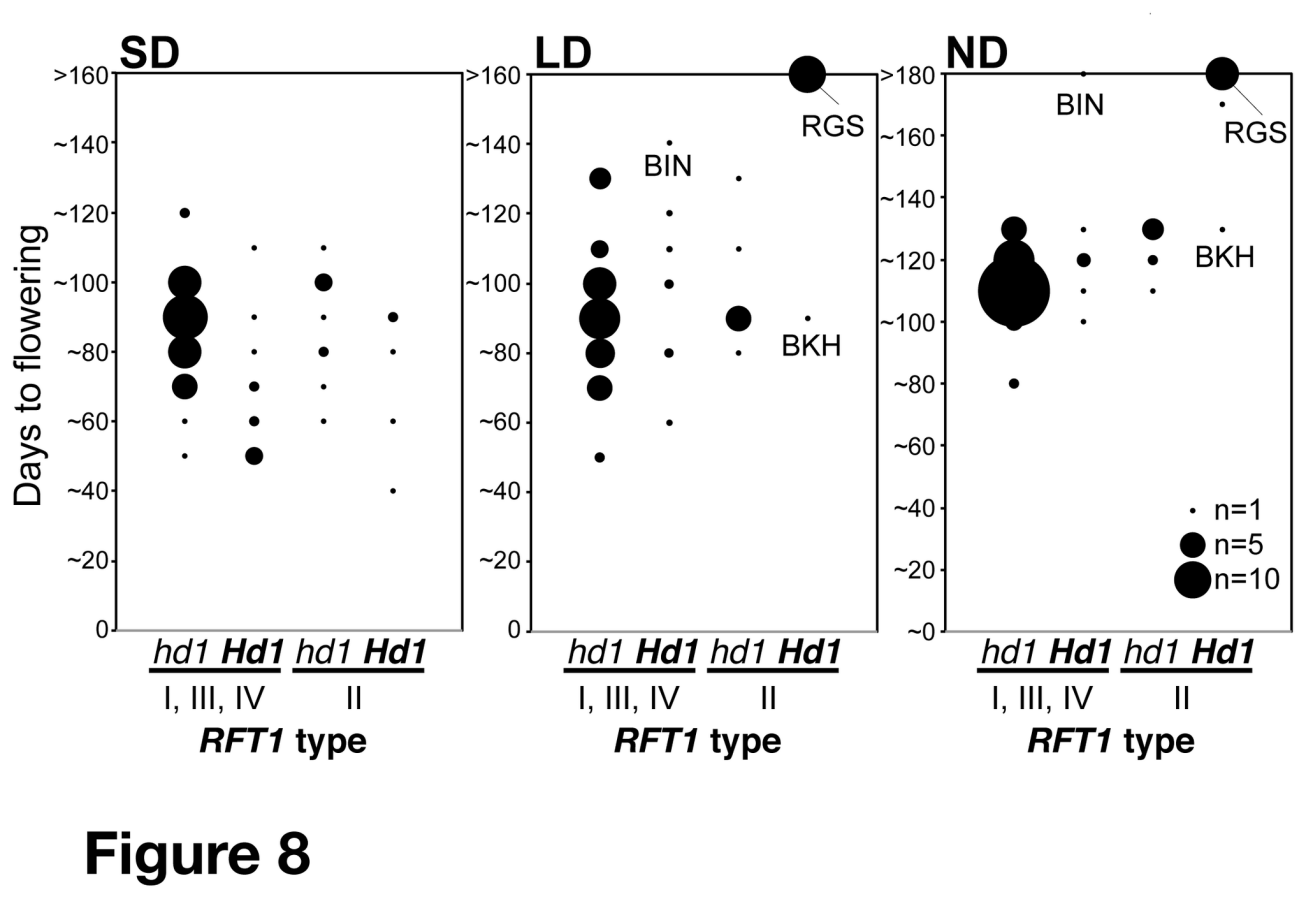
Flowering time of plants from the core collection under SD, LD and ND conditions. Accessions were divided into four groups by *RFT1* haplotypes and *Hd1* function (functional *Hd1* is shown in bold). *RFT1* haplotypes were divided into two groups, candidate functional (groups I, III and IV) or defective (group II) haplotypes. Accessions were grouped according to flowering time (in 10-day steps). The diameter of the black circles reflects the number of accessions. BIN, Bingala; BKH, Bei Khei; RGS, Radin Goi Sesat. The distributions of flowering time of group II with functional *Hd1* deviated from other distributions under LD and ND conditions (*P*<0.01, Kolmogorov-Smirnov test), but not under SD conditions (*P* = 0.135).

We investigated the relationships between *RFT1* haplotype groups and flowering time. As the *RFT1* phenotype appeared in a functional *Hd1* background, cultivars were divided into two categories: *hd1* (nonfunctional) or *Hd1* (functional) (Figure 8). Most cultivars with extremely late flowering under LD and ND conditions had group II *RFT1* and functional *Hd1*. This clearly indicated that group II *RFT1* has an important role in delaying flowering in the presence of functional *Hd1*. However, there were two exceptions: cv. Bei Khei (BKH) and cv. Bingala (BIN). BKH had functional *Hd1*, but showed early flowering under LD and ND conditions (Figure 8, Table S3). This suggested that BKH had a defect in a flowering time gene other than *Hd1. DTH8* could be responsible for this effect [51]. BIN had functional *Hd1* (the same type as BKH) and presumably functional *RFT1* (group IV), but it showed extremely late flowering under ND conditions (Figure 8). The *Hd3a* allele in BIN was of the Nona Bokra type. This indicated that BIN should have strong flowering suppressor gene(s) other than *Hd1*. Cv. Radin Goi Sesat (RGS) had functional *Hd1* (the same type as BKH and BIN), and showed extremely late flowering under ND conditions (Figure 8, Table S3). RGS did not have the E105K substitution, although the *RFT1* regulatory region was of the Nona Bokra type (II-8 on Figure 7), indicating that this regulatory region is involved in late flowering in RGS. Taken together, these results indicated that the group II haplotype of *RFT1* has an important role in ‘extremely’ late flowering under LD and ND conditions.

### Diversification of *RFT1* and *Hd3a* in rice

Nucleotide diversity is used to measure the degree of polymorphism within a population [61]. Sequence analysis of fragments from six cultivars and four wild rice species revealed that the nucleotide diversity was higher in *RFT1* than in *Hd3a* [50]. To expand this analysis, we additionally sequenced the coding regions of *RFT1* and *Hd3a* in cultivated and wild rice accessions (204 in total, including the accessions from the core collection, see above), and found 16 amino acid changes in RFT1 and 10 changes in Hd3a (Figure S9 and Table S3). No frameshifts or premature stop codons were found. The nucleotide diversity () of *RFT1* in the coding region and in the entire gene region was higher than that of *Hd3a* (Table 1), as found previously [50]. In addition, since defective *RFT1* was found in *indica*, we also analyzed and Watterson’s estimator () [62] within the two subspecies (*indica* and *japonica*). Although the value was similar among the two subspecies, the value of *RFT1* in *indica* was almost four times that in *japonica*, indicating the higher diversity in *RFT1*. The haplotype diversity of *RFT1* was similar to that of *Hd3a* in cultivated rice. The *RFT1* haplotype number was larger than that of *Hd3a* in the entire gene region, but smaller in the coding region. Spontaneous mutations may have occurred at particular sites in the *Hd3a* coding region, increasing the number of haplotypes. Mutations in *RFT1* were similar among the accessions, and the haplotype number appeared to decrease despite high nucleotide diversity (Table 1). Furthermore, we sequenced the entire coding regions of *RFT1* (7 species) and *Hd3a* (11 species) in wild *Oryza* species ( *O* *. glaberrima* [AA genome constitution], *O* *. barthii* [AA], *O* *. glumaepatula* [AA], *O. longistaminata* [AA], *O* *. meridionalis* [AA], *O* *. punctata* [BBCC], *O* *. minuta* [BBCC], *O* *. officinalis* [CC], *O* *. alta* [CCDD], *O, australiensis* [EE] and *O* *. brachyantha* [FF]). We found a frameshift mutation in *RFT1* in *O* *. glaberrima* and *O* *. barthii* , and a premature stop codon in *O* *. meridionalis* ; no frameshift mutations or premature stop codons were found in *Hd3a*. Nonsynonymous substitutions were found in both *RFT1* (42 sites) and *Hd3a* (27 sites) (Figure S9). If RFT1 and Hd3a were important as florigens, their amino acid sequences would be highly conserved in *Oryza* species. Do the differences in the extent of nucleotide diversity and in the number of nonsynonymous substitutions indicate pseudogenization of *RFT1*?

**Table 1:** Nucleotide diversity and divergence in each region of *RFT1* and *Hd3a* gene.

			No. of accessions	Number of sequence	Number of sites*	S	π	θ	number of Haplotypes	Haplotype diversity
***RFT1***	Entire region	*O. sativa*	141	3,427	2,876	106	0.0219	0.0079	18	0.7336
		*indica*	75	3,427	3,214	103	0.0114	0.0080	16	0.7996
		*japonica*	66	3,427	2,944	75	0.0035	0.0082	6	0.3263
		*O* *. rufipogon*	16	3,376	3,275	132	0.0230	-	15	0.9917
	CDS	*O. sativa*	150	537	537	13	0.0188	0.0067	5	0.6822
		*indica*	83	537	537	13	0.0118	0.0075	5	0.7091
		*japonica*	67	537	537	12	0.0033	0.0079	4	0.2451
		*O* *. rufipogon*	28	537	537	22	0.0229	-	10	0.8598
***Hd3a***	Entire region	*O. sativa*	141	3,184	3,003	39	0.0049	0.0068	14	0.7479
		*indica*	75	3,184	3,004	37	0.0031	0.0062	12	0.7560
		*japonica*	66	3,184	3,128	32	0.0024	0.0047	7	0.4186
		*O* *. rufipogon*	16	3,115	2,966	84	0.0129	0.0159	12	0.9667
	CDS	*O. sativa*	150	540	540	10	0.0048	0.0067	9	0.6299
		*indica*	83	540	540	8	0.0030	0.0060	7	0.4229
		*japonica*	67	540	540	7	0.0021	0.0047	6	0.3903
		*O* *. rufipogon*	28	540	540	17	0.0116	0.0194	15	0.9471

* Excluding sites with gaps/missing data S: Number of polymorphic sites π: Nucleotide diversity for synonymous. θ: Wattterson's estimator of the synonymous diversity.

To evaluate the effect of natural selection, we compared the evolutionary rates [61] of *RFT1* and *Hd3a*. Since the nucleotide diversity was higher in *RFT1* than in *Hd3a*, this implies that functional constraint was relaxed in *RFT1* after gene duplication. If so, the evolutionary rate in *RFT1* would have increased. Phylogenetic analysis and colinearity of genes around *RFT1* and *Hd3a* revealed that *BradHd3a* ( *Brachypodium* *distachyon* Bradi1g48830) and *SbHd3a* ( *Sorghum* *bicolor* Sb10g003940) are orthologs of *RFT1* and *Hd3a* [63-65]. We compared the evolutionary rates of *RFT1* and *Hd3a* by two methods. First, the Tajima’s relative rate test [66] showed no significant differences between *RFT1* and *Hd3a* in each lineage (χ^2^ test, *P*>0.05) (Table S4, S5). Even in Nona Bokra, no significant difference was observed. Second, the ratio of nonsynonymous substitutions per synonymous substitutions (dN/dS) was low (below 0.25) in both *BradHd3a* and *SbHd3a*. A scatter plot of dN/dS for *RFT1* and *Hd3a* in each lineage did not show any significant differences, even though the ratio was smaller in *RFT1* in some cultivars (Figure S10). Therefore, there was no clear difference in the evolutionary rates of *RFT1* and *Hd3a*, and indicating that they are under the same degree of functional constraint. Both genes are highly conserved in *Oryza* species.

**Figure 9:**
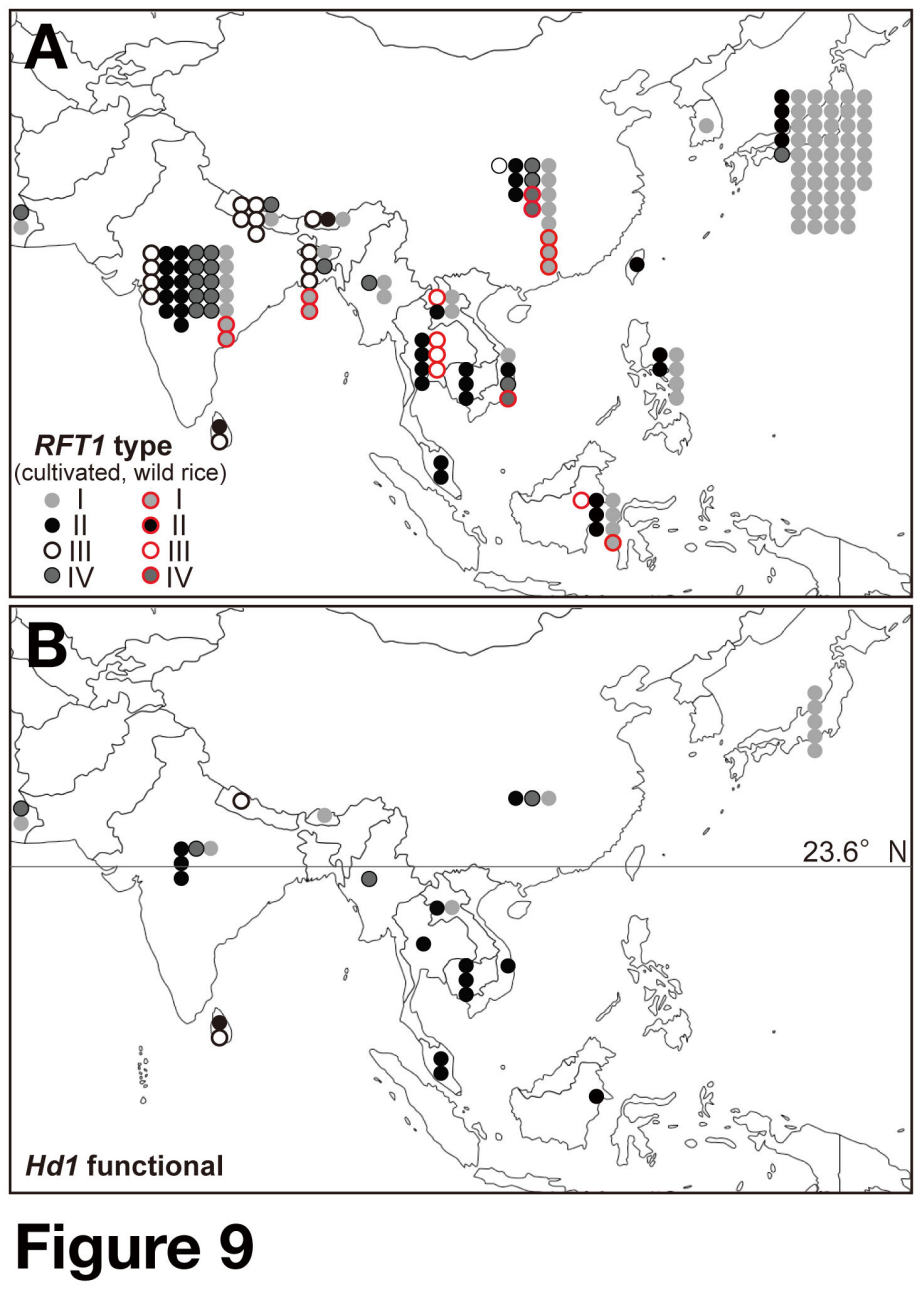
Geographic distribution of rice accessions and *RFT1* haplotype groups. (A) Distribution and *RFT1* haplotypes of 143 *O. sativa* and 16 *O* *. rufipogon* accessions. (B) The subset containing only accessions with functional *Hd1*. Black line indicates 23.6° north latitude. To the south of this line, maximum daylength is less than 13 h.

Haplotype group II *RFT1* alleles with E105K were found in *indica* cultivars and wild rice, but not in *japonica* cultivars (*indica* n = 40, *japonica* n = 0, *O* *. rufipogon* n = 1) (Table S3 and Figure S7). We investigated the relationship between *RFT1* haplotypes and the distribution of their areas of cultivation. The cultivars with group II *RFT1* are found in the whole of Asia, and accessions with group II *RFT1* in a functional *Hd1* background are found at lower latitudes (<23°60′N) (Figure 9).

### SNPs in E105K and the *RFT1* regulatory region have strong linkage disequilibrium with a flowering time–associated SNP

A genome-wide association study using 950 rice accessions from a global collection showed that the SNP most strongly associated with flowering time is located on chr. 6 (SNP chr6_2912415) [55]. We found that the physical distances from this SNP to ATG of *RFT1* and *Hd3a* were about 15 and 27 kb, respectively (Figure S11). Using Rice HapMap data [67], we investigated the linkage disequilibrium of SNPs in *RFT1* and *Hd3a* with the peak SNP (chr6_2912415) in global rice accessions (*O. sativa* n = 950). We found 25 SNPs in the *RFT1* region, and 21 SNPs in the *Hd3a* region in 217 cultivated rice accessions (Table S6). Most SNPs in the regulatory and coding regions of *RFT1* and *Hd3a* showed strong linkage disequilibrium with the peak SNP (*D'* > 0.90). The *D'* value for *RFT1* FNP(E105K) was 1.00 and that for *Hd3a* candidate FNP(P179N) [20] (chr6_2927179) was 0.93 (Figure S11). This indicates that *RFT1* and *Hd3a* regions are coinherited with the peak SNP. *RFT1* SNP(V33A) showed very high *r* ^2^ values (0.97), but this nonsynonymous substitution had no effect on the flowering time in the overexpression experiment (Figure 4). The *r* ^2^ value for *RFT1* FNP(E105K) was 0.38 and that for *Hd3a* candidate FNP(P179N) was 0.56 (Figure S11).

## Discussion

### The mechanism of extremely late flowering or lack of flowering in Nona Bokra under ND and LD conditions

In this study, we found that three regions (I to III), which include *RFT1* and *Hd3a*, are detected as flowering time QTLs, and we revealed the roles of each region. We found that Nona Bokra RFT1 has a functional defect because of the E105K mutation. We further showed that sequence variations in the regulatory region may also reduce *RFT1* function at the transcriptional level. We demonstrated that *RFT1* and *Hd3a* were never expressed in Nona Bokra, which never flowered under LD conditions (Figure 6), but transformation with *kosRFT1* restored flowering (Figure 3). This indicated that Koshihikari *RFT1* with its own regulatory region was induced normally in Nona Bokra under LD conditions. This suggested that the abnormal regulation of *RFT1* expression (Region I) is the major cause of the lack of flowering under LD conditions. In addition, the region from the distal end of Chr. 6 to the 1^st^ exon of *RFT1* (the Nona Bokra segment) delayed the induction of *RFT1* and *Hd3a* expression (Figure 6). Because the Nona Bokra segment on the distal end of Chr. 6 above the InDel2 marker did not affect flowering time (Figure 1C, D), the delay in *RFT1* and *Hd3a* expression might be caused by Region I (Figure 6). It is known that *RFT1* expression is regulated by chromatin modification [36]. Region I contains the H3K9 acetylation locus (5′-UTR), and the levels of H3K36 methylation are high at the *RFT1* locus [49]. In *Arabidopsis* , quantitative modulation of chromatin silencing through *cis* variation (nucleotide changes) in the *FLC* locus was reported [68]. These reports imply that the regulatory region of *RFT1* (Region I) is affected by chromatin silencing, which is in turn affected by variations in this region.

**Figure 10:**
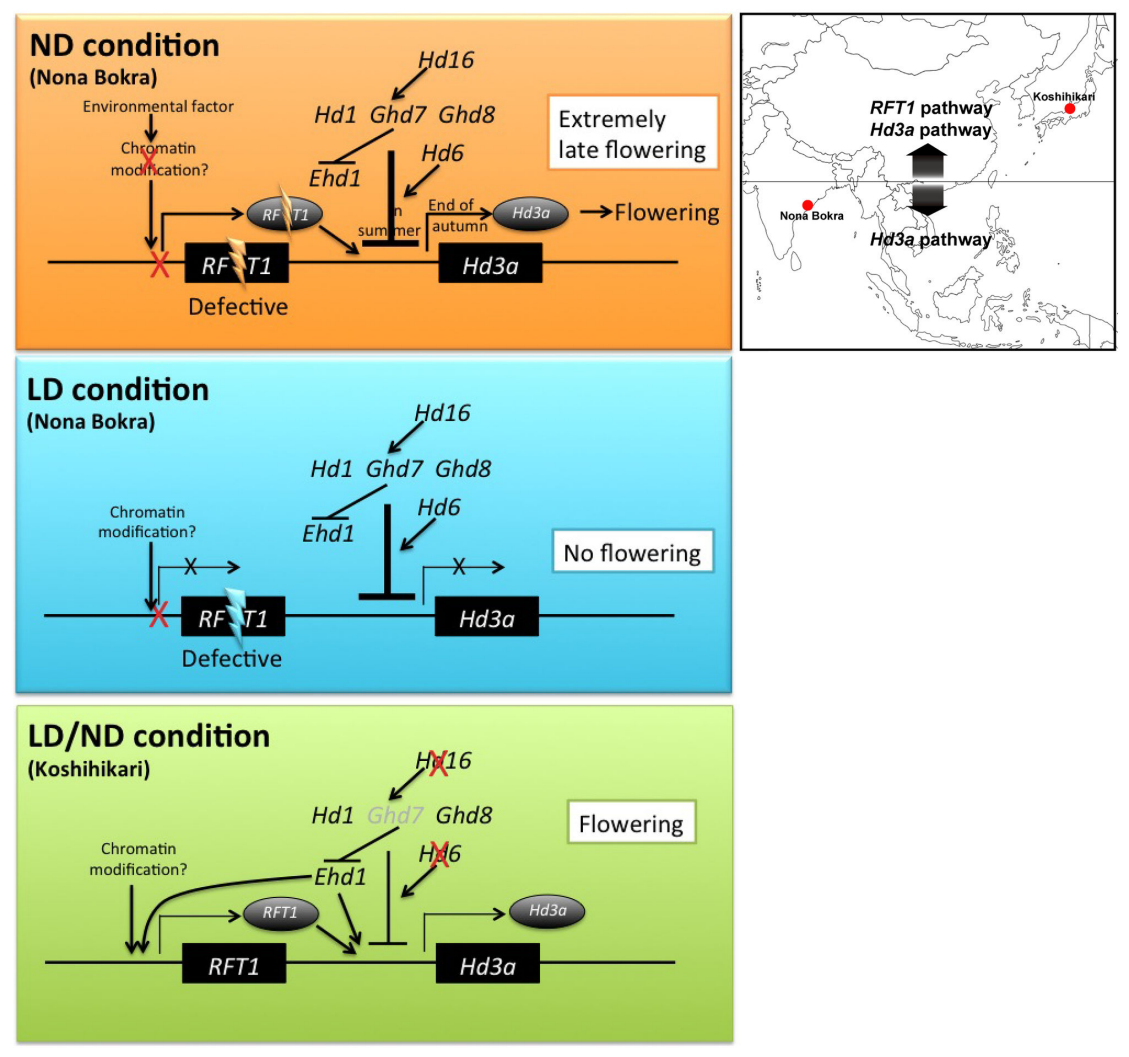
Schematic models of the flowering pathway in Nona Bokra and Koshihikari under ND and LD conditions. *RFT1* in Nona Bokra is defective, but the pathway that represses *Hd3a* expression is functional. Koshihikari has functional *RFT1*, but has defects (in *Hd6* and *Hd16*) in the pathway that represses *Hd3a*. Under ND conditions, *RFT1* begin to express from summer, but it cannot induce *Hd3a* expression in Nona Bokra. In autumn (short day conditions), *Hd3a* expression is induced and Nona Bokra can flower. Under LD conditions, *RFT1* expression does not occur, and *Hd3a* is repressed by flowering repressor genes, and Nona Bokra cannot flower. In Koshihikari, *Hd3a* and *RFT1* express under LD and ND condition, and it shows early flowering. Geographic location of the original cultivation regions of Koshihikari and Nona Bokra is shown on the right.

Under ND conditions, *RFT1* and *Hd3a* showed unique expression patterns. *RFT1* expression in Nona Bokra was induced in summer, but *Hd3a* was induced after the daylength became relatively short, which preceded flowering (Figures 5, 10). This indicates that *Hd3a* expression is entirely controlled by photoperiod in Nona Bokra. In contrast, in Koshihikari and Nipponbare, *Hd3a* was induced even under relatively long-day conditions (Figure 5). This induction can be explained by defective *Hd16* in Koshihikari [18], but not in Nipponbare. We found that high expression of functional *RFT1* can induce *Hd3a* expression without inducing *Ehd1* expression in Nipponbare (Figure 4G). Therefore, we suggest that *RFT1* plays an important role in regulation of *Hd3a* expression in photoperiod-sensitive varieties under LD conditions and during summer under ND conditions*.*

We found that functional *Hd1* and *Hd16* are necessary for late flowering in plants with defective *RFT1* (Figure 2, S2). These results indicate that when *Hd3a* expression is repressed, the *RFT1* effect appears. *Hd1, Hd2, Hd6* and *RFT1* regions were detected by QTL analysis of the difference in flowering time between Koshihikari and Nona Bokra [52]. *DTH8* and *Ghd7* were not detected in crosses between Koshihikari and Nona Bokra or between Koshihikari and Nipponbare [52,17]. Koshihikari has functional *Hd1*, *DTH8* and *Ghd7* (Nipponbare type), nonfunctional *Hd6* (Kasalath type), and a dominant *Hd2* allele (because the *Hd2* locus was not detected in QTL analysis in a cross between Koshihikari and Nipponbare, but was detected in a cross between Nipponbare and Kasalath [17,69]). Thus, Nona Bokra has strong flowering repression genes *Hd1, Hd2, Hd6*, *DTH8, Ghd7* [52] and *Hd16* (located near *Hd6*) [17,18]. Its non-flowering phenotype under LD may be caused by the defect in *RFT1* and by strong repression of *Hd3a* (Figure 10). This model can explain the following phenomenon: under LD conditions, cv. Norin 8 with *RFT1* suppressed by RNAi flowers extremely late, but does flower eventually [36]. Because Norin 8 has nonfunctional *Hd6* (*e3*) [15,16], the level of *Hd3a* repression in Norin 8 is weaker, which allows flowering under LD conditions.

We observed the effect of the *Hd3a*-containing locus (Region III) on flowering in the *nbRFT1* background under ND and LD conditions (Figures 1C, 3C, S3). The Nona Bokra Region III caused earlier flowering. The *Hd3a* nucleotide sequence (including promoter) is identical between Koshihikari and Nipponbare, and between Nona Bokra and Kasalath. The Koshihikari/Nipponbare and Nona Bokra/Kasalath types of *Hd3a* are functional, but the Kasalath allele (*KasaHd3a*) promotes earlier flowering than the Nipponbare allele [20], although the reported difference between Nipponbare and a near-isogenic line carrying *kasaHd3a* was small (5 days) under LD conditions [20]. In this study, in a defective *RFT1* background, the difference between line III (*nbRFT1/kosHd3a*) and SL520 (*nbRFT1/nbHd3a*) under LD conditions was larger (>20 days) (Figure S3). Therefore, we speculate that if the *RFT1* pathway is not functional, a small functional difference in the *Hd3a* (pathway) can be detected (Figure 10). Our data show that *RFT1* can regulate the expression of *Hd3a* and *FTL* (Figure 4G). The *RFT1* and *Hd3a* pathways are coordinated and can precisely control flowering time [48]. The first induced florigen initiates the flowering process (Figure 10). In Nona Bokra, *Hd3a* appears to be the only florigen gene: its repression under LD conditions causes extremely late flowering under ND conditions and no flowering under LD conditions.

### Functional divergence and pseudogenization of *RFT1* and *Hd3a*

We found that the duplication of the *RFT1* and *Hd3a* ancestor gene occurred after the divergence between rice and *Brachypodium* (about 40 Mya [70,71]). Since *RFT1* and *Hd3a* genes are present in all *Oryza* species (Figure S9), we assume that a single duplication event occurred before their divergence. The evolutionary rates were not significantly different between functional and defective *RFT1* (Table S4 and Figure S10), and the defective alleles accumulated very few mutations, suggesting a functional constraint on both genes. This is consistent with earlier reports that both *RFT1* and *Hd3a* are important for flowering time regulation in cultivated rice [36]. The E105K allele is still under functional constraint for some reason. Our model implies that *RFT1* can be expressed under ND conditions, and can regulate *Hd3a*. We have revealed that variation in two loci, *RFT1* regulatory (I) and coding (II) regions, is involved in the regulation of flowering. Most group II *RFT1* cultivars had the Nona Bokra–like regulatory region and the defective protein (E105K). Only two cultivars (II-8 type, RGS and Puluik Arang) had Nona Bokra–like regulatory regions, but no E105K substitution (Figure 7). The II-8 allele might be the ancestral allele of group II defective *RFT1*. Furthermore, the haplotype network indicated that the group IV allele was the original allele of group II and III *RFT1*. These data suggest that the loss-of-function event in the regulatory region occurred first, and that the G to A (E105K) substitution was the second mutation.

Could *RFT1* be an example of pseudogenization? The presence of two florigens in rice, encoded by *Hd3a* and *RFT1*, might generate functional redundancy. However, previous studies and our current studies have shown that *RFT1* is regulated by unique mechanisms [40,49] and promotes flowering in the absence of *Hd3a* function [36]. The *RFT1* pathway is important in the late-flowering varieties (e.g. *Hd1* functional cultivars) under LD conditions or in high-latitude areas (Figure 10). Thus, the mechanisms may have been established by the gain of function after gene duplication. In general, *indica* rice is grown in tropical and subtropical regions at low latitudes, i.e., in warmer climate and shorter days, and these factors accelerate flowering. Under SD conditions, flowering time is mainly regulated by the *Hd3a* pathway (Figure 10), because *Hd3a* expression is induced earlier than *RFT1*. This situation may lead to a relaxation of functional constraint on *RFT1*. However, sometimes a mutation in a florigen protein leads to an opposite function: for example, paralogous genes *TFL1* and *FT* are key controllers of flowering but have opposite effects in *Arabidopsis* [38]. It was reported that only a single amino acid change is sufficient to switch the flowering promoter or repressor function of TFL1 and FT [72,73]. Therefore, limited mutations may be permissible in florigen genes.

We reconfirmed in a large scale test that *RFT1* and *Hd3a* diversity in *O. sativa* is lower than in *O* *. rufipogon* (Table 1) [50]. The number of haplotypes for the coding region of *RFT1* was lower than that of *Hd3a* in both species, and the same loss-of-function *RFT1* allele was found in both *indica* and *O* *. rufipogon* (Table 1, Figure S7). If the loss of function occurred in the common ancestor of *indica* and *rufipogon*, some mutations would have accumulated in the *RFT1* allele. However, the sequence of the defective *RFT1* allele of *O. sativaindica* (group II-1) is identical to that of *O* *. rufipogon* (W1723). This implies that the defective *RFT1* in W1723 was introgressed from an *indica* cultivar.

Perennial wild rice typically shows extremely late flowering; after germination, it may take two years or more to reach flowering even under SD conditions (at low latitudes). Vegetative growth and reproduction is the main propagation strategy of the wild rice species. Wild rice has strong photoperiod sensitivity, and some QTLs were reported around the *RFT1/Hd3a* region in perennial wild rice [50,54,74]. The nonsynonymous substitutions and a frameshift mutation we found in the *RFT1* and *Hd3a* coding regions in several wild *Oryza* species may underlie the variability of flowering time. Further studies are required to elucidate the role of *RFT1* and *Hd3a* in *Oryza* wild species and cultivars.

### Flowering time control for rice breeding

In this study, we found four major haplotypes of *RFT1*. The relationship between flowering time and *RFT1* haplotype suggested that group I, III and IV *RFT1* are functional alleles, whereas group II *RFT1* is nonfunctional (Table S3). No flowering time QTLs were previously detected around *RFT1* in crosses between Koshihikari (allele group I) and the following cultivars: Nipponbare (group I) [17], JAR (III) (Figure S8), NAB (IV) [51] or KASA (IV) [69] (Figure 7), consistent with group I, III and IV *RFT1* being functional haplotypes. Thus, these haplotype data would be useful for flowering time prediction. Group II *RFT1* haplotypes were found in the whole of Asia, whereas at lower latitudes they were found only in the presence of functional *Hd1* (Figure 9). This distribution was consistent with flowering responses to daylength (Figure 8). Furthermore, the varieties carrying group II and IV *RFT1* have mainly group I and IV *Hd1* alleles, respectively (Table S3) [24]. The *indica* subspecies contains two genetically distinct subgroups, *indica* and *aus*. Because independent events of domestication of *O* *. rufipogon* carrying the ancestral types of *Hd1* alleles I and IV of *indica* and *aus* group have occurred, respectively [24]. This suggests that the defective mutation occurred in the *indica* lineage after the divergence of *indica/aus*. *Indica* rice is grown mainly in tropical and subtropical regions. Therefore, the wide distribution of group II *RFT1* haplotypes might indicate that the defective *RFT1* allele first appeared at lower latitudes, and subsequently expanded to northern areas due to loss of function of flowering repressor gene(s) (e.g. *Hd1*) and artificial breeding.

We sequenced *RFT1 and Hd3a* in 204 accessions, including parental lines for rice breeding. We found some elite cultivars and accessions that have the defective *RFT1* allele (Table S3), for example IR36 (a “Green Revolution” rice cultivar from the International Rice Research Institute [IRRI]) [75] (Table S3). Although IR36 is an early-flowering cultivar, in breeding programs some of its progeny showed late flowering [76]. One of the parents of IR36, cv. TKM6, has defective *RFT1* (E105K) and is photoperiod-insensitive, but its progenies show various (and even high) photoperiod sensitivity [77]. The defective *RFT1* may be an unfavorable allele for breeding programs in middle- to high-latitude areas. On the other hand, we detected the defective *RFT1* in deep-water cultivars (Table S3). All deep-water rice accessions are photoperiod-sensitive [78]; flowering time is critical for propagation of their seeds after seasonal flooding. Our data suggested that *Hd3a* pathway acts as a single flowering activator in deep-water rice. To continue vegetative growth in water during the rainy season (in summer), *Hd3a* has to be repressed. According to our model (Figure 10), *Hd3a* is repressed by flowering time genes under LD conditions (in summer), but is induced after being subjected to SD conditions (in autumn), except in equatorial areas. Therefore, under particular environmental conditions (e.g. seasonal rapid flooding), defective *RFT1* might be a favorable allele.

The effect of defective *RFT1* on flowering is strong in functional *Hd1* and other flowering repressor gene backgrounds (e.g. *Hd16*) (Figure 2, S2). The tropical and subtropical *indica* cultivars often have not only defective *RFT1*, but also strong flowering repressor genes (e.g. *Hd1, Hd2* and *Hd6*) [50-54]. Therefore, when parents with defective *RFT1* are used in middle- or high-latitude areas, removing the defective *RFT1* region is advantageous. The ability to select for or against defective *RFT1* should make it easier to breed for cultivars with the desired flowering time. Our experimental evidence suggests that *RFT1* is the primary gene responsible for flowering time variation (see also 55). Most SNPs in the *RFT1* region showed strong linkage disequilibrium with the flowering-associated SNP [chr6_2912415] (Figure 7, S11), although the *r* ^2^ value for *RFT1* FNP(E105K) was relatively low (0.38). This may be explained by allele frequency differences and by the likely role of the *RFT1* regulatory regions (as opposed to the E105K substitution) as the primary determinant of late flowering under ND conditions. Therefore, it is possible to remove or select a defective *RFT1* haplotype by using group II *RFT1pro*-specific SNPs and FNP(E105K).

## Materials and Methods

### Plant materials and growth conditions

We crossed Koshihikari and SL520 [56], and used the progeny from self-pollinated individuals (Figure S1) for high-resolution mapping of the *RFT1* and *Hd3a* regions. Only Nona Bokra–type *Hd16* lines were selected for mapping in F_3_ (Figure S1). Within the F_3_ population, three regions were mapped around the *RFT1* and *Hd3a* regions in a paddy field in Tsukuba, Japan: 36°03′N, 140°10′E (Figure 1). The progeny from self pollination of these plants (F_4_) was used for the flowering time test and for the expression analysis under LD conditions. To verify the interaction between *RFT1* and *Hd1*, we crossed Koshihikari and KantoIL1, which contains the *Hd1* chromosomal region (*Hd1* nonfunctional allele) from the Kasalath genome [79] (Figure S1). We measured the flowering time in the self-pollinated progeny of these plants (F_2_) in a functional *Hd16* background. The molecular markers used for high-resolution mapping are listed in Tables S1 and S7. Genomic regions I and II, which include the *RFT1* promoter, coding region and recombination region are shown in Figure 1D. For fine mapping and flowering time evaluation under ND conditions, plants were grown from the middle of April. The daylengths during growth are shown in Figure 5B. For flowering evaluation and gene expression analysis, plants were grown in a growth cabinet under SD (10 h light/14 h dark) or LD (14.5 h light/ 9.5 h dark) conditions. Days to flowering under each condition were scored as the number of days required from germination to the emergence of the first panicle.

### *RFT1* cloning and generation of transgenic plants for complementation test

The Koshihikari *RFT1* genomic region (4,914 bp; Figure S4) was amplified by PCR and cloned into the *Sal*I site of pCR-Blunt II-TOPO (Invitrogen), resulting in TOPO-*RFT1*. TOPO-*RFT1* was digested with *Apa*I/*Kpn*I and *Kpn*I/*Xho*I, and subcloned into pPZP-2Hlac, digested with *Apa*I and *Xho*I [80], yielding pPZP-*gRFT1*. This plasmid was introduced into *Agrobacterium* strain EHA101 and subsequently into SL520, progeny from crossing between Koshihikari and SL520, and Nona Bokra by using the *Agrobacterium* -mediated rapid method [81].

### *RFT1* overexpression

Fragments containing the 5′-UTR of *RFT1* from Nipponbare (*nipRFT1*), Nona Bokra (*nbRFT1*) and Kasalath (*KasaRFT1*) were amplified by PCR with primers designed for Nipponbare cDNA (Table S7), and cloned into pCR-Blunt II-TOPO. The E105K mutation was introduced by PCR with RFT1-F/RFT1(E105K)-R and RFT1(E105K)-F/RFT1-R primer sets (Table S7) containing appropriate restriction sites, and the fragment obtained (*RFT1[E105K*]) was inserted into pPZP-Ha3(+) [80], resulting in pPZP-*35S*:*RFT1*. These plasmids were introduced into Nipponbare as mentioned above [81]. Regenerating transformed plants (T_0_) were incubated on Murashige and Skoog medium to induce roots under LL conditions for 20 days, and then transplanted to soil under LD conditions. We defined the flowering time (for each individual plant) as the time from transplanting onto the regeneration medium to the appearance of the first panicle.

### Expression analysis

Nipponbare (reference genome cultivar), Koshihikari and Nona Bokra were used for expression analysis under ND conditions. They were cultivated from April to October. Nona Bokra was transplanted to a greenhouse from October to avoid low temperatures. Leaf samples were collected every 7 days from 37 to 120 days after sowing, and every 14 days from 135 to 218 days (at 9:00 am).

The control line, #3098-2-1-1 and Nona Bokra were used for expression analysis under LD conditions. Leaf samples were collected every 10 days from 10 to 90 days after sowing. Additionally, Nona Bokra samples were collected 120 and 180 days after sowing.

Plants overexpressing *nipRFT1*, *nbRFT1*, *KasaRFT1* and *RFT1*(*E105K*) were used for expression analysis under LL conditions (20 days after transplanting onto regeneration medium).

Total RNA extraction and TaqMan quantitative real-time PCR (qRT-PCR) analysis were performed as described previously [14]. Expression data were normalized against *UBQ* expression.

### Sequencing of *RFT1* and *Hd3a* in *O. sativa* accessions and wild rice species

The upstream regions and the ORFs of *RFT1* and *Hd3a* were amplified by PCR (94°C for 30 s, 60°C for 60 s, and 72°C for 60 s; 35 cycles) with primers listed in Table S7 and AmpliTaq DNA (Applied Biosystems). The nucleotide sequences of the PCR products were analyzed with an ABI3700 capillary sequencer (Applied Biosystems). Accessions were provided by NIAS Gene Bank [60], National BioResource Project (NBRP) [82] and IRRI [http://singer.cgiar.org/]. For the wild *Oryza* species (except for *O* *. rufipogon* ), BAC clones were selected. The BAC resources of *O* *. glaberrima* *,* *O. glumaepatula* *,* *O. barthii* *,* *O* *. meridionalis* *and O. longistaminata* were supplied by the Rice Genome Research Program, and those of *O* *. punctata* *,* *O* *. minuta* *,* *O* *. officinalis* *,* *O* *. alta* *,* *O* *. australiensis* *,* *O* *. brachyantha* and *O* *. granulata* were supplied by the Arizona Genomics Institute [http://www.genome.arizona.edu/].

### Sequence analysis

Multiple alignment and evolutionary analyses of the FT-like proteins were conducted in MEGA5 [83]. To consider the codon structure, multiple alignment of 363 sequences was performed in MUSCLE [84]. For further analyses, positions with gaps were excluded. To analyze the equality of evolutionary rates of *RFT1* and *Hd3a* in each accession, Tajima’s relative rate test was performed with orthologs from either *B* *. distachyon* (Bradi1g48830) or *S. bicolor* (Sb10g003940) as an outgroup [63-65]. The numbers of nonsynonymous substitutions per nonsynonymous site and of synonymous substitutions per synonymous site were calculated by using the modified Nei–Gojobori model (assumed transition/transversion bias = 2) [86].

### Accession Codes

Os06g0157500/LOC_Os06g06300.1 (*RFT1/OsFTL3*), Os06g0157700/LOC_Os06g06320.1 (*Hd3a/OsFTL2*), Os01g0218500/LOC_Os01g11940.1 (*FTL/OsFTL1*), Os06g0298200 / LOC_Os06g19444 (*Hd1*), Os10g0463400 / LOC_Os10g32600.1 (*Ehd1*), Os04g0488400/LOC_Os04g41130.1 (*OsFTL6*), Os01g0202700/LOC_Os01g10590.1 (*OsFTL8*), Os12g0232300/LOC_Os12g13030.1 (*OsFTL7*), Os05g0518000/LOC_Os05g44180.1 (*OsFTL10*), Os11g0293800/LOC_Os11g18870.1 (*OsFTL11*), Os06g0552900/LOC_Os06g35940.1 (*OsFTL12*), Os02g0232300/LOC_Os02g13830.1 (*OsFTL13*), Os02g0602601 / LOC_Os02g39064.1 (*OsFTL5*), Os09g0513500 / LOC_Os09g33850.1 (*OsFTL4*) and Os01g074880 / LOC_Os01g54490.1(*OsFTL9*) in RAP-DB (http://rapdb.affrc.go.jp/) [85] / MSU (http://rice.plantbiology.msu.edu/index.shtml) [86]. Accession codes used in Figure S9 are underlined.

The accession numbers for the genes used in overexpression analysis are Nipponbare (*nipRFT1ox*, AB062675), Kasalath (*kasaRFT1ox*, AB426870) and Nona Bokra (*nbRFT1ox*, AB809564). The accession numbers for sequences of *RFT1* and *Hd3a* from rice accessions can be found in GenBank (AB564440-AB564463, AB838243-AB838594).

## Supporting Information

Figure S1 **Development of the plant materials used for fine mapping and studies on interaction between flowering time genes.** Graphical genotypes of KantoIL1 [79], SL520 [56], Koshihikari, Nona Bokra and lines developed in this study are shown. KantoIL1 has nonfunctional Hd1 from Kasalath [79].
(TIF)

Figure S2 **Frequency distribution of flowering time of F_2_ plants derived from a cross between Koshihikari and SL520 under ND conditions (A).** White, gray and black boxes show the Koshihikari, heterozygous and Nona Bokra *RFT1*/*Hd3a* genotypes, respectively. (B) Graphical genotype of SL520. The numbered bars represent the 12 chromosomes of rice. Black lines indicate SSR markers. Black and gray boxes represent Nona Bokra and heterozygous segments. (C, D) Flowering time of F_2_ plants derived from a cross between Koshihikari (Kos) and SL520 under ND (C) and LD (D) conditions. (C) is based on (A). *RFT1* and *Hd16* genotypes are shown: N in black boxes, Nona Bokra; K in white boxes, Koshihikari; H in gray boxes, heterozygous. Each bar represents the mean ± SD.
(TIF)

Figure S3 **Delimitation of the candidate *RFT1/Hd3a* genomic region and genetic effects of recombination on flowering under LD conditions.** Left: Graphical genotypes of the *RFT1* and *Hd3a* region in parental lines and in control and four recombinant lines in which recombination occurred between InDel1 and RM7488. Right: The days to flowering of control and recombinant plants (F_4_ generation [Figure S1]) under LD conditions. Kos, Koshihikari; Con, control line (Figure S1).
(TIF)

Figure S4 ***RFT1* nucleotide sequence in Koshihikari.** Nucleotide sequence of *RFT1* genomic region for complementation test in Koshihikari is shown. The physical position (from 2,922,570 to 2,927,483 on Chr. 6) and the sequence correspond to Nipponbare reference genome (RAP-DB build 5.0). The 4,914-bp region shown was used for the complementation test (Figure 3). Characters above the sequence represent polymorphic sites in Nona Bokra; black and white triangles, insertions and deletions in Nona Bokra, respectively; amino acid substitutions in Nona Bokra are shown below the nucleotide sequence (original > changed); blue and red characters indicate the CAAT boxes and the ARR1 binding elements, respectively; SNP and InDel markers are shown as aqua balloons; the purple box indicates the recombination region between the two markers in 3098#1 (Figure 1C); the coding region is shaded in yellow; 5′- and 3′-UTR regions are shown in gray. Black half-arrows indicate primer sets for qRT-PCR (a/b [23] and c/d [this study]); the TaqMan probe used with c/d primers is shown as a red bar.
(TIF)

Figure S5 **Correlation of flowering time with the mRNA levels of *RFT1* and *Hd3a* under SD (A), LD (B) and ND (C) conditions.** The mRNA levels in the top leaves from plants 20 (A), 40 (B) and 64 (C) days old (five plants per point) were determined by qRT-PCR. The data are shown on a log_10_ scale. (D) Pearson correlation coefficients between flowering time and mRNA levels under SD, LD and ND conditions. ***, *P*<0.001; **, *P*<0.05; *, *P*<0.01.
(TIF)

Figure S6 **Phylogenic tree of the FT-like clade in rice (A) and mRNA levels of six FT-like genes during growing period under natural field conditions (B).** (A) The tree was constructed in MEGA5 [83]. (B) The data are from Rice-XPro [58]. The plants were grown in in paddy field in Tsukuba (Japan).
(TIF)

Figure S7 **Haplotype network of the *RFT1* and *Hd3a* coding region.** . The haplotypes are represented by colored circles; their size is proportional to the number of individuals showing that haplotype. Haplotype network generated on the basis of the maximum-likelihood tree by Network 4.611 [2].
(TIF)

Figure S8 **QTLs for flowering time.** (A) Frequency distribution of flowering time in the 89 F_2_ population derived from a cross between Koshihikari and Jarjan. (B) Linkage map of the F_2_ population. Crossed lines show the positions of 164 RM markers used for the whole genome survey. (C) LOD score plot based on composite interval mapping (R/qtl) [3]. Black triangles indicate the position of *Hd16, Hd6, RFT1* and *Hd1*. (D) Effect plots depicting the effects of *RFT1* allele on flowering time in *Hd16* (Jarjan allele) and *Hd1* (Koshihikari allele) functional background.
(PDF)

Figure S9 **Nucleotide polymorphisms in the coding regions of *RFT1* and *Hd3a* (185 and 183 accessions, respectively).** *RFT1* and *Hd3a* coding regions of plants from the 204 rice accessions and wild rice species were compared with those of Nipponbare (WRC001). Physical positions are based on the Nipponbare coding sequence (RAP-DB build 5.0 [85]). Nucleotide substitutions are highlighted in orange, green, yellow or pink. Gray and purple boxes represent UTRs and ORF of *RFT1* and *Hd3a*, respectively. Amino acid changes are indicated above the alignment. The number of cultivars with each type of sequence is shown in the column at the right.
(TIF)

Figure S10 **Scatter plot of dN/dS ratios of orthologous gene of *Brachypodium* (*BradHd3a*) and *Sorghum* (*SbHd3a*) versus *O. sativa RFT1 and Hd3a*** (**144 accessions; see** Table S3, S4).
(TIF)

Figure S11 **Gene structure and linkage disequilibrium in *RFT1* and *Hd3a*** Pair-wise measures of LD (*D'* and *r* ^*2*^) were calculated using Haploview [1]. The position of the flowering time associated SNP [55] and SNPs in *RFT1* and *Hd3a* genomic region are shown the upper part of the figure. The triangular part of the figure shows the linkage disequilibrium (LD) pattern as measured by *D'* and *r* ^*2*^ between the SNPs. Red squares indicate high pairwise LD, gradually coloring down to white squares of low pairwise LD. *r* ^2^ values x100 are indicated within squares. The position of flowering time associated SNP, RFT1(E105K)-FNP SNP in this study and Hd3a(P179N)-candidate FNP SNP are denoted by red line. The *r* ^2^ values between flowering time associated SNP and FNP SNPs are in blue boxes. The names and positions for all SNPs used are given in Table S6.
(TIF)

Table S1 **SSR markers and genotype of SL520.** The “Locus name” column shows SSR markers. “p” indicates small band size differences, “P” indicates clear differences (good markers). K and N, Koshihikari and Nona Bokra genotypes, respectively. BLAST-chr indicates the chromosome. The fifth column gives the genetic distance. Physical positions are shown in sixth and seventh column as (BLAST position start and end) (RAP-DB build 3.0).
(PDF)

Table S2 **Information for the 24 cultivars used in** Figure S5.
(PDF)

Table S3 **Information for 204 tested rice accessions.**
(PDF)

Table S4 **Tajima’s relative rate test for *Hd3a* and *RFT1* using *BradHd3a* ( *Brachypodium* *distachyon* ) DNA sequence as an out group.**
(PDF)

Table S5 **Tajima’s relative rate test for *Hd3a* and *RFT1* using *SbHd3a* ( *Sorghum* *bicolor* ) DNA sequence as an out group.**
(PDF)

Table S6 **SNP information for Haploview plot.**
(PDF)

Table S7 **Primer sequences of newly designed DNA markers used for linkage mapping.** Amplified fragment size differences are shown in the “Name” column. Target SNPs and restriction enzymes used to detect polymorphism are shown for CAPS markers. SNP-1, 2 and 3 are SNP marker. These target SNPs are shown as red character in Note/SNP column. These SNPs were detected by using Acyclo-Prime FP detection kit (PerkinElmer Life Science). Primers for expression analysis were previously described [14,19].
(PDF)

File S1 **Supplementary References.**
(DOCX)
