# Supplementary figures and images for: Natural Variation of the RICE FLOWERING LOCUS T 1 Contributes to Flowering Time Divergence in Rice
Source: PLoS One. 2013 Oct 1;8(10):e75959. doi: 10.1371/journal.pone.0075959 (PMC3788028; doi:10.1371/journal.pone.0075959)

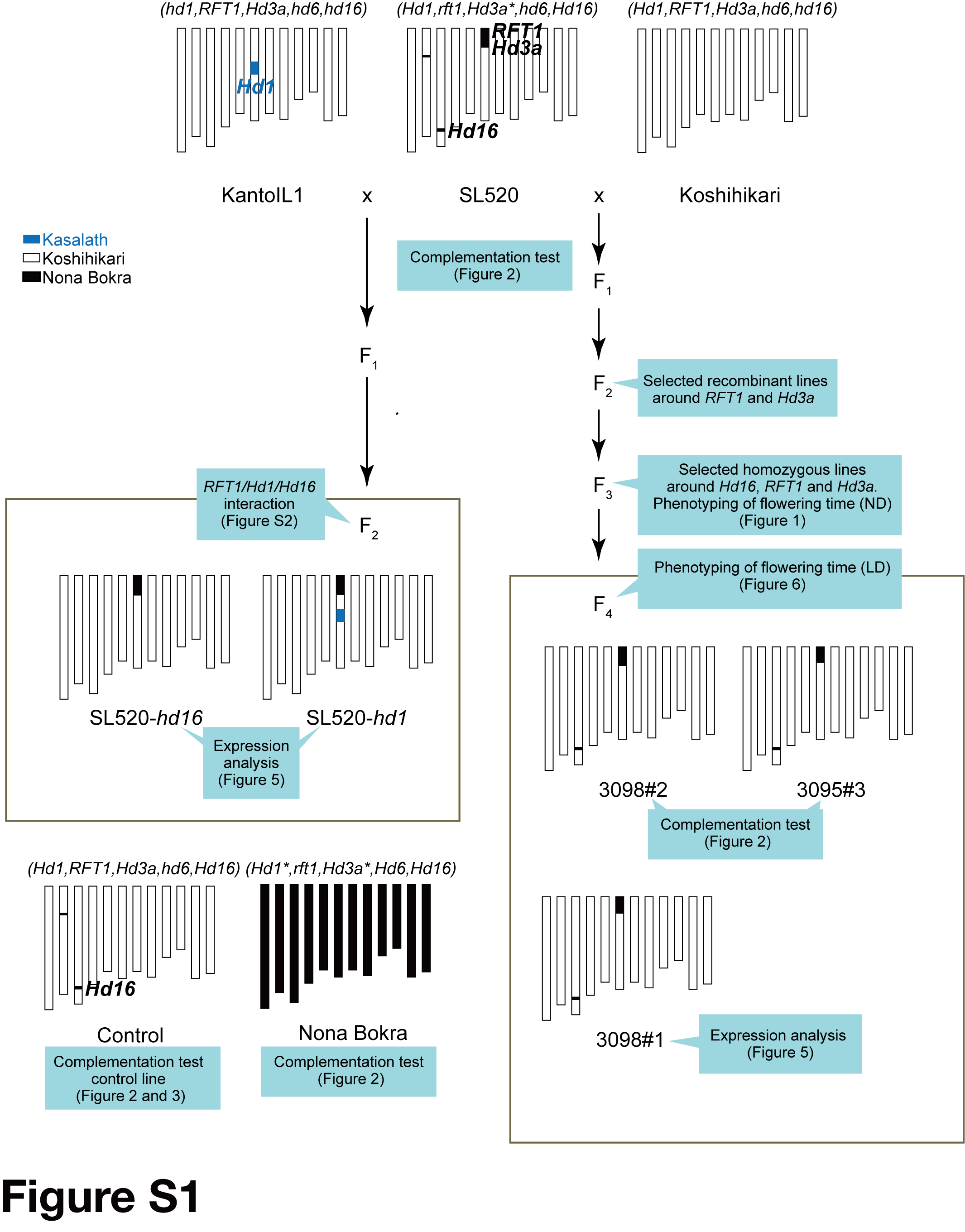

Supplement: Figure S1 — Development of the plant materials used for fine mapping and studies on interaction between flowering time genes. Graphical genotypes of KantoIL1 [79], SL520 [56], Koshihikari, Nona Bokra and lines developed in this study are shown. KantoIL1 has nonfunctional Hd1 from Kasalath [79]. (TIF) [file pone.0075959.s001.tif]

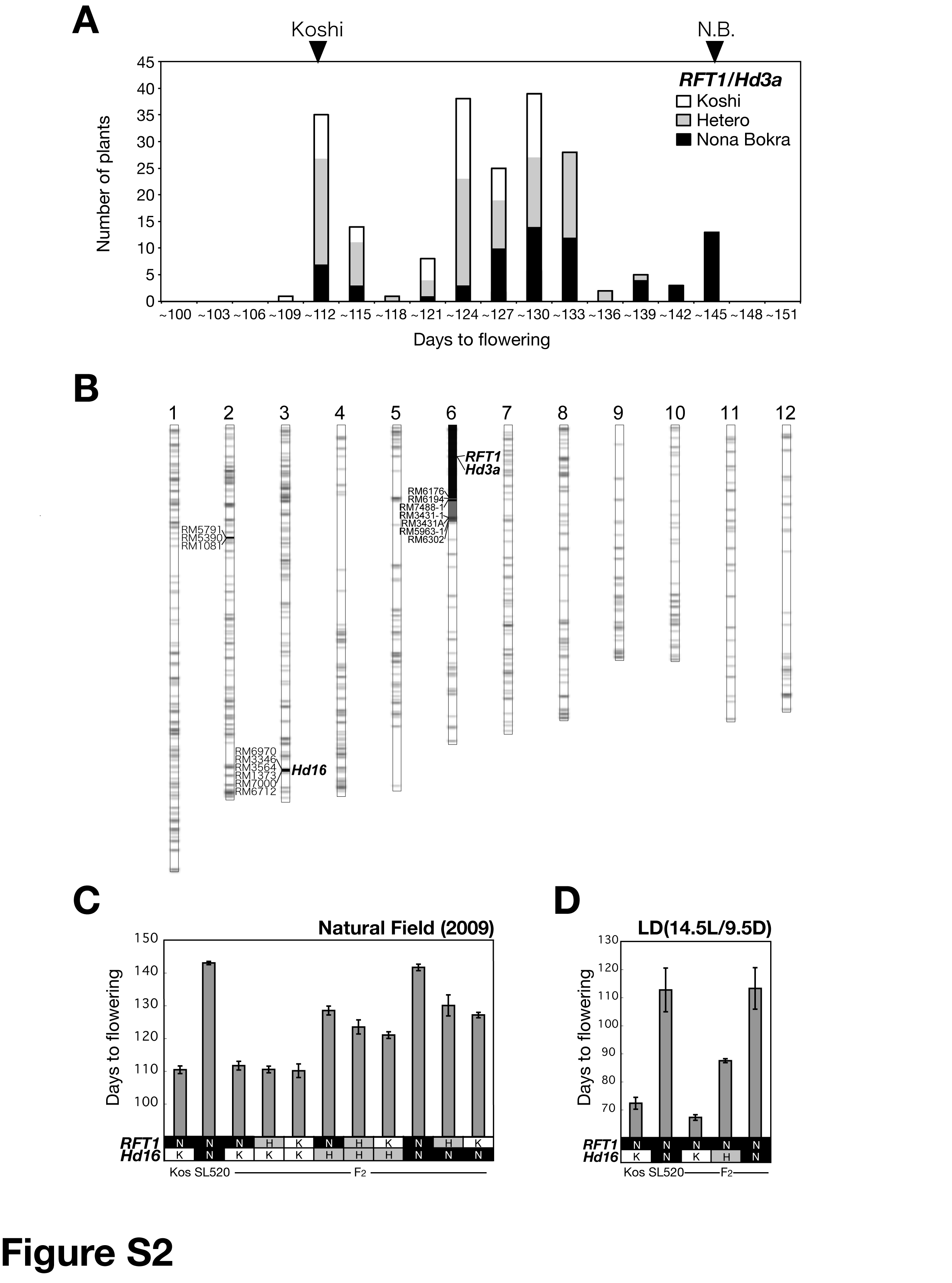

Supplement: Figure S2 — Frequency distribution of flowering time of F2 plants derived from a cross between Koshihikari and SL520 under ND conditions (A). White, gray and black boxes show the Koshihikari, heterozygous and Nona Bokra RFT1/Hd3a genotypes, respectively. (B) Graphical genotype of SL520. The numbered bars represent the 12 chromosomes of rice. Black lines indicate SSR markers. Black and gray boxes represent Nona Bokra and heterozygous segments. (C, D) Flowering time of F2 plants derived from a cross between Koshihikari (Kos) and SL520 under ND (C) and LD (D) conditions. (C) is based on (A). RFT1 and Hd16 genotypes are shown: N in black boxes, Nona Bokra; K in white boxes, Koshihikari; H in gray boxes, heterozygous. Each bar represents the mean ± SD. (TIF) [file pone.0075959.s002.tif]

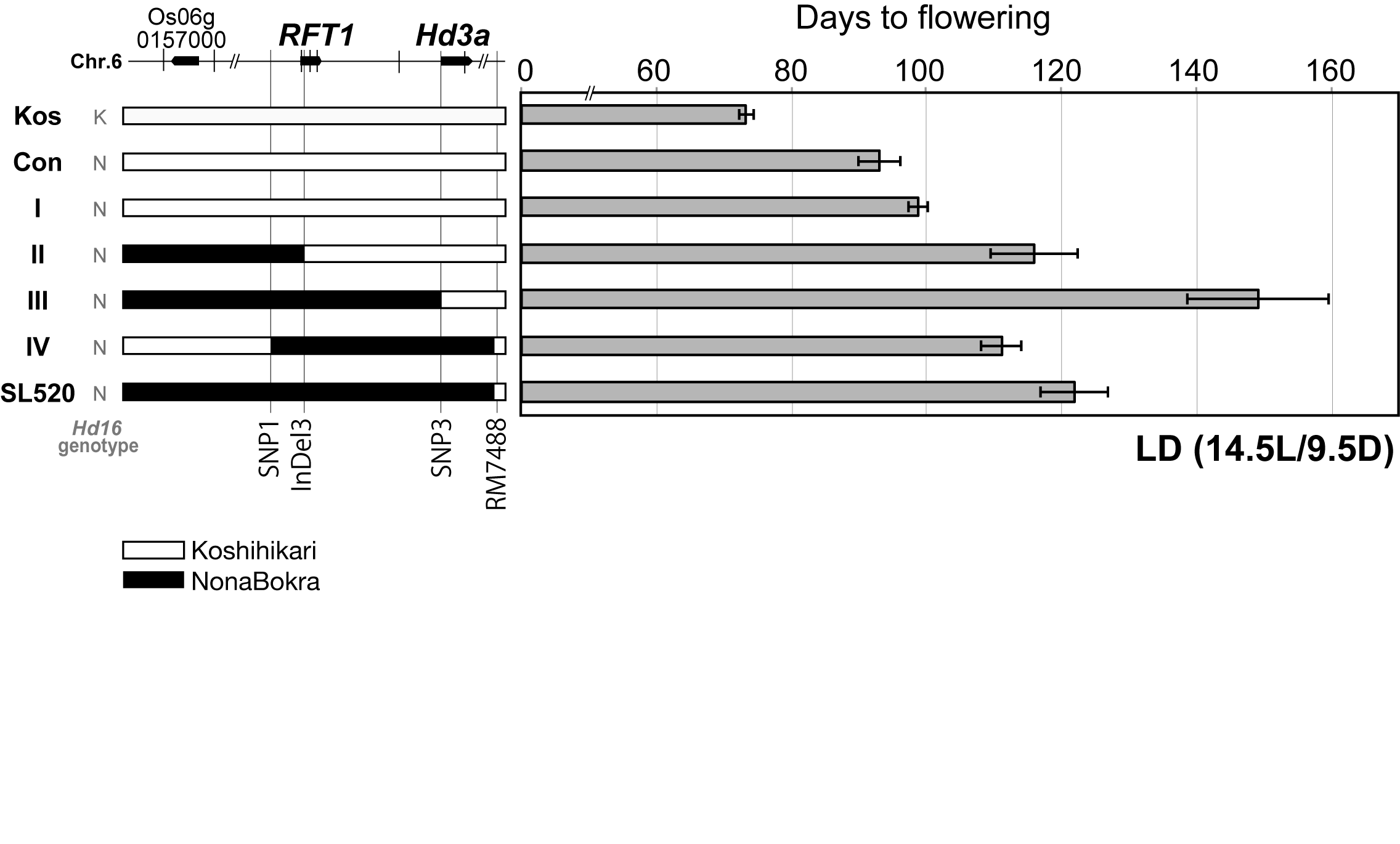

Supplement: Figure S3 — Delimitation of the candidate RFT1/Hd3a genomic region and genetic effects of recombination on flowering under LD conditions. Left: Graphical genotypes of the RFT1 and Hd3a region in parental lines and in control and four recombinant lines in which recombination occurred between InDel1 and RM7488. Right: The days to flowering of control and recombinant plants (F4 generation [Figure S1]) under LD conditions. Kos, Koshihikari; Con, control line (Figure S1). (TIF) [file pone.0075959.s003.tif]

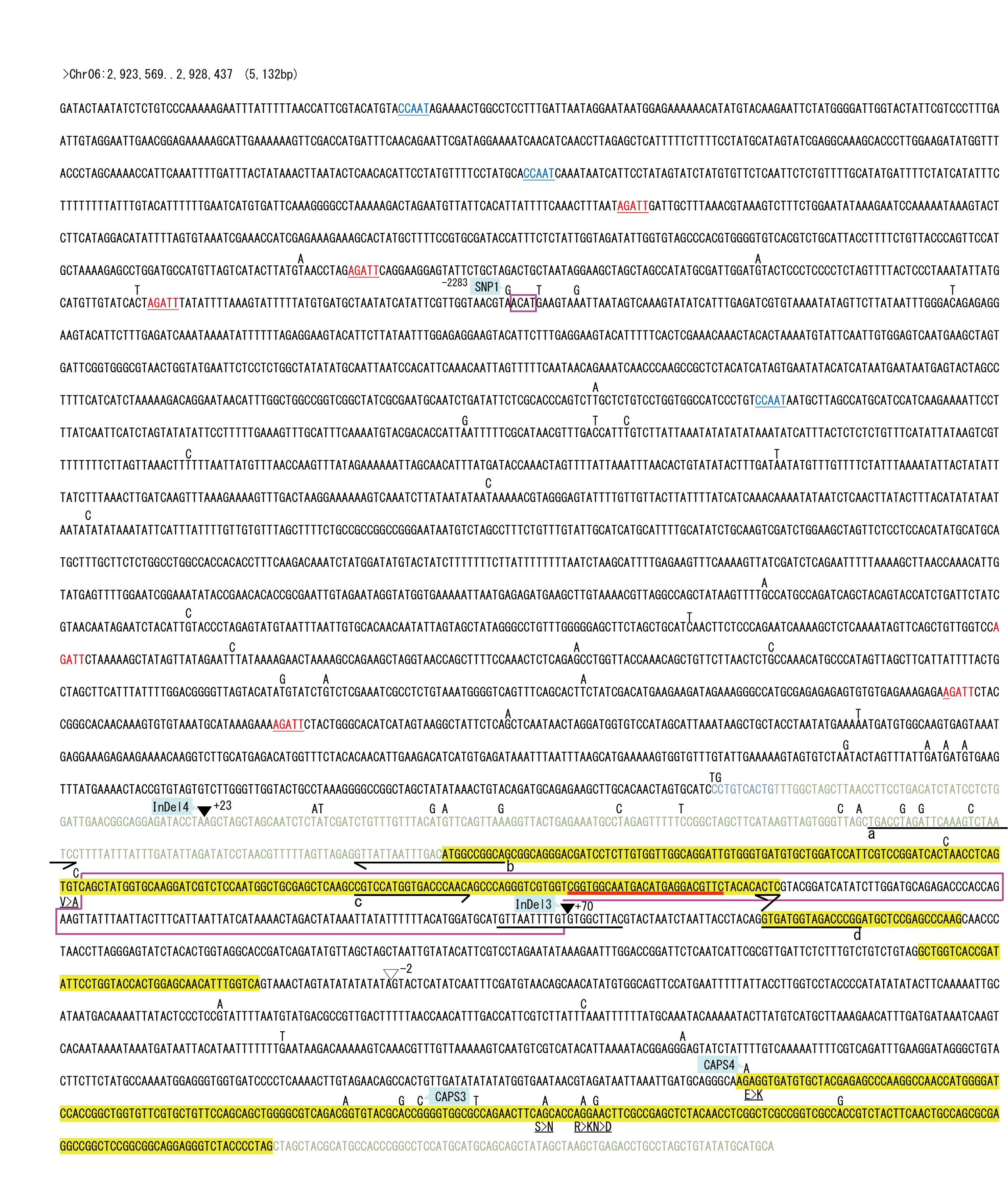

Supplement: Figure S4 — RFT1 nucleotide sequence in Koshihikari. Nucleotide sequence of RFT1 genomic region for complementation test in Koshihikari is shown. The physical position (from 2,922,570 to 2,927,483 on Chr. 6) and the sequence correspond to Nipponbare reference genome (RAP-DB build 5.0). The 4,914-bp region shown was used for the complementation test (Figure 3). Characters above the sequence represent polymorphic sites in Nona Bokra; black and white triangles, insertions and deletions in Nona Bokra, respectively; amino acid substitutions in Nona Bokra are shown below the nucleotide sequence (original > changed); blue and red characters indicate the CAAT boxes and the ARR1 binding elements, respectively; SNP and InDel markers are shown as aqua balloons; the purple box indicates the recombination region between the two markers in 3098#1 (Figure 1C); the coding region is shaded in yellow; 5′- and 3′-UTR regions are shown in gray. Black half-arrows indicate primer sets for qRT-PCR (a/b [23] and c/d [this study]); the TaqMan probe used with c/d primers is shown as a red bar. (TIF) [file pone.0075959.s004.tif]

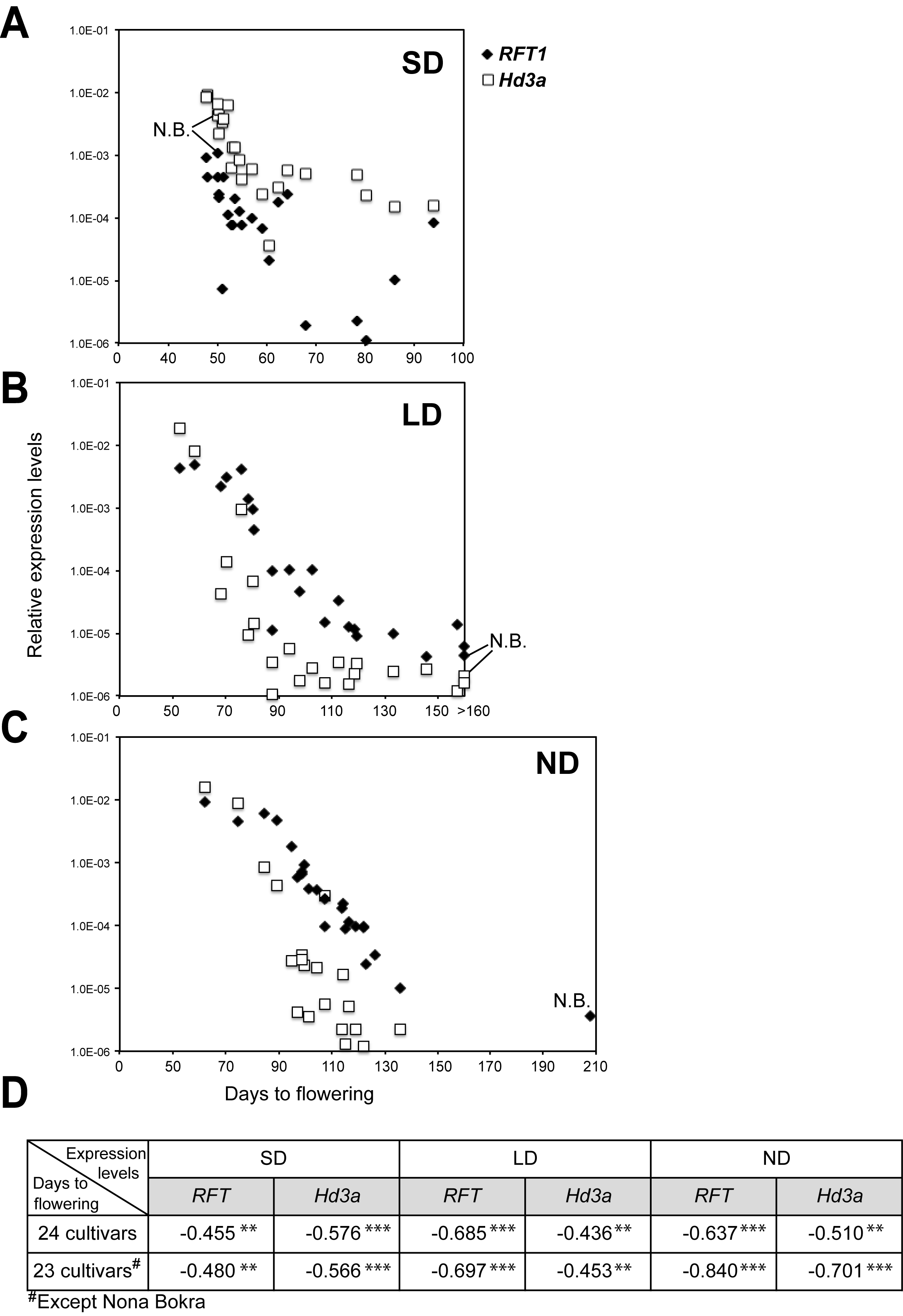

Supplement: Figure S5 — Correlation of flowering time with the mRNA levels of RFT1 and Hd3a under SD (A), LD (B) and ND (C) conditions. The mRNA levels in the top leaves from plants 20 (A), 40 (B) and 64 (C) days old (five plants per point) were determined by qRT-PCR. The data are shown on a log10 scale. (D) Pearson correlation coefficients between flowering time and mRNA levels under SD, LD and ND conditions. ***, P<0.001; **, P<0.05; *, P<0.01. (TIF) [file pone.0075959.s005.tif]

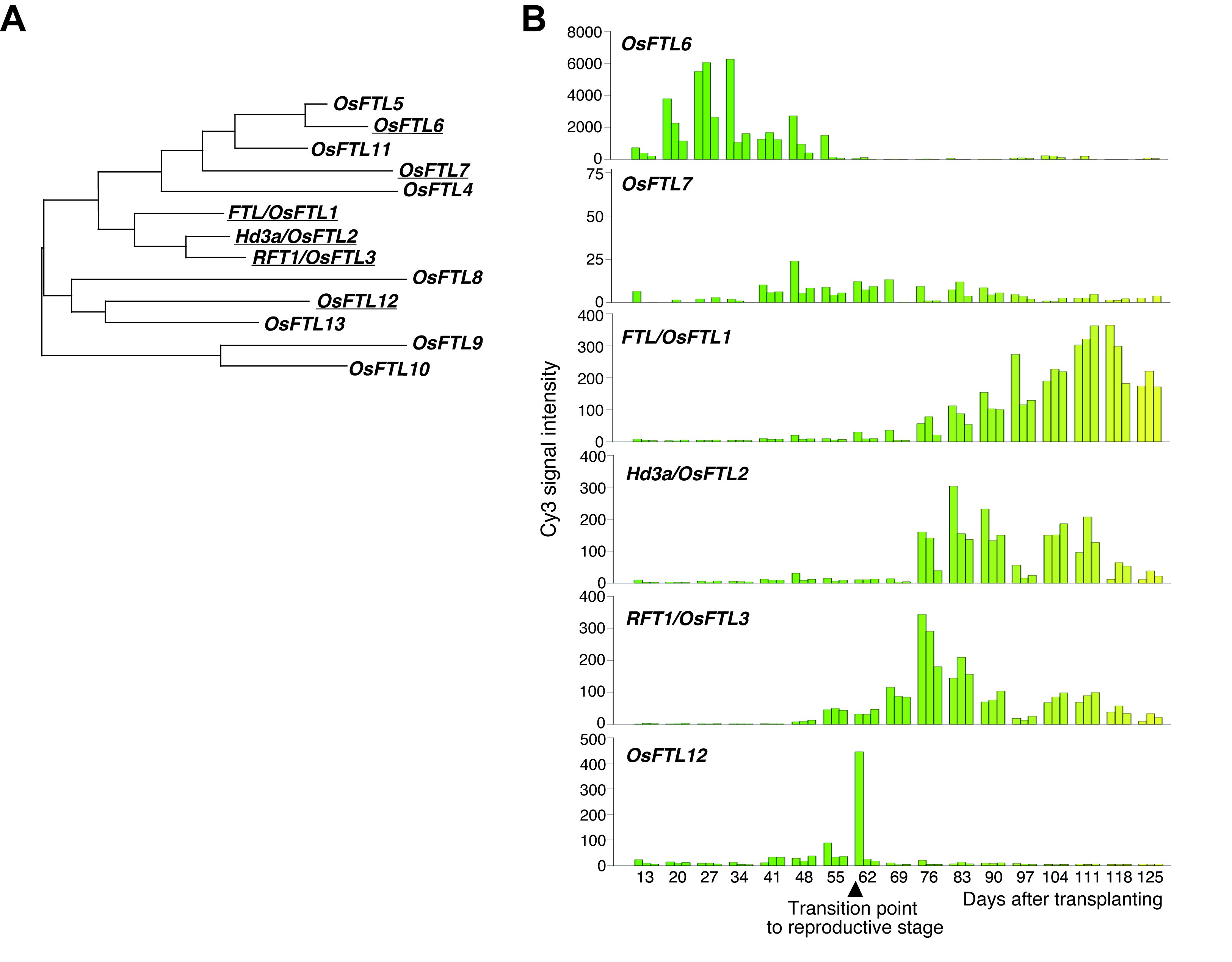

Supplement: Figure S6 — Phylogenic tree of the FT-like clade in rice (A) and mRNA levels of six FT-like genes during growing period under natural field conditions (B). (A) The tree was constructed in MEGA5 [83]. (B) The data are from Rice-XPro [58]. The plants were grown in in paddy field in Tsukuba (Japan). (TIF) [file pone.0075959.s006.tif]

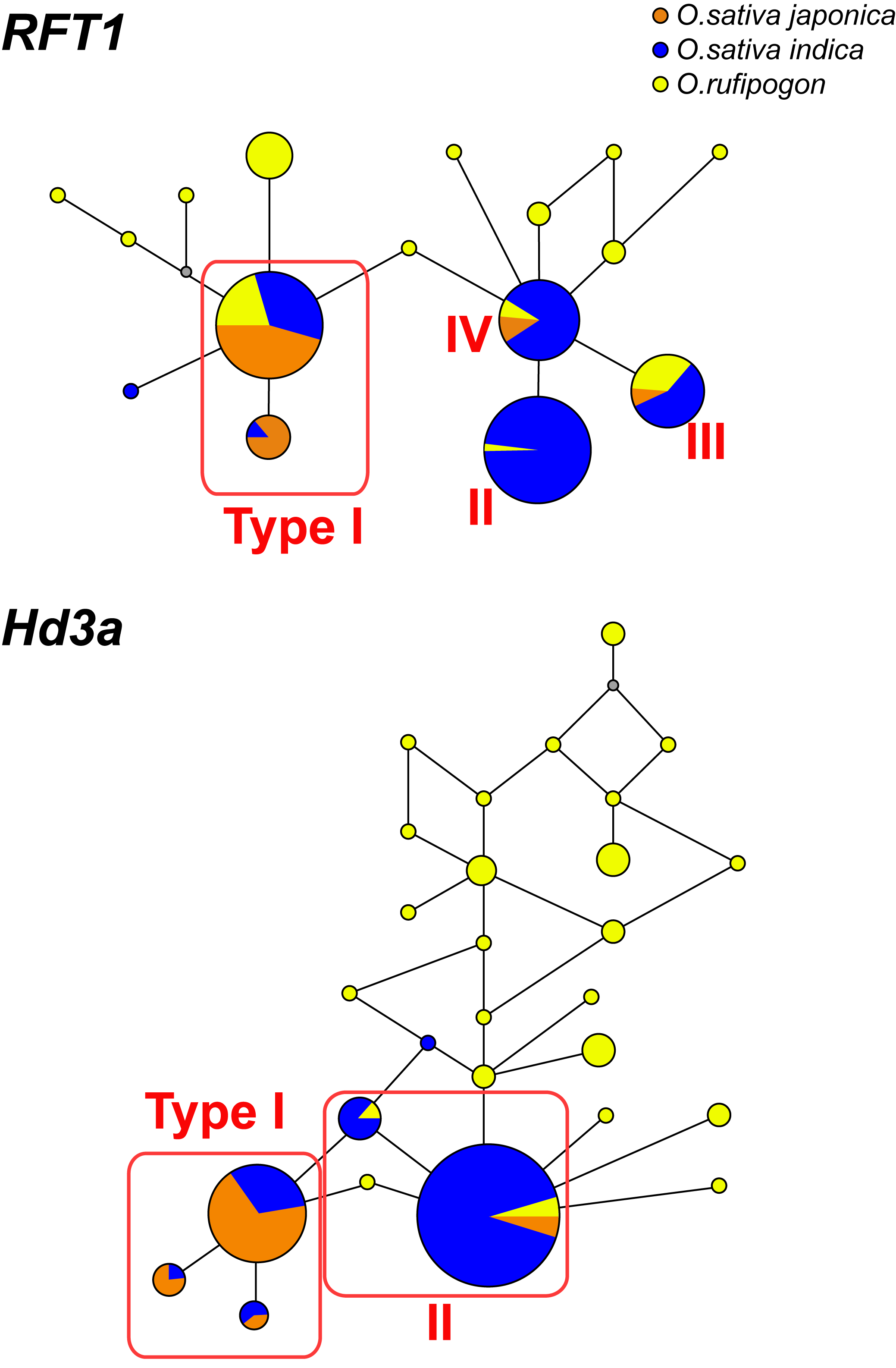

Supplement: Figure S7 — Haplotype network of the RFT1 and Hd3a coding region. . The haplotypes are represented by colored circles; their size is proportional to the number of individuals showing that haplotype. Haplotype network generated on the basis of the maximum-likelihood tree by Network 4.611 [2]. (TIF) [file pone.0075959.s007.tif]

**A**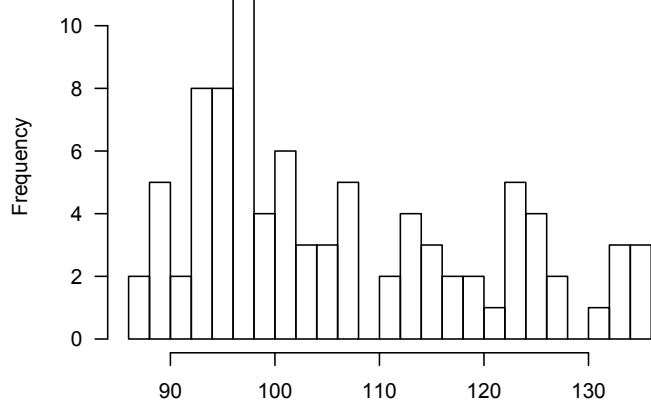**B**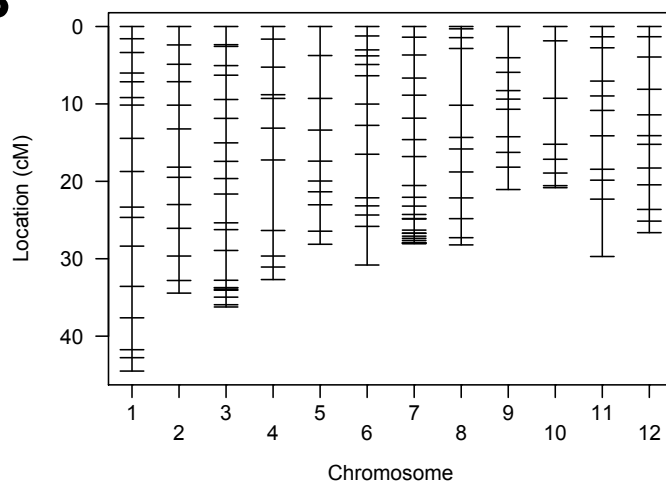**C**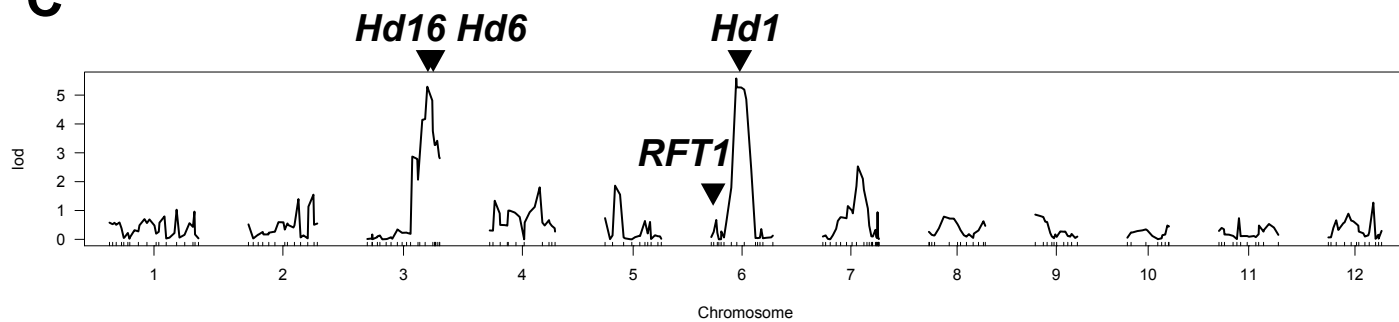

Supplement: Figure S8 — QTLs for flowering time. (A) Frequency distribution of flowering time in the 89 F2 population derived from a cross between Koshihikari and Jarjan. (B) Linkage map of the F2 population. Crossed lines show the positions of 164 RM markers used for the whole genome survey. (C) LOD score plot based on composite interval mapping (R/qtl) [3]. Black triangles indicate the position of Hd16, Hd6, RFT1 and Hd1. (D) Effect plots depicting the effects of RFT1 allele on flowering time in Hd16 (Jarjan allele) and Hd1 (Koshihikari allele) functional background. (PDF) [file pone.0075959.s008.pdf]

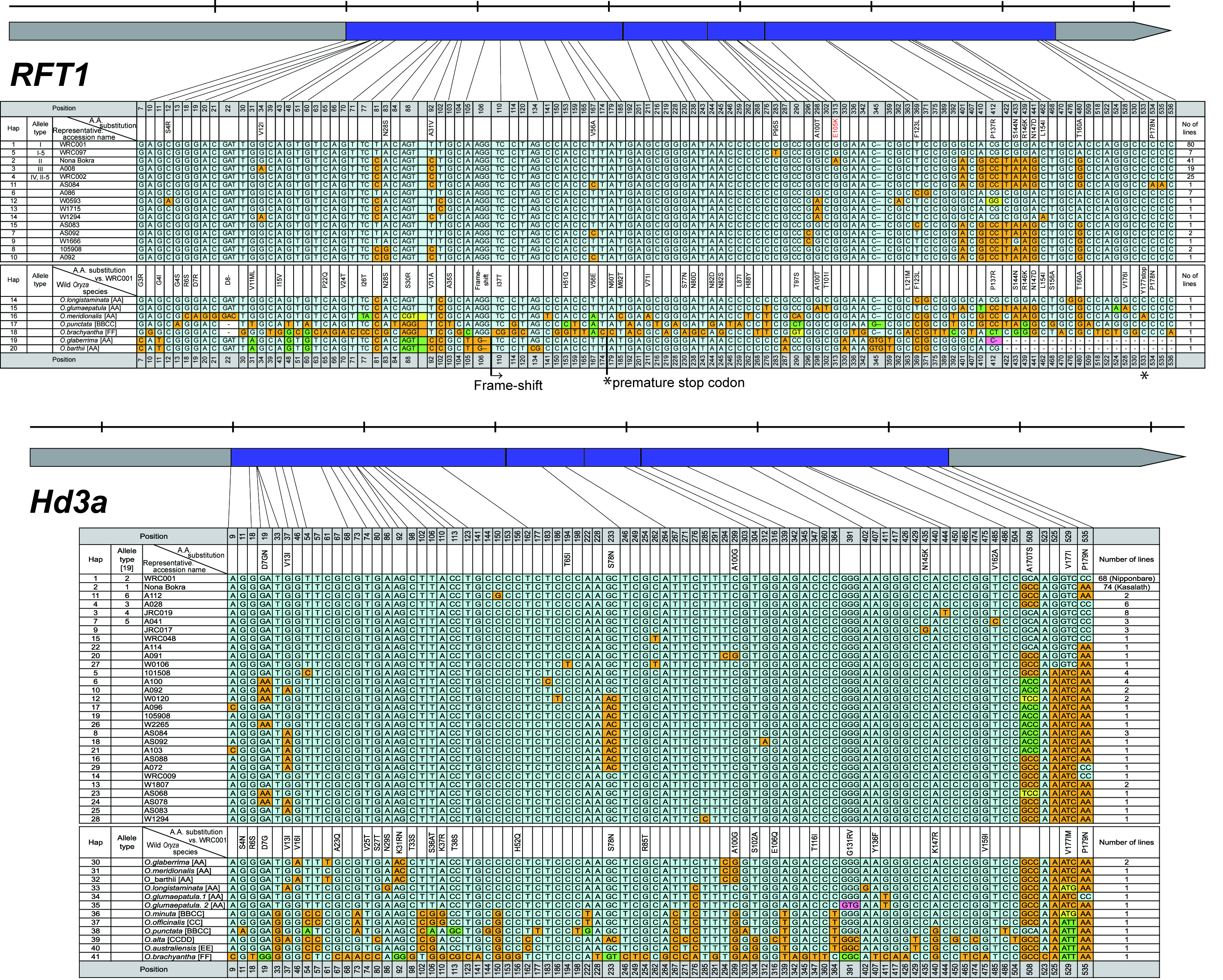

Supplement: Figure S9 — Nucleotide polymorphisms in the coding regions of RFT1 and Hd3a (185 and 183 accessions, respectively). RFT1 and Hd3a coding regions of plants from the 204 rice accessions and wild rice species were compared with those of Nipponbare (WRC001). Physical positions are based on the Nipponbare coding sequence (RAP-DB build 5.0 [85]). Nucleotide substitutions are highlighted in orange, green, yellow or pink. Gray and purple boxes represent UTRs and ORF of RFT1 and Hd3a, respectively. Amino acid changes are indicated above the alignment. The number of cultivars with each type of sequence is shown in the column at the right. (TIF) [file pone.0075959.s009.tif]

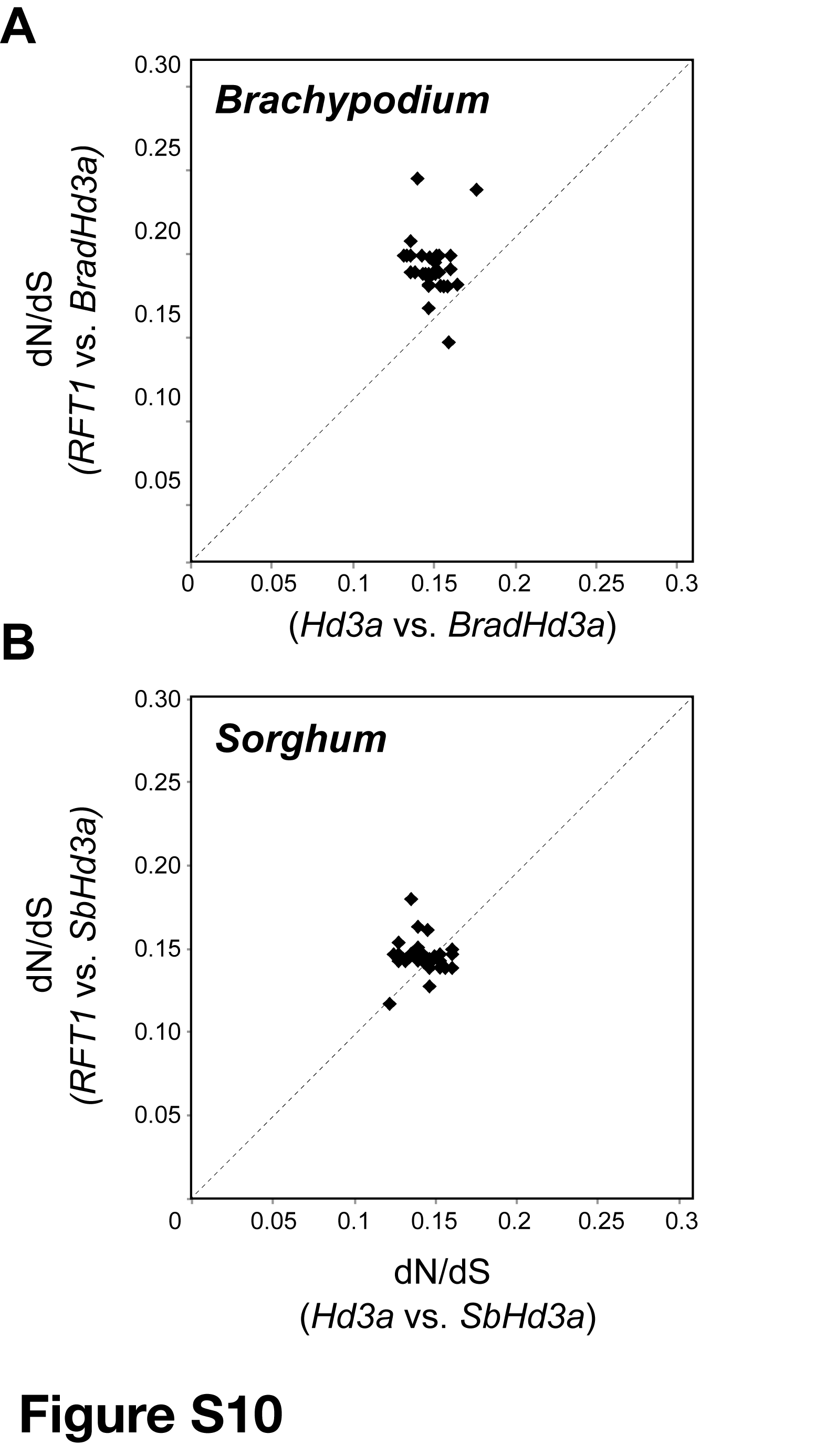

Supplement: Figure S10 — Scatter plot of dN/dS ratios of orthologous gene of Brachypodium (BradHd3a) and Sorghum (SbHd3a) versus O. sativa RFT1 and Hd3a (144 accessions; see Table S3, S4). (TIF) [file pone.0075959.s010.tif]

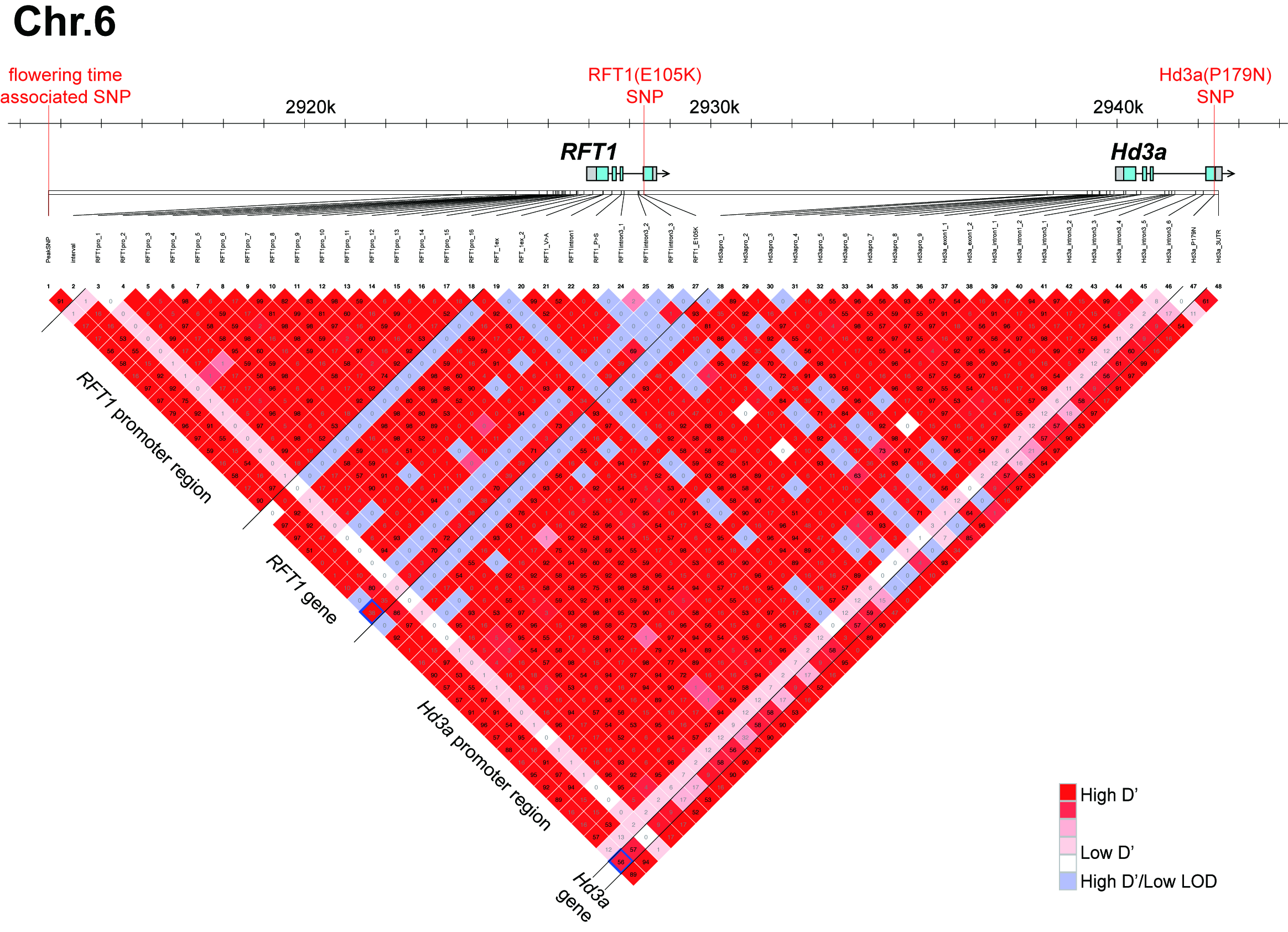

Supplement: Figure S11 — Gene structure and linkage disequilibrium in RFT1 and Hd3a Pair-wise measures of LD (D' and r 2) were calculated using Haploview [1]. The position of the flowering time associated SNP [55] and SNPs in RFT1 and Hd3a genomic region are shown the upper part of the figure. The triangular part of the figure shows the linkage disequilibrium (LD) pattern as measured by D' and r 2 between the SNPs. Red squares indicate high pairwise LD, gradually coloring down to white squares of low pairwise LD. r 2 values x100 are indicated within squares. The position of flowering time associated SNP, RFT1(E105K)-FNP SNP in this study and Hd3a(P179N)-candidate FNP SNP are denoted by red line. The r 2 values between flowering time associated SNP and FNP SNPs are in blue boxes. The names and positions for all SNPs used are given in Table S6. (TIF) [file pone.0075959.s011.tif]
