# Supplementary material for: Natural Variation of the RICE FLOWERING LOCUS T 1 Contributes to Flowering Time Divergence in Rice
Source: PLoS One. 2013 Oct 1;8(10):e75959. doi: 10.1371/journal.pone.0075959 (PMC3788028; doi:10.1371/journal.pone.0075959)
Supplement: Table S1 — SSR markers and genotype of SL520. The “Locus name” column shows SSR markers. “p” indicates small band size differences, “P” indicates clear differences (good markers). K and N, Koshihikari and Nona Bokra genotypes, respectively. BLAST-chr indicates the chromosome. The fifth column gives the genetic distance. Physical positions are shown in sixth and seventh column as (BLAST position start and end) (RAP-DB build 3.0). (PDF) [file pone.0075959.s012.pdf]

Table S1 Genotype of SL520

Marker Name	Koshihikari/NonaBokra	SL520 genotype	Position			
			Chromosome	Genetic distance (cM)	Physical start position*	Physical end position*
RM6887–1	p	K	1	0.3	207,759	207,986
RM6672	P	K	1	0.3–4.2	281,797	282,026
RM3252	P	K	1	0.3–4.2	301,847	302,072
RM4554	P	K	1	0.3–4.2	388,832	389,075
RM1282	P	K	1	4.2	548,571	548,804
RM6340	P	K	1	7	1,058,440	1,058,663
RM1331	p	K	1	8.7	1,669,100	1,669,339
RM8068	p	–	1	8.7		
RM8069	P	K	1	8.7		
RM6045	p	K	1	10.9	1,775,753	1,775,979
RM7278	P	K	1	10.9	1,793,825	1,794,064
RM5423	p	K	1	10.9	2,169,122	2,169,353
RM6324	P	K	1	11.7	2,376,751	2,376,998
RM3740	P	K	1	12.5–13.1	2,799,991	2,800,222
RM7383	p	K	1	16.4	3,483,382	3,483,609
RM3652	p	K	1	16.4	3,573,153	3,573,380
RM6902	P	K	1	16.4–19.9	3,802,382	3,802,638
RM5302	P	K	1	20.2	4,149,094	4,149,317
RM3521	P	K	1	22.6–24.0	4,394,421	4,394,682
RM6463	p	K	1	22.6–24.0	4,432,422	4,432,645
RM8145	p	K	1	25.4		
RM8146	P	–	1	25.4		
RM5641	P	K	1	25.4–26.8	4,966,347	4,966,587
RM6259	P	K	1	25.4–26.8	4,983,970	4,984,196
RM3233	P	K	1	25.4–26.8	5,054,834	5,055,060
RM3604	P	K	1	26.8	5,139,280	5,139,505
RM3530	P	K	1	26.8	5,149,901	5,150,178
RM3360	P	K	1	27.3	5,236,387	5,236,616
RM1118	P	K	1	28.4–28.9	5,566,482	5,566,705
RM3746	P	K	1	30.5	6,154,949	6,155,182
RM1360	P	K	1	30.5	6,219,830	6,220,081
RM5443	P	K	1	30.5	6,224,491	6,224,724
RM8111	P	K	1	30.8		
RM6539	P	K	1	32.4–36.9	6,996,283	6,996,506
RM6786	P	K	1	32.4–36.9	7,075,295	7,075,518
RM1220	P	K	1	36.9	7,242,799	7,243,028
RM8098	P	K	1	36.9		
RM8077	P	K	1	37.4		
RM8078	P	K	1	37.4		
RM3598	P	K	1	38.8	7,347,806	7,348,031
RM5800	P	K	1	38.8	7,396,757	7,396,980
RM3235	P	K	1	38.8	7,516,125	7,516,350
RM3234	P	K	1	38.8	7,570,356	7,570,581
RM8143	p	K	1	38.8		
RM3780	p	K	1	42.4–43.2	8,017,941	8,018,176
RM5496	P	K	1	42.4–43.2	8,074,158	8,074,405
RM8141	P	K	1	43.2		
RM5346	p	K	1	45.4	8,750,010	8,750,235
RM8093	P	K	1	45.4–46.3		
RM8083	P	K	1	46.3		
RM3384	p	K	1	49	9,180,830	9,181,061
RM6466	p	K	1	49	9,191,124	9,191,347
RM6613	p	K	1	49.3	9,263,899	9,264,125
RM1032	p	K	1	49.3	9,317,405	9,317,632
RM8131	P	K	1	49.6		
RM8132	P	K	1	49.6		
RM8133	P	K	1	49.6		
RM6642	P	K	1	49.6–50.8	9,594,148	9,594,371
RM8070	P	K	1	49.6–50.8		
RM8071	P	K	1	49.6–50.8		
RM6039	p	K	1	50.8	9,710,984	9,711,210
RM6190	p	K	1	50.8	9,732,986	9,733,209
RM8051	P	K	1	52.1		
RM8052	P	K	1	52.1		
RM8053–1	P	K	1	52.1		
RM8045	P	K	1	52.4		
RM8046	P	K	1	52.4		
RM3627	P	K	1	54.3	10,306,915	10,307,140
RM1287–1	p	K	1	58.1	10,837,475	10,837,708
RM8094	P	K	1	60.6		
RM8095	P	K	1	60.6		
RM3412	P	K	1	62.5	11,566,862	11,567,095
RM5365	P	K	1	72.8	14,500,839	14,501,064
RM7075	P	K	1	73.1	15,101,695	15,101,946
RM6681	P	K	1	73.4	15,880,764	15,881,020
RM6711	P	K	1	73.4	16,094,680	16,094,903
RM5964	P	K	1	73.4	17,577,387	17,577,616
RM5422	P	K	1	73.7–78.0	18,752,836	18,753,067
RM6092	P	K	1	78	19,118,132	19,118,364
RM8139	P	K	1	78		
RM8004	P	K	1	78.1		
RM5638	P	K	1	86	20,931,939	20,932,177
RM6892	P	K	1	87.1	21,254,213	21,254,442
RM3341	P	K	1	87.4–90.0	21,883,862	21,884,091
RM7405	P	K	1	87.4–90.0	21,899,161	21,899,392
RM1196	P	K	1	87.4–90.0	21,900,409	21,900,636
RM6880	p	K	1	90	21,991,693	21,991,922
RM5718	P	K	1	94.5	22,923,359	22,923,648
RM5646	P	K	1	94.5–95.7	22,954,795	22,955,039
RM6716	P	K	1	95.7	23,116,909	23,117,135
RM8144	P	K	1	95.7		
RM6033	p	K	1	100.4–101.8	24,360,737	24,360,963
RM3682	P	K	1	100.4–101.8	24,371,946	24,372,175
RM7124	P	K	1	100.4–101.8	24,386,887	24,387,122
RM7266	P	K	1	100.4–101.8	24,446,211	24,446,442
RM5919	p	K	1	101.8–102.3	24,732,575	24,732,849
RM3642	P	K	1	102.3	24,862,256	24,862,483

RM1349-1	P	K	1	103.7	25,067,321	25,067,566
RM8129	P	K	1	103.7		
RM7318	P	K	1	110.2	26,136,714	26,136,937
RM3143	P	K	1	112.7	26,814,812	26,815,045
RM8128	P	K	1	112.7		
RM5461	p	K	1	113	26,900,351	26,900,588
RM3440	P	K	1	114.1	27,184,961	27,185,198
RM6738	P	K	1	114.1-116.5	27,504,650	27,504,915
RM5497	P	K	1	114.1-116.5	27,538,182	27,538,431
RM1232	p	K	1	116.5	27,626,812	27,627,041
RM7419	P	K	1	116.5	27,695,503	27,695,726
RM1297-1	P	K	1	121.6	28,606,463	28,606,698
RM3336	P	K	1	121.6	28,608,488	28,608,717
RM1244	P	K	1	122.1	28,684,383	28,684,612
RM3817	P	K	1	122.1	28,897,188	28,897,427
RM5931	P	K	1	122.6	29,277,928	29,278,241
RM1117	p	K	1	122.6	29,288,007	29,288,230
RM5486	P	K	1	122.6	29,362,170	29,362,413
RM8126	P	K	1	122.6		
RM1231	p	K	1	123.1	29,472,275	29,472,504
RM3547	p	K	1	123.1	29,472,359	29,472,582
RM6405	p	K	1	123.1	29,529,969	29,530,192
RM6702	P	K	1	123.2-123.5	29,763,406	29,763,641
RM3615	P	K	1	123.5	29,799,939	29,800,164
RM7371	P	K	1	123.5	29,800,877	29,801,100
RM3614	P	K	1	123.5	29,823,686	29,823,911
RM1061	p	K	1	124.8	30,171,860	30,172,095
RM1183	P	K	1	127.3	30,973,420	30,973,647
RM3411	P	K	1	129	31,305,594	31,305,827
RM1268	P	K	1	129	31,404,076	31,404,307
RM8096	P	K	1	129		
RM8097	P	K	1	129		
RM5914	P	K	1	129.3	1,631,848	1,632,116
RM6581	P	K	1	129.3	31,495,606	31,495,841
RM3324	p	K	1	130.1	31,713,093	31,713,320
RM3632	p	K	1	130.1-132.0	31,908,318	31,908,543
RM3709	P	K	1	130.1-132.0	31,941,688	31,941,917
RM1216	p	K	1	132	32,101,564	32,101,791
RM6648	p	K	1	132.8	32,331,565	32,331,788
RM3416	p	K	1	134.7-135.8	32,886,553	32,886,786
RM3285	p	K	1	135.8	33,043,964	33,044,189
RM8002	P	K	1	136.1		
RM8124	P	K	1	136.2		
RM1003	P	K	1	136.6	33,470,185	33,470,408
RM7180	P	K	1	136.9	34,098,712	34,098,935
RM8061	P	K	1	136.9		
RM6703	P	K	1	139.1	34,508,380	34,508,615
RM5811	P	K	1	139.1	34,629,679	34,629,908
RM6608	p	K	1	139.4	34,683,228	34,683,451
RM8084	P	K	1	140.2		
RM3304	P	K	1	140.2-140.5	34,949,799	34,950,026
RM3403	P	K	1	140.5	34,989,901	34,990,134
RM7594	P	K	1	140.5-141.6	35,465,411	35,465,628
RM5389	P	K	1	142.4	35,726,592	35,726,819
RM3640	P	K	1	142.4	35,831,691	35,831,918
RM7250	P	K	1	143.2	36,079,534	36,079,757
RM3738	P	K	1	143.7-144.5	36,927,811	36,928,042
RM6618	p	K	1	144.5	37,006,411	37,006,637
RM5448	P	K	1	146.4	37,391,539	37,391,774
RM1198	p	K	1	146.4	37,596,962	37,597,189
RM6333	P	K	1	147.2	38,002,891	38,003,114
RM1339-1	P	K	1	147.5	38,191,138	38,191,381
RM3442-1	P	K	1	147.5	38,196,724	38,196,961
RM6696	P	K	1	147.5	38,214,944	38,215,242
RM1068	P	K	1	150.7	38,432,397	38,432,636
RM6504	P	K	1	154.6	38,979,878	38,980,101
RM3602	P	K	1	155.2	39,001,828	39,002,053
RM5759	p	K	1	155.2	39,013,725	39,013,948
RM7246	P	K	1	157.1	39,164,091	39,164,318
RM6827	P	K	1	157.1	39,197,070	39,197,302
RM8062	P	K	1	157.1		
RM8066	P	K	1	157.1		
RM8067	P	K	1	157.1		
RM3810	P	K	1	157.6	39,485,367	39,485,606
RM3523	P	-	1	157.6	39,817,281	39,817,544
RM3468-1	P	K	1	159.6	40,136,256	40,136,497
RM1387	P	K	1	159	40,200,960	40,201,247
RM3520-1	P	K	1	159.6	40,246,914	40,247,175
RM5362	P	K	1	163.8	41,080,377	41,080,602
RM5410	p	K	1	163.8	41,088,099	41,088,328
RM8099	P	K	1	163.8		
RM5310	P	K	1	164.1	41,190,655	41,190,890
RM8089	P	K	1	167.2-169.5		
RM6321	P	K	1	181.1	42,916,492	42,916,733
RM1067	P	K	1	181.1	42,916,739	42,916,978
RM3362	P	K	1	181.8	43,036,305	43,036,533
RM6840	P	K	1	181.8	43,165,074	43,165,324
RM8135	P	K	1	181.8		
RM8136	P	K	1	181.8		
RM8137	P	K	1	181.8		
RM6143	p	K	2	0	73,456	73,682
RM6842	P	K	2	0	124,579	124,841
RM7451	P	K	2	2.5-4.7	651,794	652,025
RM6938	P	K	2	4.7-6.9	977,699	978,012
RM6367	P	K	2	4.7-6.9	1,132,184	1,132,425
RM7562	p	K	2	8.9	1,878,293	1,878,520
RM3096	P	K	2	13.4	2,835,481	2,835,613
RM6616	P	K	2	13.4	2,864,997	2,865,224
RM3188	P	K	2	15	3,453,704	3,453,927
RM1075	p	K	2	17.9	3,833,199	3,833,442
RM1285	p	K	2	17.9	3,837,883	3,838,116
RM3703	p	K	2	17.9	3,863,713	3,863,942
RM7581	P	K	2	19.0-20.9	4,206,244	4,206,483

RM5996	p	K	2	19.0-20.9	4,208,497	4,208,720
RM5512	P	K	2	19.0-20.9	4,221,150	4,221,373
RM4355	P	K	2	19.0-20.9	4,264,218	4,264,455
RM5622	P	K	2	19.0-20.9	4,273,659	4,273,891
RM3732	P	K	2	20.9	4,407,819	4,408,050
RM6641	P	K	2	23.3	4,633,939	4,634,180
RM3497	P	K	2	23.3	4,699,248	4,699,497
RM6654	P	K	2	23.3-25.5	4,816,938	4,817,161
RM4949	P	K	2	23.3-25.5	4,835,690	4,835,953
RM5350	p	K	2	23.3-25.5	4,911,308	4,911,533
RM2008	P	K	2	23-25	4,934,093	4,934,330
RM7082	P	K	2	25.5	5,100,890	5,101,113
RM3294	P	K	2	25.5	5,204,927	5,205,154
RM1347	P	K	2	26.9	5,314,354	5,314,603
RM5664	p	K	2	26.9	5,379,400	5,379,626
RM5780	P	K	2	26.9	5,453,123	5,453,364
RM6378	P	K	2	28	5,477,465	5,477,721
RM4702	P	K	2	31.2	6,218,964	6,219,213
RM5897	p	K	2	32.8	6,732,589	6,732,833
RM2939	p	K	2	33.6-36.3	7,420,111	7,420,388
RM2468	P	K	2	36.3	7,420,437	7,420,680
RM5340	P	K	2	36.3	7,482,266	7,482,489
RM2814	P	K	2	36.3	7,486,314	7,486,591
RM5459	p	K	2	36.3	7,518,136	7,518,371
RM3828	P	K	2	36.3-36.8	7,645,604	7,645,845
RM7636	P	K	2	36.3-36.8	7,663,243	7,663,486
RM3390	p	K	2	36.3-36.8	7,663,463	7,663,694
RM6853	P	K	2	42.1	8,955,928	8,956,151
RM5699	P	K	2	42.1	8,980,479	8,980,738
RM6911	p	K	2	42.1	9,008,053	9,008,321
RM7288	P	K	2	42.1	9,032,605	9,032,884
RM6639	P	K	2	42.4-43.4	9,415,458	9,415,681
RM6375	p	K	2	43.4-47.8	9,554,650	9,554,903
RM1358	P	K	2	48.1	10,184,619	10,184,866
RM3501	P	K	2	48.1	10,186,608	10,186,857
RM2792	P	K	2	50.3	10,754,117	10,754,386
RM1106	p	K	2	50.3	10,996,526	10,996,749
RM5015	P	K	2	50.3	25,214,230	25,214,395
RM5862	P	K	2	58.4	6,005,859	6,006,184
RM5791	p	K	2	50.3	10,746,744	10,746,967
RM5390	p	N	2	50.3	10,806,207	10,806,434
RM1081	P	K	2	50.8	11,182,475	11,182,728
RM1313	P	K	2	51.1	11,261,996	11,262,233
RM5619	P	K	2	52.2	12,009,734	12,009,963
RM5439	p	K	2	52.2-53.5	12,098,978	12,099,211
RM5210	p	K	2	54.6	12,665,916	12,666,201
RM3443	P	K	2	54.6	14,430,889	14,431,126
RM6374	p	K	2	54.6	15,287,698	15,287,945
RM5521	p	K	2	54.9-55.4	15,951,309	15,951,532
RM5812	P	K	2	55.4	15,999,785	16,000,017
RM7426	p	K	2	57.3	16,778,169	16,778,392
RM5101	P	K	2	58.4-59.5	18,161,640	18,161,910
RM6844	p	K	2	58.4-59.5	18,169,315	18,169,604
RM1211-1	P	K	2	59.5-62.2	18,556,018	18,556,245
RM7413	P	K	2	59.5-62.2	18,558,423	18,558,646
RM4499	P	K	2	62.2	18,635,391	18,635,642
RM5578-1	P	K	2	62.2-64.7	18,738,310	18,738,483
RM3858	P	K	2	62.2-71.3	19,208,110	19,208,361
RM6509	P	K	2	71.3	19,361,543	19,361,766
RM6380	P	K	2	71.3-77.8	19,768,222	19,768,502
RM1379	P	K	2	77.8-79.6	20,076,041	20,076,304
RM2634	P	K	2	80.5-81.4	20,520,494	20,520,759
RM3666	p	K	2	81.7	20,922,006	20,922,233
RM6023	p	K	2	81.7	20,996,865	20,997,088
RM5614	P	K	2	81.7-83.6	21,080,751	21,080,977
RM5427	P	K	2	85.9	21,544,286	21,544,517
RM5789	P	K	2	88.2	22,409,717	22,409,940
RM3688	P	K	2	88.2	22,420,121	22,420,350
RM3762-1	P	K	2	88.2-89.9	22,481,790	22,482,025
RM3352	P	K	2	93.2	23,592,372	23,592,601
RM3515-1	P	K	2	95.5	24,041,789	24,042,044
RM1942	p	K	2	102.6	25,337,550	25,337,687
RM1920	P	K	2	103.4	25,485,748	25,485,983
RM1307	p	K	2	103.9-105.8	25,849,777	25,850,012
RM3730	p	K	2	106.6	26,260,832	26,261,057
RM5706	P	K	2	109.3	26,503,898	26,504,160
RM4472	P	K	2	109.3	26,572,908	26,573,148
RM7511	p	K	2	109.3	26,626,615	26,626,870
RM1385	p	K	2	109.3	26,664,906	26,665,183
RM1367-1	P	K	2	110.6	27,083,759	27,084,012
RM3508	P	K	2	110.9	27,101,283	27,101,534
RM5470-1	P	K	2	111.2-112.6	27,172,400	27,172,639
RM3512-1	P	K	2	112.6	27,339,280	27,339,535
RM3763	P	K	2	114.0-118.1	27,950,071	27,950,306
RM3289	P	K	2	114.0-118.1	27,967,247	27,967,474
RM1342-1	P	K	2	114.0-118.1	28,183,765	28,184,008
RM5631	P	K	2	118.1	28,291,905	28,292,140
RM6465	P	K	2	118.1	28,381,313	28,381,536
RM3685	P	K	2	123.9	29,313,202	29,313,431
RM6933-1	P	K	2	123.9	29,331,877	29,332,178
RM6424	P	K	2	125.6	29,644,314	29,644,540
RM5303	p	K	2	126.4	29,857,607	29,857,830
RM5378	p	K	2	126.4	29,886,347	29,886,574
RM6290	p	K	2	126.4	29,895,626	29,895,855
RM3275	p	K	2	128.3	30,128,666	30,128,891
RM5472	P	K	2	130.2	30,661,496	30,661,735
RM8024	p	K	2	130.2-131.8		
RM6627	p	K	2	138.0-140.9	32,765,727	32,765,950
RM5958	p	K	2	138.0-140.9	32,859,599	32,859,822
RM8029	P	K	2	138-140.9		
RM8030	P	K	2	138-140.9		
RM3212	p	K	2	150.5	28,885,630	28,885,853
RM5833	p	K	2	150.5	28,891,024	28,891,271

RM7009	p	K	2	141.7	32,984,925	32,985,148
RM7286	P	K	2	141.7	33,018,942	33,019,205
RM6733	p	K	2	141.7	33,019,361	33,019,620
RM6636	p	K	2	141.7-142.5	33,097,317	33,097,546
RM5460	p	K	2	144.7-146.6	33,791,476	33,791,713
RM1092	P	K	2	144.7-146.6	33,872,124	33,872,347
RM5300	P	K	2	144.7-146.6	33,882,838	33,883,061
RM5421	p	K	2	151.6	34,707,935	34,708,166
RM3789-1	p	K	2	150.5	34,709,081	34,709,320
RM3535	P	-	2	150.5	34,713,756	34,713,979
RM3542	P	K	2	154.1	35,162,893	35,163,116
RM1255	p	K	2	155.5	35,280,259	35,280,490
RM6312	p	K	2	156.3	35,409,179	35,409,411
RM2265	p	K	2	156.3	35,424,268	35,424,515
RM3850	p	K	2	157.9	35,450,087	35,450,334
RM7388	p	K	2	157.9	35,496,843	35,497,066
RM4470	p	K	2	157.9	35,562,394	35,562,533
RM5643	p	K	2	157.9	35,578,313	35,578,557
RM7337	P	K	2	157.9	35,627,914	35,628,141
RM3274	P	K	2	157.9-	35,729,115	35,729,340
RM6139	p	K	2	157.9	35,863,439	35,863,665
RM5807	p	K	2	157.9	35,873,088	35,873,314
RM3265	p	K	3	0.0-1.1	82,175	82,400
RM3413	P	K	3	0.0-1.1	347,739	347,972
RM3387	p	K	3	0.0-1.1	359,398	359,621
RM7332	P	K	3	0.0-1.1	390,495	390,742
RM4108	P	K	3	1.1	514,807	515,034
RM3203	P	K	3	2.2	776,074	776,297
RM3202	p	K	3	2.2	789,282	789,505
RM3894	P	K	3	2.5	1,097,600	1,097,829
RM4853	P	K	3	6	1,376,815	1,377,070
RM3372	P	K	3	6	1,437,106	1,437,337
RM7072	P	K	3	13.3	2,552,210	2,552,457
RM6829	P	K	3	14.4	2,862,108	2,862,340
RM4683	P	K	3	14.8	3,162,565	3,162,822
RM6849	P	K	3	15.2	3,268,540	3,268,763
RM3126	P	K	3	16.8	3,661,177	3,661,404
RM5474	P	K	3	16.8-17.9	3,785,867	3,786,108
RM3392	P	K	3	17.9	3,808,695	3,808,928
RM5819	P	K	3	18.4-20.3	4,268,176	4,268,411
RM4352	P	K	3	18.4-20.3	4,296,887	4,297,120
RM3195	P	K	3	21.5	4,464,394	4,464,617
RM4992	P	K	3	22.1	4,706,012	4,706,275
RM6038	p	K	3	24.4	4,811,135	4,811,361
RM5480	P	K	3	99	5,306,022	5,306,263
RM5442	P	K	3	25.9-27.9	5,496,736	5,496,969
RM5444	P	K	3	25.9-27.9	5,559,867	5,560,100
RM3417	p	K	3	27.9	5,617,483	5,617,716
RM3864	P	K	3	28.1	5,828,327	5,828,584
RM5570	P	K	3	28.1	5,840,126	5,840,361
RM5755	P	K	3	28.2	5,927,260	5,927,537
RM3467	P	K	3	28.5	5,971,918	5,972,159
RM1324	P	K	3	28.5	6,004,608	6,004,847
RM7576	P	K	3	28.5	6,046,694	6,046,925
RM5347	p	K	3	30.5	6,396,098	6,396,323
RM5477	p	K	3	31.1	6,521,156	6,521,397
RM3441	P	K	3	31.1	6,550,779	6,551,016
RM3029	P	K	3	31.3	6,676,577	6,676,862
RM5925-1	P	K	3	31.3-33.5	6,769,689	6,769,975
RM6291	P	K	3	31.3-33.5	6,779,869	6,780,098
RM3716	p	K	3	33.5	6,807,809	6,807,986
RM3872	P	K	3	33.5	6,820,447	6,820,718
RM3766-1	P	K	3	34.8	6,902,036	6,902,271
RM1002	p	K	3	36.1	7,110,431	7,110,654
RM6308	p	K	3	36.1	7,148,579	7,148,805
RM1022	p	K	3	36.9	7,201,275	7,201,500
RM3134	p	K	3	36.9	7,208,899	7,209,126
RM3545	p	K	3	36.9	7,292,627	7,292,850
RM6417	p	K	3	39.0-40.3	8,076,902	8,077,125
RM1338-1	P	K	3	40.3	8,362,219	8,362,460
RM4996	P	K	3	40.3	8,452,116	8,452,383
RM5896	P	K	3	40.3	8,515,258	8,515,499
RM5686-1	P	K	3	40.3	8,515,300	8,515,550
RM6783	P	K	3	43.5	9,285,953	9,286,182
RM1256	p	K	3	44.1-44.4	9,714,194	9,714,425
RM1371	P	K	3	44.4-46.6	9,912,283	9,912,538
RM2790	P	K	3	49.3-50.8	4,505,358	4,505,491
RM4321	P	K	3	46.6	10,140,187	10,140,320
RM3434	P	K	3	48.5	10,745,699	10,745,934
RM3280	P	K	3	48.8	10,874,641	10,874,866
RM7365	p	K	3	49.3	11,249,158	11,249,401
RM4512	P	K	3	50.3	11,279,913	11,280,055
RM3562	p	K	3	50.8-54.7	11,654,458	11,654,681
RM5748	p	K	3	54.7-55.8	12,297,668	12,297,903
RM5928-1	P	K	3	56.3	12,547,308	12,547,609
RM5551	P	K	3	59.5	12,901,279	12,901,508
RM7323	P	K	3	59.5-61.9	12,943,737	12,943,964
RM5419	P	K	3	59.5-61.9	12,997,973	12,998,204
RM1319	p	K	3	59.5-61.9	12,998,170	12,998,409
RM5903	P	K	3	61.9	13,253,598	13,253,851
RM5489	P	K	3	61.9	13,278,065	13,278,311
RM3291	P	K	3	61.9-63.3	13,574,445	13,574,672
RM6080	p	K	3	63.3	13,768,001	13,768,227
RM6676-1	P	K	3	65.4	14,327,586	14,327,821
RM6959	P	K	3	65.4	14,340,966	14,341,192
RM7369	p	K	3	65.4-68.7	14,531,873	14,532,096
RM3204	P	K	3	69.2	14,825,418	14,825,641
RM3400	p	K	3	84.4	17,071,966	17,072,199
RM6914	P	K	3	84.4	17,098,188	17,098,459
RM1334	P	K	3	84.4-85.2	17,228,387	17,228,628
RM2346	P	K	3	85.2	17,501,166	17,501,415
RM1940	P	K	3	86	17,968,464	17,968,705
RM7642	p	K	3	86	18,437,963	18,438,190

RM2453	P	K	3	86	20,046,012	20,046,165
RM7370	p	K	3	86.5	20,951,356	20,951,579
RM5488	P	K	3	87.1	21,193,732	21,193,977
RM3646	P	K	3	87.9	21,798,652	21,798,879
RM7134	P	K	3	87.9	21,813,779	21,814,004
RM6832	P	K	3	88.7-89.2	22,203,275	22,203,510
RM5532	P	K	3	91.1-94.9	23,214,450	23,214,675
RM6266	P	K	3	94.9	23,624,297	23,624,523
RM5626-1	P	K	3	99	24,671,160	24,671,392
RM3513	P	K	3	99.6	24,919,450	24,919,705
RM2334	P	K	3	115.6	26,547,297	26,547,546
RM7097	p	K	3	115.6-119.7	26,680,895	26,681,122
RM6736	P	K	3	120.1-120.4	27,123,477	27,123,736
RM5286	p	K	3	120.1-120.4	27,124,226	27,124,336
RM6053	p	K	3	120.4	27,187,804	27,188,033
RM2614	P	K	3	120.4	27,226,150	27,226,409
RM1350-1	P	K	3	126.8	28,479,732	28,479,977
RM3856-1	P	K	3	127.1	28,578,685	28,578,934
RM3525	P	K	3	136.5	30,192,760	30,193,025
RM3199	p	K	3	137.6	30,221,109	30,221,332
RM5172	p	K	3	137.6	30,227,926	30,228,201
RM5992	p	K	3	137.9	30,321,746	30,321,969
RM5475-1	p	K	3	137.9	30,375,989	30,376,230
RM3829	P	K	3	138.7	30,629,149	30,629,328
RM5995	p	K	3	139.8	30,744,563	30,744,786
RM5813	p	K	3	139.8	30,780,012	30,780,244
RM6781	P	K	3	139.8	30,840,136	30,840,365
RM3867	P	K	3	142.3	31,539,971	31,540,230
RM6508	P	K	3	144.5	31,777,994	31,778,217
RM1238	P	K	3	144.5	31,870,830	31,871,059
RM6090-1	p	K	3	144.5	31,995,629	31,995,858
RM6759	p	K	3	144.5	32,016,762	32,017,201
RM6970	P	K	3	146.1	32,230,635	32,230,870
RM3346	p	N	3	151.5	33,111,110	33,111,339
RM3564	p	N	3	151.5	33,218,161	33,218,384
RM1373	P	N	3	151.1	33,502,902	33,503,169
RM7000	P	N	3	151.5-152.0	33,589,153	33,589,400
RM6712	P	K	3	157.7	35,019,905	35,020,128
RM6135	P	K	3	157.7	35,050,523	35,050,746
RM2187	P	K	3	158.2	35,403,133	35,403,378
RM1221	P	K	3	159	35,462,884	35,463,113
RM7389	P	K	3	164.4	35,948,417	35,948,644
RM1867	p	K	4	5.4-6.5	913,815	914,037
RM2416	P	K	4	6.5	941,058	941,306
RM3892	P	K	4	6.8	1,123,538	1,123,705
RM7535	p	K	4	6.8	1,159,274	1,159,505
RM5414	P	K	4	7.9	2,021,661	2,021,890
RM2811	P	K	4	7.9	2,074,173	2,074,440
RM6770	p	K	4	8.7-10.1	2,813,060	2,813,283
RM7200	P	K	4	10.1-11.0	4,019,201	4,019,432
RM3658	p	K	4	14.3-16.7	5,573,576	5,573,803
RM1305	p	K	4	14.3-16.7	5,624,391	5,624,626
RM3471	P	K	4	16.7-18.3	6,279,384	6,279,625
RM6659	P	K	4	16.7-18.3	6,541,721	6,541,962
RM7279	p	K	4	23.3	13,825,426	13,825,669
RM5687	P	K	4	25.4	15,927,714	15,927,964
RM3308	p	K	4	44.0-48.3	19,186,523	19,186,750
RM1205	p	K	4	49.7-52.6	19,643,655	19,643,882
RM5586	P	K	4	52.6-56.1	19,908,585	19,908,844
RM3742	p	K	4	52.6-56.1	19,925,163	19,925,396
RM1359-1	p	K	4	56.1	20,041,055	20,041,304
RM3643	p	K	4	57.5	20,128,592	20,128,819
RM5749	P	K	4	57.5	20,131,093	20,131,316
RM5424	p	K	4	58.9-60.2	20,608,484	20,608,715
RM3397	P	K	4	58.9-60.2	20,633,258	20,633,487
RM7313	p	K	4	60.2	20,678,759	20,678,982
RM3708	P	K	4	60.2-62.1	20,839,761	20,839,990
RM5635	P	K	4	60.2-62.1	20,889,764	20,889,999
RM5757	p	K	4	62.6	21,425,081	21,425,304
RM3042	P	K	4	70.1	22,992,712	22,992,997
RM2521	P	K	4	70.1-70.6	23,193,020	23,193,175
RM3866	P	K	4	70.6	23,333,612	23,333,869
RM6351	P	K	4	70.6	23,395,720	23,395,946
RM6540	p	K	4	72	23,601,879	23,602,102
RM3785	P	K	4	74.5	24,222,826	24,223,063
RM3367	P	K	4	74.5	24,260,161	24,260,392
RM7051	P	K	4	74.5	24,277,159	24,277,386
RM5270	P	K	4	76.8	24,989,024	24,989,169
RM1388-1	P	K	4	77.9	25,195,510	25,195,801
RM1223-1	P	K	4	78.2	25,451,328	25,451,557
RM1354	P	K	4	78.2-81.7	26,272,426	26,272,671
RM3735	p	K	4	81.7	26,369,272	26,369,503
RM5714-1	P	K	4	84.1-85.2	27,446,610	27,446,893
RM3288-1	p	K	4	85.2	27,516,134	27,516,361
RM6454	p	K	4	85.2	27,541,137	27,541,360
RM3092	P	K	4	85.5	27,691,541	27,691,684
RM3820	P	K	4	85.5-87.1	27,782,114	27,782,353
RM2636	P	K	4	87.1	28,004,458	28,004,575
RM2441	P	K	4	87.1	28,020,750	28,021,003
RM7210	P	K	4	87.1-94.4	28,613,163	28,613,398
RM3781	P	K	4	87.1-94.4	28,731,315	28,731,550
RM3916	P	K	4	87.1-94.4	28,733,205	28,733,456
RM6089	P	K	4	97.7	29,563,436	29,563,665
RM6480	P	K	4	97.7-99.3	29,852,136	29,852,362
RM7208	P	K	4	99.3-100.7	30,175,635	30,175,865
RM5503-1	P	K	4	100.7	30,397,605	30,397,858
RM3276	p	K	4	102.1	30,715,662	30,715,887
RM6748	P	K	4	102.7	30,902,069	30,902,346
RM5511	P	K	4	102.7-107.4	31,146,772	31,147,072
RM3534	P	K	4	102.7-107.4	31,197,968	31,198,191
RM1250	P	K	4	102.7-107.4	31,296,033	31,296,264
RM3687	p	K	4	107.4	31,517,487	31,517,716
RM5473-1	P	K	4	108.2	31,710,397	31,710,636

RM3843	P	K	4	108.2	31,717,525	31,717,770
RM6629	P	K	4	108.2	31,778,484	31,778,707
RM3836	P	K	4	108.2-109.9	31,845,434	31,845,677
RM5709-1	P	K	4	109.9	32,094,491	32,094,762
RM6909	P	K	4	109.9	32,094,493	32,094,764
RM3335-1	p	K	4	113.2	33,060,223	33,060,452
RM1153	p	K	4	113.2	33,065,542	33,065,767
RM3306	p	K	4	113.2	33,106,363	33,106,590
RM3648	p	K	4	114.3	33,340,360	33,340,587
RM5506	P	K	4	114.3-120.3	33,526,172	33,526,433
RM3466	P	K	4	120.3-122.9	34,083,728	34,083,965
RM1113	P	K	4	122.9	34,304,852	34,305,075
RM2799	P	K	4	122.9	34,354,663	34,354,932
RM1112	P	K	4	123.8-128.5	34,456,250	34,456,473
RM7172	P	K	4	123.8-128.5	34,630,150	34,630,373
RM3310	P	K	4	123.8-128.5	34,765,869	34,766,096
RM3399	P	K	4	128.5	34,916,550	34,916,783
RM6006	P	K	4	128.5	34,927,078	34,927,301
RM3332	p	K	4	128.5	34,936,709	34,936,938
RM6473	p	K	4	129.1	35,024,170	35,024,396
RM1841	P	K	4	129.6	35,139,756	35,139,983
RM2431	P	K	4	129.6	35,139,856	35,139,983
RM7030	P	K	4	129.6	35,411,625	35,411,856
RM1248	P	K	5	0-0.3	62,566	62,795
RM3529	P	K	5	0-3.0	82,024	82,301
RM4777	P	K	5	4.6	422,042	422,303
RM5693	P	K	5	4.6	432,449	432,702
RM3796	P	K	5	4.6	456,790	457,027
RM7029	P	K	5	6.5	537,755	537,982
RM6300	P	K	5	6.5	568,859	569,082
RM1258	P	K	5	9.5	782,886	783,117
RM1200	P	K	5	9.5	804,892	805,119
RM2010	P	K	5	12	1,120,521	1,120,758
RM1024	P	K	5	14.1	1,174,275	1,174,500
RM6317	P	K	5	14.1-17.9	1,462,168	1,462,403
RM5579-1	P	K	5	19.0-20.1	1,855,846	1,856,089
RM7302	p	K	5	20.1	1,917,441	1,917,664
RM3345	p	K	5	20.1	1,951,290	1,951,519
RM2488	P	K	5	21.1	2,085,089	2,085,344
RM4710	P	K	5	21.1	34,876,210	34,876,459
RM6517	p	K	5	24.7	2,853,215	2,853,438
RM1366	P	K	5	24.7	2,855,477	2,855,728
RM3322	p	K	5	32	4,203,647	4,203,874
RM5994	p	K	5	45.8	6,798,566	6,798,789
RM4837	P	K	5	46.9	6,932,208	6,932,463
RM2422	P	K	5	46.9	6,937,938	6,938,189
RM4691	P	K	5	46.9	6,941,234	6,941,483
RM6034	p	K	5	46.9	6,949,404	6,949,630
RM3683	p	K	5	47.2	7,091,499	7,091,728
RM2998	P	K	5	47.2	7,150,222	7,150,496
RM3381	P	K	5	53.5-54.3	9,485,561	9,485,792
RM2676	P	K	5	54.6	13,403,771	13,404,034
RM5140	P	K	5	54.6	13,408,182	13,408,449
RM6229	P	K	5	54.6-55.4	13,472,890	13,473,125
RM6724	P	K	5	54.6-55.4	13,697,171	13,697,409
RM6742	P	K	5	55.4-58.7	14,743,587	14,743,852
RM6645	P	K	5	58.7	14,944,703	14,944,926
RM6024	p	K	5	67.5	17,668,464	17,668,687
RM6025	P	K	5	67.5	17,725,795	17,726,018
RM1237	P	K	5	67.5	17,872,276	17,872,505
RM4743	p	K	5	70.5-73.9	18,293,492	18,293,741
RM1868	P	K	5	70.5-73.9	18,314,564	18,314,797
RM6621	P	K	5	70.5-73.9	18,698,659	18,698,888
RM7568	p	K	5	75	19,346,191	19,346,414
RM3160	P	K	5	75.0-77.4	19,899,341	19,899,616
RM1386	P	K	5	75.0-77.4	19,912,853	19,913,136
RM5329	P	K	5	75.0-77.4	19,947,140	19,947,363
RM4674	P	K	5	77.4-78.2	20,179,619	20,179,866
RM3351-1	P	K	5	80.7	20,614,960	20,615,189
RM6954	p	K	5	89.6	21,936,521	21,936,744
RM4501	P	K	5	89.6	21,968,652	21,968,891
RM5592	P	K	5	89.6	21,970,375	21,970,684
RM5401	P	K	5	92	22,144,559	22,144,786
RM3295	P	K	5	92.0-94.5	22,187,343	22,187,570
RM3759	P	K	5	92.0-94.5	22,246,799	22,247,032
RM1271	P	K	5	92.0-94.5	22,333,136	22,333,367
RM6841	P	K	5	95.3	22,657,355	22,657,614
RM6054	P	K	5	95.3	22,696,622	22,696,857
RM3870	P	K	5	95.8	22,817,695	22,817,958
RM5311	P	K	5	95.8	22,828,446	22,828,669
RM5575	P	K	5	96.4	23,264,008	23,264,247
RM6598	P	K	5	101.5-102.8	23,980,978	23,981,201
RM6545	P	K	5	103.9	24,789,244	24,789,470
RM3348	P	K	5	104.4	24,948,876	24,949,105
RM3620	P	K	5	104.4	25,065,844	25,066,069
RM6972	P	K	5	104.7	25,208,247	25,208,485
RM3321	p	K	5	107.4	25,645,910	25,646,137
RM3616	p	K	5	108.5	26,137,352	26,137,577
RM3809	P	K	5	108.5	26,444,182	26,444,421
RM2357	P	K	5	109	26,712,644	26,712,893
RM7452	p	K	5	110.4	26,782,938	26,783,173
RM7473	p	K	5	110.4	26,801,148	26,801,399
RM5784	P	K	5	112.4-115.7	27,637,400	27,637,656
RM3170	P	K	5	115.7	27,796,335	27,796,558
RM6360-1	p	K	5	115.7	27,924,566	27,924,798
RM6339	p	K	5	116.2	27,931,838	27,932,061
RM6313	P	K	5	116.5	28,120,099	28,120,331
RM6822	P	K	5	122.2	29,041,757	29,041,983
RM3068	P	K	5	122.3	29,093,932	29,094,224
RM8039	P	K	5	55		
RM6273	P	N	6	0.6	130,334	130,566
RM6775	P	N	6	0.6	208,954	209,177
RM7158	P	N	6	0.6	216,256	216,503

RM6467	P	N	6	0.6	219,965	220,188
RM8116	P	N	6	0.9		
RM8117	P	N	6	0.9		
RM8120	P	N	6	0.9		
RM8121	P	N	6	0.9		
RM3353	P	N	6	1.4	435,548	435,777
RM8107	P	N	6	1.7		
RM8109	P	N	6	1.7		
RM4332	P	N	6	3.1	720,912	721,046
RM8060	P	N	6	3.1		
RM2353	P	N	6	3.6	1,064,340	1,064,597
RM8072	P	N	6	6		
RM1369	P	N	6	6.6	1,562,558	1,562,811
RM8101	P	N	6	6.9		
RM5199	P	N	6	10.4	2,270,117	2,270,231
RM3805	P	N	6	11.5	2,853,003	2,853,240
RM3414	P	N	6	11.5	2,881,700	2,881,933
RM8125	P	N	6	12.9		
RM6917	P	N	6	15.5	4,079,317	4,079,591
RM4608	P	N	6	15.5	4,314,970	4,315,215
RM6176	P	N	6	15.8-18.0	4,623,091	4,623,314
RM6194	P	H	6	32.7	6,069,977	6,070,200
RM7488-1	P	N	6	38.3-40.2	7,010,907	7,011,134
RM3431-1	P	H	6	51.0-53.5	8,756,703	8,756,937
RM3431A	P	H	6	51.0-53.5	8,756,703	8,756,937
RM5963-1	P	H	6	51.0-53.5	8,826,389	8,826,615
RM6302	P	K	6	53.5	8,984,467	8,984,690
RM6162	P	K	6	53.8	9,210,045	9,210,274
RM6836-1	P	K	6	54.1	9,320,722	9,320,963
RM7311	P	K	6	58.7-61.6	10,889,390	10,889,613
RM3183	P	K	6	64.6	12,290,840	12,291,063
RM5745	p	K	6	64.9	12,336,809	12,337,035
RM1161	P	K	6	65.8	13,595,858	13,596,083
RM6818	p	K	6	65.8	16,364,235	16,364,461
RM5087	P	K	6	65.8	16,459,600	16,459,869
RM3207	P	K	6	65.8	17,517,626	17,517,765
RM7193	P	K	6	70.9	19,910,824	19,911,051
RM3187	p	K	6	72.9	20,578,902	20,579,125
RM3498	P	K	6	73.2	20,634,084	20,634,333
RM3827-1	P	K	6	79	21,950,302	21,950,543
RM1340-1	P	K	6	82.9	22,960,533	22,960,776
RM3272	P	K	6	84.5-85.4	23,237,160	23,237,385
RM6298	P	K	6	85.4	23,353,466	23,353,689
RM7434	P	K	6	87.5	23,552,127	23,552,366
RM6202	P	K	6	87.5-90.5	23,878,153	23,878,376
RM3279	P	K	6	87.5-90.5	23,942,014	23,942,239
RM5957	P	K	6	90.5	24,138,791	24,139,014
RM5371	P	K	6	96.5	25,442,711	25,442,936
RM6395	P	K	6	99.2	25,612,810	25,613,039
RM6782	P	K	6	99.2	25,662,572	25,662,801
RM7309	p	K	6	100.3	25,914,609	25,914,836
RM6274	P	K	6	100.3	25,935,738	25,935,970
RM3138	P	K	6	110.6	28,086,362	28,086,593
RM7555-1	P	K	6	117	29,054,809	29,055,056
RM3343	P	K	6	117	29,099,681	29,099,910
RM3765	P	K	6	117.0-119.2	29,207,571	29,207,806
RM3509	P	K	6	124.4	30,456,026	30,456,277
RM5463-1	p	K	6	124.4	30,470,062	30,470,299
RM4584	P	K	7	2.2	494,849	495,092
RM6047	P	K	7	3.9	741,838	742,067
RM7454	P	K	7	4.4	1,102,252	1,102,479
RM3831	P	K	7	5.2	1,164,715	1,164,958
RM1085	P	K	7	5.8	1,243,095	1,243,354
RM3222	P	K	7	6.4-7.0	1,733,194	1,733,417
RM5100	P	K	7	9.6	2,249,684	2,249,953
RM3325	P	K	7	9.6	2,250,349	2,250,576
RM5752-1	P	K	7	11	2,599,925	2,600,163
RM5055	P	K	7	11	2,682,147	2,682,414
RM5711-1	p	K	7	24.2	3,174,256	3,174,527
RM1353-1	P	K	7	24.8	3,344,916	3,345,163
RM1243	P	K	7	25.4	3,587,770	3,587,999
RM7479-1	p	K	7	31	4,167,672	4,167,895
RM2006	P	K	7	31.0-35.7	4,230,099	4,230,336
RM3484	P	K	7	31.0-35.7	4,452,024	4,452,267
RM8008	P	K	7	31.0-35.7		
RM8009	P	K	7	31.0-35.7		
RM6872	P	K	7	35.7	4,692,510	4,692,733
RM6574	P	K	7	38.4	4,714,940	4,715,166
RM7121	P	K	7	41.7	5,656,003	5,656,226
RM6728	P	K	7	41.7-42.6	5,762,130	5,762,377
RM5672	P	K	7	44.1	6,413,118	6,413,356
RM1253	p	K	7	45.2	7,000,946	7,001,177
RM8006	P	K	7	46.5-47.1		
RM7153	P	K	7	48.0-49.4	8,640,555	8,640,786
RM3859	p	K	7	49.4	8,910,348	8,910,603
RM7074	p	K	7	49.4	9,043,858	9,044,109
RM5436	p	K	7	49.7	9,107,816	9,108,049
RM5499	p	K	7	49.7	10,020,317	10,020,566
RM3635	P	K	7	49.7	11,120,425	11,120,650
RM3832	P	K	7	49.7	12,659,408	12,659,651
RM1377	P	K	7	49.7	12,730,866	12,731,129
RM7273	P	K	7	50	13,377,037	13,377,268
RM3670	P	K	7	50	13,386,282	13,386,509
RM8036	p	K	7	50		
RM8037	p	K	7	50		
RM7184	p	K	7	50.0-50.9	14,489,038	14,489,261
RM2878	p	K	7	50.0-50.9	14,584,027	14,584,300
RM3755	P	K	7	50.9	14,680,895	14,681,128
RM5543	P	K	7	53.4-55.6	15,699,515	15,699,742
RM5481-1	P	K	7	55.6	16,146,408	16,146,649
RM7110-1	p	K	7	56.2	16,800,663	16,800,898
RM1135	p	K	7	57.5	16,879,357	16,879,580
RM3795	P	K	7	57.5-60.8	17,132,816	17,133,053

RM6767-1	P	K	7	60.8	17,417,522	17,417,781
RM6394	P	K	7	61.9	18,340,234	18,340,460
RM3300	P	K	7	62.4-67.0	18,908,937	18,909,164
RM3691	P	K	7	67	19,172,490	19,172,719
RM3743	p	K	7	67	19,290,447	19,290,680
RM5420	P	K	7	67	19,304,394	19,304,621
RM7087	P	K	7	67	19,304,765	19,304,988
RM5583	P	K	7	67	19,307,849	19,308,100
RM2966	P	K	7	67.0-69.2	19,681,525	19,681,713
RM3404-1	P	K	7	70	20,053,245	20,053,478
RM1279	P	K	7	75.6	21,561,321	21,561,554
RM3799	p	K	7	76.2	21,578,202	21,578,439
RM5495-1	P	K	7	76.7-78.9	21,999,223	21,999,470
RM6152	P	K	7	78.9	22,074,885	22,075,111
RM6403	p	K	7	78.9	22,117,990	22,118,213
RM2752	P	K	7	78.9-80.5	22,498,355	22,498,624
RM6885	P	K	7	81.1	22,955,547	22,955,770
RM6432	P	K	7	81.4	23,107,080	23,107,309
RM3753-1	P	K	7	81.9	23,607,562	23,607,795
RM1365	P	K	7	81.9-83.3	23,737,026	23,737,277
RM6042	p	K	7	81.9-83.3	23,872,325	23,872,551
RM5397	P	K	7	81.9-83.3	23,899,674	23,899,901
RM1132	P	K	7	83.3	23,931,837	23,932,060
RM6326	P	K	7	83.3-84.1	24,142,506	24,142,759
RM6420	p	K	7	88.7	24,735,086	24,735,309
RM6344	p	K	7	89.8	24,910,592	24,910,815
RM1330-1	P	K	7	94.7	25,603,900	25,604,139
RM5426	P	K	7	96.1	25,877,964	25,878,195
RM7054	p	K	7	98.2	26,300,919	26,301,142
RM6216	P	K	7	98.2	26,343,822	26,344,048
RM5455	P	K	7	99.3	26,408,285	26,408,520
RM3555	P	K	7	105.7	27,837,942	27,838,165
RM5720	P	K	7	115.5	28,616,399	28,616,700
RM7601	p	K	7	116.6	28,985,910	28,986,137
RM6357-1	P	K	7	118.6	29,495,046	29,495,276
RM5911	P	K	8	0	69,028	69,293
RM6369	P	K	8	0	119,238	119,479
RM2680	P	K	8	0.5	141,310	141,573
RM1019	P	K	8	0.5	195,955	196,180
RM1959	P	K	8	1.6	259,219	259,456
RM1381-1	P	K	8	3	417,847	418,108
RM6925	P	K	8	3.6	641,650	641,939
RM1235-1	P	K	8	13.1	1,203,331	1,203,550
RM6356	P	K	8	13.4	1,555,391	1,555,620
RM6863	P	K	8	16.4	2,005,890	2,006,116
RM8018	P	K	8	16.7		
RM5647	P	K	8	21.6-25.2	2,886,460	2,886,592
RM4955	P	K	8	25.2	2,953,386	2,953,645
RM3819	P	K	8	25.2	3,009,695	3,009,934
RM1270-1	P	K	8	25.2-26.3	3,075,093	3,075,324
RM5434	P	K	8	25.2	3,118,660	3,118,891
RM1376	p	K	8	26.3	3,162,515	3,162,776
RM5428	p	K	8	27.1	3,246,672	3,246,903
RM1148	P	K	8	31.6	3,733,709	3,733,932
RM3572	p	K	8	32.7	3,921,948	3,922,171
RM6999	P	K	8	32.7	3,978,833	3,979,077
RM5432	p	K	8	34.6-35.7	4,371,964	4,372,195
RM4085	p	K	8	35.7	4,443,863	4,444,088
RM3791	P	K	8	35.7	4,510,395	4,510,632
RM5556	P	K	8	36	4,583,090	4,583,319
RM3374	P	K	8	36.8	4,725,654	4,725,886
RM1111	P	K	8	36.8	4,767,350	4,767,573
RM5808	p	K	8	36.8		
RM7080	p	K	8	36.8	4,694,512	4,694,735
RM6208	p	K	8	40.2-42.9	5,782,632	5,782,855
RM6838	P	K	8	42.9	5,844,143	5,844,384
RM3507	P	K	8	44.6-45.4	6,152,837	6,153,088
RM3644	p	K	8	44.6-45.4	6,199,529	6,199,756
RM2584	P	K	8	45.4-45.8		
RM3181	p	K	8	45.4-45.8	7,547,366	7,547,587
RM8019	P	K	8	45.84		
RM6429	p	K	8	48.8-49.2	8,379,004	8,379,230
RM3835	P	K	8	50.5	8,573,657	8,573,900
RM3481	P	K	8	50.8-52.1	9,129,774	9,130,017
RM6010	P	K	8		9,478,523	9,478,746
RM3395	P	K	8	52.9	10,288,396	10,288,629
RM5514	P	K	8	52.9-53.7	10,401,871	10,402,094
RM3662	P	K	8	54.0-54.3	11,721,092	11,721,319
RM1384-1	P	K	8	54.0-54.3	11,842,578	11,842,849
RM6032	P	K	8	54.0-54.3	11,890,094	11,890,317
RM3383	p	K	8	54.3	13,478,659	13,478,890
RM6990	P	K	8	54.3	14,355,543	14,355,766
RM6382	p	K	8	58.7-59.0	16,911,930	16,912,153
RM7285	P	K	8	61.2-66.5	18,756,284	18,756,543
RM5767	P	K	8	61.2-66.5	18,814,305	18,814,531
RM3153-1	P	K	8	66.5	18,982,379	18,982,628
RM4815	P	K	8	66.5	19,001,947	19,002,202
RM6215	P	K	8	66.5	19,058,482	19,058,708
RM7191	P	K	8	67.3-70.1	19,598,375	19,598,602
RM6187-1	P	K	8	72.2	20,474,153	20,474,376
RM3459	P	K	8	75.7	20,645,513	20,645,752
RM5351-1	P	K	8	76.7	20,825,098	20,825,323
RM1578-1	P	K	8	76.7-78.5	20,966,809	20,966,937
RM5887-1	P	K	8	76.7-78.5	20,971,607	20,971,836
RM7356	P	K	8	78.5	21,275,442	21,275,665
RM3634	P	K	8	80.7-82.8	21,575,792	21,576,017
RM6635	P	K	8	85.1	22,166,005	22,166,234
RM7556	P	K	8	86.7	22,202,467	22,202,694
RM3262	p	K	8	86.7	22,373,934	22,374,159
RM6976	P	K	8	92.2	23,551,098	23,551,342
RM5485	P	K	8	92.2-96.6	24,065,608	24,065,851
RM8058	P	K	8	99.1		
RM3845	p	K	8	99.1-102.1	24,755,418	24,755,663

RM4487	P	K	8	107.7	26,080,816	26,081,055
RM1345	P	K	8	107.7	26,140,324	26,140,569
RM5493-1	P	K	8	107.7	26,140,889	26,141,136
RM3571	P	K	8	108.2	26,243,549	26,243,772
RM7631	p	K	8	114.4-118.9	27,349,912	27,350,135
RM5933	p	K	8	114.4-118.9	27,465,770	27,466,113
RM6845	P	K	8	118.9	27,555,590	27,555,813
RM3120	P	K	8	119.6	27,812,944	27,813,167
RM3496-1	P	K	8	119.6	27,834,785	27,835,034
RM3155-1	P	-	8	119.9	27,898,003	27,898,264
RM3840	P	K	8	119.9	27,919,217	27,919,462
RM4153	P	K	8	120.4	28,196,203	28,196,440
RM4997	P	K	8	121.2	28,231,273	28,231,536
RM4154	P	K	8	121.2	28,238,630	28,238,857
RM5545	P	K	8	121.2	28,267,514	28,267,742
RM5688	P	K	9	0.8	1,663,450	1,663,700
RM3502	p	K	9	2.1-2.4	4,975,086	4,975,336
RM5899	P	K	9	2.1-2.4	5,069,962	5,070,209
RM6920	P	K	9	10	6,955,519	6,955,799
RM5515	p	K	9	10	7,093,619	7,093,842
RM1328	P	K	9	26.7	9,152,193	9,152,432
RM3855	P	K	9	26.7-30.6	9,316,925	9,317,174
RM3907	p	K	9	34.4	10,440,718	10,440,957
RM3769	P	K	9	36	11,694,673	11,694,908
RM1896	P	K	9	36	11,714,708	11,714,827
RM6444	p	K	9	36.0-40.1	11,826,987	11,827,210
RM6051	P	K	9	41.9	12,777,595	12,777,824
RM6475	p	K	9	41.9	12,786,082	12,786,308
RM6839	P	K	9	50.7	14,512,300	14,512,550
RM6771	p	K	9	50.7	14,638,336	14,638,559
RM5652	P	K	9	50.7	14,731,182	14,731,447
RM5122	P	?	9	55.3	15,194,221	15,194,492
RM3700	P	K	9	55.3	15,373,551	15,373,780
RM3025	P	K	9	58.3-60.8	16,332,095	16,332,378
RM3492	P	K	9	58.3-60.8	16,440,786	16,441,033
RM7424	p	K	9	60.8	16,527,050	16,527,273
RM6235	P	K	9	60.8-62.4	16,620,710	16,620,935
RM7048	P	K	9	62.4	16,881,548	16,881,779
RM3600	p	K	9	62.7	17,054,042	17,054,267
RM7289	P	K	9	63	17,210,136	17,210,427
RM5574	P	K	9	63.0-65.1	17,668,026	17,668,265
RM3533	P	K	9	65.1	17,833,742	17,833,965
RM5535	P	K	9	74.7	18,935,645	18,935,870
RM6460	P	K	9	74.7	18,978,882	18,979,105
RM7343	p	K	9	75	19,225,970	19,226,193
RM3249	p	K	9	77.2	19,453,932	19,454,157
RM6491	p	K	9	77.2	19,454,547	19,454,770
RM5786-1	P	K	9	77.2	19,493,602	19,493,870
RM3787	P	K	9	77.7-78.0	19,748,455	19,748,692
RM3808	p	K	9	78.8	20,246,418	20,246,657
RM1553	P	K	9	82.1	20,702,481	20,702,706
RM6971	P	K	9	90.4	21,591,726	21,591,964
RM5384-1	P	K	9	90.7-91.5	21,766,885	21,767,112
RM2915	p	K	9	90.7-91.5	21,784,272	21,784,549
RM6816	p	K	9	91.8	21,907,085	21,907,311
RM2144	P	K	9	91.8	21,908,523	21,908,768
RM6294	P	K	9	91.8	21,983,121	21,983,344
RM2482	P	K	9	93.1	22,275,100	22,275,347
RM1026	p	K	9	93.1	22,304,231	22,304,458
RM6797	p	K	9	93.5	22,412,038	22,412,261
RM3744	P	K	9	93.5	22,419,832	22,419,964
RM5095	P	K	10	0.6	31,772	32,039
RM7492	P	K	10	0.6	33,871	34,098
RM6370	P	K	10	1.1	324,345	324,586
RM3590	p	K	10	1.9-2.2	1,075,995	1,076,218
RM7361	P	K	10	3	1,901,703	1,901,930
RM5271	P	K	10	3.9	1,915,539	1,915,828
RM6404	P	K	10	4.4-5.5	2,415,410	2,415,633
RM3882	P	K	10	5.5-6.8	2,717,427	2,717,650
RM7545	P	K	10	7.6	3,785,015	3,785,286
RM6565	P	K	10	11.7-13.3	4,616,853	4,617,076
RM2504	P	K	10	15.7	7,217,413	7,217,668
RM2887	P	K	10	16.4	8,513,956	8,514,231
RM1126	P	K	10	16.8-17.6	9,294,322	9,294,545
RM3152-1	P	K	10	17.9	9,766,047	9,766,215
RM6833	P	K	10	19.0-21.8	10,893,395	10,893,630
RM4455	P	K	10	21.8-23.1	11,221,443	11,221,682
RM1859	P	K	10	24.7-26.1	13,041,096	13,041,230
RM6144	p	K	10		15,158,952	15,159,178
RM1375	P	K	10	??	16,201,987	16,202,248
RM3229	P	K	10	42.7-44.0	16,248,788	16,249,011
RM6477	p	K	10	42.7-44.0	16,291,023	16,291,249
RM3470	P	K	10	44.6-45.7	16,842,586	16,842,827
RM5392	P	K	10	44.6-45.7	16,859,910	16,860,137
RM5620	P	K	10	45.7	16,959,830	16,960,059
RM1873	P	K	10	48.4	17,379,779	17,380,014
RM5274	P	K	10	48.4	17,403,868	17,404,002
RM1937	P	K	10	48.4	17,483,380	17,483,617
RM6474	P	K	10	53.3	18,183,508	18,183,734
RM5629	P	K	10	53.3-53.6	18,230,357	18,230,589
RM6737	P	K	10	53.3-53.6	18,268,289	18,268,551
RM3510	P	K	10	54.3	18,633,958	18,634,211
RM1108	P	K	10	55.3	18,716,263	18,716,486
RM6745-1	P	K	10	55.6	18,897,227	18,897,405
RM2371	P	K	10	55.6-57.5	19,132,837	19,133,086
RM1146	P	K	10	55.6-57.5	19,168,982	19,169,205
RM5841	P	K	10	55.6-57.5	19,202,360	19,202,616
RM6691	P	K	10	61.4	19,785,088	19,785,371
RM4477	P	K	10	71.4	20,655,592	20,655,732
RM3451	P	K	10	72.8	21,122,835	21,123,072
RM3123	p	K	10	73.7	21,316,923	21,317,146
RM5471-1	p	K	10	73.7-83.0	21,403,092	21,403,331
RM5768	p	K	10	73.7-83.0	22,081,651	22,081,877

RM6614	p	K	10	73.7-83.0	22,302,371	22,302,597
RM6673	P	K	10	83.8	22,561,827	22,562,056
RM4771	P	K	10	83.8	22,564,979	22,565,230
RM7453	P	K	11	2.8-4.1	933,199	933,438
RM7203	P	K	11	4.1	1,083,833	1,084,064
RM3717	P	K	11	4.8	1,174,057	1,174,282
RM3668	P	-	11	6.5	1,463,300	1,463,527
RM2459	P	K	11	10.3	2,391,067	2,391,196
RM1812	P	K	11	10.3	2,391,986	2,392,217
RM5599-1	P	K	11	19.8	3,808,835	3,809,067
RM6544	p	K	11	19.85	3,838,097	3,838,323
RM3339	P	K	11	20.3	4,120,670	4,120,899
RM5704-1	P	K	11	28.6	5,403,551	5,403,810
RM3133	P	K	11	32.1-33.4	6,109,417	6,109,644
RM3137	P	K	11	32.1-33.4	6,110,975	6,111,204
RM4469	P	K	11	32.1-33.4	6,181,989	6,182,128
RM7248	P	K	11	45.3	7,930,320	7,930,543
RM3701	P	K	11	45.3	8,024,626	8,024,855
RM7283	P	K	11	49.6	9,024,117	9,024,368
RM3185	P	K	11	49.6-52.9	9,128,505	9,128,728
RM1206	P	K	11	52.9-54.3	9,668,428	9,668,655
RM4862	P	K	11	54.3	9,884,869	9,885,124
RM7463	P	K	11	54.8-55.1	10,095,186	10,095,425
RM7120	P	K	11	55.9-56.2	11,684,902	11,685,125
RM5857	P	K	11	55.9-56.2	11,759,816	11,760,108
RM6091	p	K	11	56.2	13,290,044	13,290,276
RM3428	P	K	11	56.2	13,366,516	13,366,751
RM5824	P	K	11	56.2-57.3	13,950,019	13,950,254
RM6272	P	K	11	64.2	16,400,544	16,400,773
RM4746	P	K	11	64.2	16,455,330	16,455,565
RM1341	P	K	11	80.2-80.5	19,438,976	19,439,219
RM6422	p	K	11	80.2-80.5	19,483,244	19,483,464
RM6534	P	K	11	84.6-85.7	20,662,189	20,662,415
RM4601	P	K	11	90.1	22,251,890	22,252,012
RM6499	P	K	11	91.4-97.3	23,227,875	23,228,098
RM4112	P	K	11	101.9	24,257,229	24,257,456
RM2191-1	P	K	11	101.9	24,271,958	24,272,201
RM1233-1	P	K	11	112.9	26,137,367	26,137,596
RM2064	P	K	11	114.4-115.1	26,612,971	26,613,111
RM2136	P	K	11	117.9	28,302,443	28,302,680
RM7443	P	-	11	117.9	28,322,869	28,323,096
RM5568	P	K	12	9.4-9.7	711,799	712,034
RM1880	P	K	12	9.4-9.7	746,907	747,142
RM1080	P	K	12	9.7	905,086	905,331
RM3323	P	K	12	9.7	975,922	976,149
RM1208	P	K	12	9.7	1,074,283	1,074,510
RM7315	P	K	12	12.2-26.0	2,181,945	2,182,180
RM3747	P	K	12	12.2-26.0	2,304,382	2,304,615
RM6998	P	K	12	30.0-38.1	4,745,116	4,745,351
RM7619	P	K	12	38.1	4,828,787	4,829,038
RM5746	p	K	12	39.4	5,091,932	5,092,155
RM7119	P	K	12	41.2	6,694,643	6,694,866
RM2935	P	K	12	47	7,424,904	7,425,181
RM2529	P	K	12	47.6	7,566,687	7,566,944
RM1036	P	K	12	48.2	8,796,058	8,796,285
RM7195	P	K	12	49.3	9,895,547	9,895,774
RM6973-1	P	K	12	49.3	10,080,086	10,080,324
RM5364	P	K	12	51.5	12,025,668	12,025,893
RM7344	p	K	12	62.2	17,990,727	17,990,950
RM1246-1	P	K	12	65.3	19,156,050	19,156,279
RM5700-1	P	K	12	73.3-75.8	22,016,809	22,017,068
RM6869	P	K	12	75.8	22,286,778	22,287,001
RM1015	P	K	12	75.8-78.9	22,473,762	22,473,987
RM3813	P	K	12	86.5-88.6	23,313,519	23,313,760
RM7376	P	K	12	88.6-91.4	23,510,599	23,510,822
RM3331	P	K	12	88.6-91.4	23,527,988	23,528,217
RM1103	P	K	12	91.4	23,606,675	23,606,898
RM6693	p	K	12	91.9	23,832,690	23,832,979
RM6947	p	K	12	91.9-94.6	24,041,241	24,041,464
RM5479-1	P	K	12	95.1-95.4	24,446,105	24,446,346
RM5715-1	P	K	12	97	25,067,657	25,067,940
RM7025	p	K	12	100.9-103.1	25,923,135	25,923,358
RM1194	P	K	12	100.9-103.1	25,931,697	25,931,924
RM1310	P	K	12	100.9-103.1	25,940,358	25,940,595
RM1300	P	K	12	100.9-103.1	26,032,495	26,032,730
RM6189	p	K	12	100.9-103.1	26,055,257	26,055,480
RM1999	P	K	12	103.1	26,182,327	26,182,565
RM2734	P	K	12	103.1	26,220,916	26,221,181
RM1226	P	K	12	109.2	27,380,115	27,380,344
RM2197	P	K	12	109.2	27,420,384	27,420,629

*RAP Build3
