# Supplementary material for: Natural Variation of the RICE FLOWERING LOCUS T 1 Contributes to Flowering Time Divergence in Rice
Source: PLoS One. 2013 Oct 1;8(10):e75959. doi: 10.1371/journal.pone.0075959 (PMC3788028; doi:10.1371/journal.pone.0075959)
Supplement: Table S2 — Information for the 24 cultivars used in Figure S5. (PDF) [file pone.0075959.s013.pdf]

Table S2: Information for 24 cultivars, the flowering time and the expression levels of *RFT1* and *Hd3a* under SD, LD and ND conditions.

Name of accessions	Categoty	SD			LD			ND		
		**RFT*	**Hd3a*	Days to heading	**RFT*	**Hd3a*	Days to heading	**RFT*	**Hd3a*	Days to heading
Miryang23	indica	2.4E-04	5.8E-04	64	4.7E-05	1.76E-06	98	9.7E-05	3.0E-04	107
IR24	indica	1.0E-05	1.5E-04	86	1.0E-04	2.84E-06	103	1.2E-04	5.3E-06	116
DV85	indica	2.3E-06	4.8E-04	78	1.1E-05	1.06E-06	87	5.9E-04	4.3E-06	97
ARC10313	indica	1.9E-06	5.1E-04	68	2.2E-03	4.26E-05	68	6.1E-03	8.5E-04	85
Bhadua	indica	7.4E-06	3.4E-03	51	4.2E-06	2.66E-06	146	1.0E-05	2.2E-06	136
Taichung65	indica	8.5E-05	1.5E-04	94	9.1E-06	3.29E-06	119	2.6E-04	5.6E-06	107
Nona Bokra	indica	1.1E-03	4.3E-03	50	4.4E-06	2.1E-06	160	3.6E-06	9.5E-07	209
Kasalath	indica	1.1E-06	2.3E-04	80	9.8E-05	3.45E-06	88	3.9E-04	3.5E-06	101
Kamenoo	japonica	2.1E-04	2.2E-03	50	1.5E-05	1.6E-06	107	1.9E-04	2.3E-06	114
Kameji	japonica	2.4E-04	5.4E-03	50	9.7E-06	2.47E-06	133	9.6E-05	1.2E-06	122
Takenari	japonica	4.4E-04	9.1E-03	48	1.4E-05	1.2E-06	157	9.5E-05	2.2E-06	119
Jinriki	japonica	7.7E-05	1.3E-03	53	4.4E-06	1.5E-06	169	3.4E-05	7.3E-07	126
Ooba	japonica	7.6E-05	6.2E-04	53	1.0E-04	5.63E-06	94	9.3E-04	2.4E-05	100
Asahi	japonica	1.1E-04	6.2E-03	52	6.2E-06	1.65E-06	160	2.4E-05	2.9E-07	123
Ginbozu	japonica	7.5E-05	4.0E-04	55	3.3E-05	3.51E-06	112	9.5E-05	6.8E-07	122
Asominori	japonica	4.5E-04	6.5E-03	50	1.1E-05	2.26E-06	118	9.1E-05	1.3E-06	115
Nipponbare	japonica	8.9E-04	8.3E-03	48	1.3E-05	1.52E-06	116	2.2E-04	1.7E-05	114
Koshihikari	japonica	2.0E-04	1.3E-03	53	4.1E-03	0.000954	76	6.6E-04	3.4E-05	99
Sasanishiki	japonica	2.1E-05	3.6E-05	60	4.6E-04	1.42E-05	81	1.8E-03	2.8E-05	95
Dontokoi	japonica	6.9E-05	2.3E-04	59	9.6E-04	6.62E-05	80	3.8E-04	2.1E-05	104
Akihikari	japonica	9.8E-05	6.0E-04	57	3.1E-03	0.000138	70	4.7E-03	4.4E-04	89
Habataki	japonica	1.8E-04	3.1E-04	62	1.4E-03	9.28E-06	79	7.2E-04	2.9E-05	99
Hayamasari	japonica	1.3E-04	8.3E-04	54	4.3E-03	0.018787	53	9.3E-03	1.6E-02	62
Hoshinoyume	japonica	4.5E-04	3.8E-03	51	4.8E-03	0.008008	58	4.5E-03	8.9E-03	75

*Relative expression levels
