# Supplementary material for: Natural Variation of the RICE FLOWERING LOCUS T 1 Contributes to Flowering Time Divergence in Rice
Source: PLoS One. 2013 Oct 1;8(10):e75959. doi: 10.1371/journal.pone.0075959 (PMC3788028; doi:10.1371/journal.pone.0075959)
Supplement: Table S3 — Information for 204 tested rice accessions. (PDF) [file pone.0075959.s014.pdf]

Table S3: Information for 204 tested rice accessions

No.	No.	Accession	*RFT1* pro	*RFT1* cds	*RFT1* Entire	*Hd3a* pro	*Hd3a* cds	*Hd3a* Entire	Hd1 function [19]	Hd1 Allele group [24]	Category	Local origin	Varietiy Name	Flowering time SD	Flowering time LD	Flowering time ND
137	1	WRC001	1	1	I	2	2	I	F	I	Japonica	Japan	Nipponbare	94	116	46
138	2	WRC002	4	4	IV	4		IX	N	IV	Indica	India	Kasalath	86	77	109
139	3	WRC003	2	2	II	16		III	F	I	Indica	Cambodia	Bei Khe	73	90	125
140	4	WRC004	3	3	III	3	1	III	N	II	Indica	Nepal	Jene 035	75	80	108
141	5	WRC005	4	4	IV	4	1	IV	N	II	Indica	India	Naba	74	123	127
142	6	WRC006	2	4	7	1	1	II	N	II	Indica	Indonesia	Puluik Arang			137
143	7	WRC007	2	2	II	14	1		N	I	Indica	Philippines	Davao 1	97	85	111
144	8	WRC009	1	1	I	2	14	X	F	I	Indica	China	Ryou Suisan Koumai	51	58	94
145	9	WRC010	1	1	I	2	2	I	N		Indica	China	Qiu Zhao Zhong	70	65	91
146	10	WRC011		4		4	1	IV	N	II	Indica	China	Jinguoyin	81	88	114
147	11	WRC013	2	2	II	1	1	II	N	II	Indica	Bhutan	Asu	66	108	120
148	12	WRC014	1	1	I	2	2	I	N	II	Indica	Philippines	IR 58	64	82	109
149	13	WRC015	4	4	IV	4	1	IV	F	I	Indica	India	Co 13	68	112	116
150	14	WRC016	2	2	II	1	1	II	N		Indica	Madagascar	Vary Futsi	93	106	111
151	15	WRC017	2	2	II	14	1		N	II	Indica	China	Keiboba	78	71	103
152	16	WRC018	2	2	II				N	I	Indica	Taiwan	Qingyu(Seiyu)	88	85	108
153	17	WRC019	4	4	IV	4	1	IV	F	I	Indica	China	Deng Pao Zhai	62	100	122
154	18	WRC020	5	2	9	1	1	II	N		Indica	Philippines	Tadukan	98	126	125
155	19	WRC021	1	1	I	2	4	VIII	N	I	Indica	Myanmar	Shwe Nang Gyi	85	93	105
156	20	WRC022	6	1	10	2	2	I	N	I	Indica	Philippines	Calotoc	103	95	116
157	21	WRC023	1	1	I	4	2		N	II	Indica	Philippines	Lebed			
158	22	WRC024	1	1	I	2	2	I	N	I	Indica	Philippines	Pinulupot 1	82	101	115
159	23	WRC025	4	4	IV	4	1	IV	N	IV	Indica	India	Muha	75	94	106
160	24	WRC026	4	4	IV	4	1	IV	N	III	Indica	India	Jhona 2			
161	25	WRC027	3	3	III	4	1	IV	N	II	Indica	Nepal	Napal 8	56	74	103
162	26	WRC028	3	3	III	3	1	III	N	II	Indica	Bhutan	Jarjan	73	90	111
163	27	WRC029	4	4	IV	4	1	IV	N	IV	Indica	Nepal	Kalo Dhan	74	89	89
164	28	WRC030	3	3	III	4	1	IV	N	IV	Indica	Nepal	Anjana Dhan	56	86	114
165	29	WRC031	7	4	11	4	1	IV	N	IV	Indica	Bangladesh	Shoni	71	69	95
166	30	WRC032	4	4	IV	4	1	IV	N	II	Indica	Bangladesh	Tupa 121-3	77	87	102
167	31	WRC033	4	4	IV	4	1	IV	N	IV	Indica	India	Surjamukhi	61	86	105
168	32	WRC034	4	4	IV	4	1	IV	N	IV	Indica	India	ARC 7291	73	82	106
169	33	WRC035	4	4	IV	4	1	IV	N	II	Indica	India	ARC 5955	86	99	108
170	34	WRC036	8	3	5	3	1	III	N	IV	Indica	India	Ratul	68	77	103
171	35	WRC037	4	4	IV	4	1	IV	N	IV	Indica	India	ARC 7047	54	73	96
172	36	WRC038	4	4	IV	4	1	IV	N	IV	Indica	India	ARC 11094	66	64	105
173	37	WRC039	3	3	III	3	1	III	F	III	Indica	Nepal	Badari Dhan	43	80	107
174	38	WRC040	3	3	III	3	1	III	N	IV	Indica	India	Nepal 555	64	91	109
175	39	WRC042				2	2	I	N	IV	Indica	India	Local Basmati	85	127	126
176	40	WRC043	1	1	I	2	2	I	N	I	Japonica	China	Dianyu 1	68	104	74
177	41	WRC044	1	1	I	2	2	I	N	I	Indica	Philippines	Basilanon	112	106	115
178	42	WRC045	1	1	I	2	2	I	N	II	Japonica	Myanmar	Ma Sho			
179	43	WRC046	1	5	4	2	2	I	N	II	Japonica	Laos	Khao Nok			
180	44	WRC047	1	1	I	2	2	I	N	II	Japonica	Brazil	Jaguary	81	110	60
181	45	WRC048	1	5	4	2	15	XI	N	II	Japonica	Vietnam	Khau Mac Kho	95	124	88
182	46	WRC049	1	1	I	2	2	I	N	II	Japonica	Indonesia	Padi Perak	116	126	118
183	47	WRC050	4	4	IV	4	1	IV	N	II	Japonica	United States	Rexmont	65	108	60
184	48	WRC051	1	1	I	2	2	I	F	II	Japonica	Japan	Urasan 1	74	98	74
185	49	WRC052				2	2	I	F	II	Japonica	Vietnam	Khau Tan Chiem	98	117	109
186	50	WRC053	1	1	I	2	5	V	F	II	Japonica	Bhutan	Tima	106	129	60
187	51	WRC055	1	1	I	2	5	V	N	IV	Japonica	Bangladesh	Tupa 729	85	99	83
188	52	WRC057	1	1	I	2	4	VIII	N	I	Indica	Korea	Milyang 23	74	87	110
189	53	WRC058	2	2	II	5	3	VI	F	I	Indica	Cambodia	Neang Menh	85	>150	>200
190	54	WRC059	2	2	II	1	1	II	F	I	Indica	Cambodia	Neang Phtong			>200
191	55	WRC060	2	2	II	1	1	II	F		Indica	Laos	Hakphaynhay	72	>150	>200
192	56	WRC061	2	4	7	1	1	II	F	I	Indica	Malaysia	Radin Goi Sesat	86	>150	>200
193	57	WRC062	2	2	II	1	1	II	F	II	Indica	Malaysia	Kemasin	81	>150	>200
194	58	WRC063	2	2	II	1	1	II	F	I	Indica	Thailand	Bleiyo	36	>150	191
195	59	WRC064	2	2	II	1	2	XII	N	II	Indica	Indonesia	Padi Kuning			
196	60	WRC065	2	2	II	1	1	II	F	I	Indica	Indonesia	Rambhog			>200
197	61	WRC066	4	4	IV	4	1	IV	F		Indica	Myanmar	Bingala			>200
198	62	WRC067	1	1	I	2	2	I	F	II	Japonica	India	Phulba	132	>200	45
199	63	WRC068	1	1	I	2	2	I		II	Japonica	Laos	Khao Nam Jen	131	186.2	52
200	64	WRC096	2	2	II	1	1	II	F		Indica	India	POKALLI			>200
201	65	WRC097	1	5	4	6	2	XIII			Indica	Myanmar	Chin Galay			>200
202	66	WRC098	2	2	II		1				Indica	China	Deejiaohualuo	95	84	104
203	67	WRC099	9	2	12		1				Indica	China	Hong Cheuh Zai	72	82	102
204	68	WRC100	2	2	II	1	1	II	N		Indica	Sri Lanka	Vandaran	55	86	117
118	69	Nona Bokra	2	2	II	23	1		F		Indica	India	NONA BOKRA	52	>200	>200
3	70	IRGC000237	2	2	II	1	1	II	N		Indica	India	TKM6			
4	71	IRGC001107	1	1	I	2	2	I			Japonica	China	Ta Hung Ku			
5	72	IRGC006046	2	2	II	1	1	II			Indica	Bangladesh	DA11			
6	73	IRGC006570	3	3	III	3	1	III			Indica	Bangladesh	Aswina 322			
7	74	IRGC006590				3	1	III			Indica	Bangladesh	Baguamon 14			
8	75	IRGC008177	2	2	II	1	1	II	F		Indica	Vietnam	RTS4			
9	76	IRGC008195	4	4	IV	4	1	IV			Indica	China	Kun-Min-Tsieh-Huang			
10	77	IRGC008231	2	2	II	1	1	II			Indica	Vietnam	GIE 57			
11	78	IRGC008234	2	2	II	1	1	II			Indica	Vietnam	RTS12			
12	79	IRGC008242				4	1	IV			Indica	Taiwan	Shuang-Chiang			
13	80	IRGC008260	1	1	I	2	2	I			Indica	Indonesia	Seratoes Hari			
14	81	IRGC008264	1	1	I	2	2	I			Japonica	China	Hu-Lo-Tao			
15	82	IRGC009069	8	3	5		1				Indica	India	JC148			
16	83	IRGC009177	2	2	II	1	1	II			Indica	India	JC91			
17	84	IRGC009967	2	2	II	1	1	II			Indica	India	Deep Water Paddy			
18	85	IRGC011043	2	2	II	1	1	II			Indica	India	BR8			

19	86	IRGC012883	1	5	4	2	2	I		Japonica	Iran	Mehr
20	87	IRGC017757	1	5	4	2	2	I		Japonica	Indonesia	Jambu
21	88	IRGC024224	2	2	II	5	3	VI		Indica	Thailand	Khao Gaew
22	89	IRGC026514				1	3	XIV		Indica	Bangladesh	Nara Aswina
23	90	IRGC027513	3	3	III	3	1	III		Japonica	Bangladesh	Dholi Boro
24	91	IRGC027588					2			Indica	Bangladesh	Rayada
25	92	IRGC027748	2	2	II	1	1	II		Indica	Thailand	Khao Dawk Mail 105
26	93	IRGC030238	2	2	II	1	1	II		Indica	Thailand	Champa Tong 54
27	94	IRGC030416	2	2	II		1			Indica	Philippines	IR36
28	95	IRGC031696	3	3	III	3	1	III		Indica	Bangladesh	Aswina
29	96	IRGC032362	4	4	IV	4	1	IV		Japonica	Iran	Tchampa
30	97	IRGC032399	1	1	I	2	5	V		Indica	Bhutan	Phudugey
31	98	IRGC038994	1	1	I	2	2	I		Japonica	Brazil	Bico Branco
32	99	IRGC042469	1	5	4	2	2	I		Japonica	India	ARC 13829
33	100	IRGC043369	2	2	II	1		II		Indica	Indonesia	Cere Air
34	101	IRGC043400	2	2	II	1		II		Indica	Indonesia	Ilis Air
35	102	IRGC043675	10	1	6	2	2	I		Japonica	Indonesia	Tremnese
36	103	IRGC051250	1	1	I	2	2	I		Indica	China	Ai-Chiao-Hong
37	104	IRGC051300	2	4	13	1	1	II	F	Indica	China	Guan-Yin-Tsan
38	105	IRGC053637	1	5	4	2	2	I		Japonica	India	Basmati 217
39	106	IRGC056036	2	2	II	1	1	II		Indica	Vietnam	Chau
40	107	IRGC064801	2	2	II	1	1	II		Indica	Bangladesh	BR34
41	108	IRGC077210	3	3	III	3	3	III		Indica	Bangladesh	Rayada
42	109	IRGC077483	2	2	II		1			Indica	Brazil	IRGA 409
68	110	JRC001	1	1	I	2	2	I		Japonica	Japan	GAISEN MOCHI
69	111	JRC003	1	1	I	2	2	I		Japonica	Japan	HINODE
70	112	JRC004	10	1	6	2	2	I		Japonica	Japan	SENSHOU
71	113	JRC005	1	1	I	2	2	I		Japonica	Japan	YAMADA BAKE
72	114	JRC006	1	1	I	2	2	I		Japonica	Japan	KANEKO
73	115	JRC007	10	1	6	2	2	I		Japonica	Japan	IRIMA NISHIKI
74	116	JRC008	1	1	I	2	2	I		Japonica	Japan	OKKA MODOSHI
75	117	JRC010	1	1	I	2	10	I		Japonica	Japan	HIRAYAMA
76	118	JRC011	1	1	I	2	2	I		Japonica	Japan	KAHEI
77	119	JRC012	1	1	I	2	2	I		Japonica	Japan	OIRAN
78	120	JRC013	1	1	I	2	2	I		Japonica	Japan	BOUZU MOCHI
79	121	JRC014	1	1	I	2	2	I		Japonica	Japan	MEGURO MOCHI
80	122	JRC017	1	1	I	2		**VII**		Japonica	Japan	AKAGE
81	123	JRC018	1	1	I	2	2	I		Japonica	Japan	HASSOKUHO
82	124	JRC019	1	1	I	2	4	VIII		Japonica	Japan	WATARIBUNE
83	125	JRC020	1	1	I	2	2	I		Japonica	Japan	HOSOGARA
84	126	JRC021	2	2	8	1	1	II		Indica	Japan	AKAMAI
85	127	JRC022	1	1	I	2	2	I		Japonica	Japan	MANSAKU
86	128	JRC023	1	1	I	2	2	I		Japonica	Japan	ISHIJIRO
87	129	JRC024	1	1	I	2	2	I		Japonica	Japan	JOUSHUU
88	130	JRC025	1	1	I	2	2	I		Japonica	Japan	DANGO
89	131	JRC026	1	1	I	2	2	I		Japonica	Japan	AIKOKU
90	132	JRC027	1	1	I	2	2	I		Japonica	Japan	GINBOUZU
91	133	JRC028	1	1	I	2	4	VIII		Japonica	Japan	SHINRIKI MOCHI
92	134	JRC029	1	1	I	2	2	I		Japonica	Japan	SHICHIMENCHOU MOCHI
93	135	JRC030	1	1	I	2	2	I		Japonica	Japan	MORITA WASE
94	136	JRC031	1	1	I	2	2	I		Japonica	Japan	KAMEJI
95	137	JRC032	1	1	I	2	4	VIII		Japonica	Japan	OMACHI
96	138	JRC033	1	1	I	2	4	VIII		Japonica	Japan	SHINRIKI
97	139	JRC034	1	1	I	2	2	I		Japonica	Japan	KYOUTOASAHI
98	140	JRC035	1	1	I	2	2	I		Japonica	Japan	KABASHIKO
99	141	JRC036	1	1	I	2	2	I		Japonica	Japan	SEKIYAMA
100	142	JRC037	1	1	I	2	2	I		Japonica	Japan	SHINYAMADAHO 2
101	143	JRC038	1	1	I	2	10	**VII**		Japonica	Japan	NAGOYA SHIRO
102	144	JRC039	1	1	I	2	2	I		Japonica	Japan	SHIROINE(KEMOMI)
103	145	JRC040	1	1	I	2	2	I		Indica	Japan	AKAMAI
104	146	JRC041	1	1	I	2	2	I		Indica	Japan	AKAMAI
105	147	JRC042	2	2	II	1	1	II		Indica	Japan	TOUBOSHI
106	148	JRC043	2	2	II	1	1	II		Indica	Japan	AKAMAI
107	149	JRC044	2	4	7	1	1	II		Indica	Japan	KARAHOUSHI
108	150	JRC045	1	1	I	2	2	I		Japonica	Japan	HIYADACHITOU
109	151	JRC046	1	1	I	2	10	**VII**		Japonica	Japan	FUKOKU
110	152	JRC047	1	1	I	2	2	I		Japonica	Japan	OKABO
111	153	JRC048	1	1	I	2	2	I		Japonica	Japan	HAKAMURI(YOKOYAMA)
112	154	JRC049	1	1	I	2	2	I		Japonica	Japan	RIKUTOU RIKUU 2
113	155	JRC050	1	1	I	2	4	VIII		Japonica	Japan	HIMENOMOCHI
114	156	JRC051	1	1	I	2	2	I		Japonica	Japan	SHINSHUU
115	157	JRC052	1	1	I	2	2	I		Japonica	Japan	AICHIASAHI
116	158	JRC053	1	1	I	2	4	VIII		Japonica	Japan	RAIDEN
117	159	JRC054	1	1	I	4	2			Japonica	Japan	HOUMANSHINDEN IN LD
1	160	IRGC101508	25	6	14	3	19	XIX		O. rufipogon	India	
2	161	IRGC105908	26	8	15	11	7			O. rufipogon	Thailand	
43	162	IRGC082990					29			O. rufipogon	China	
44	163	IRGC082993		7		10	2			O. rufipogon	China	
45	164	IRGC101971	3	3	III	3	1	III		O. rufipogon	India	
46	165	IRGC102171	11	6	16	7	7	XV		O. rufipogon	India	
47	166	IRGC102179	12	6	17	7	7	XV		O. rufipogon	India	
48	167	IRGC103824	2	2	II		20	XXIII		O. rufipogon	China	W1723
49	168	IRGC103827	13	7	18		11	**XII**		O. rufipogon	Bangladesh	
50	169	IRGC104404	14	4	19	8	17	XVI		O. rufipogon	Thailand	
51	170	IRGC104626	15	1	20	9	8	XVII		O. rufipogon	China	
52	171	IRGC104639		4			21			O. rufipogon	Thailand	
53	172	IRGC104684	16	6	21	10	7	XIX		O. rufipogon	India	
54	173	IRGC104969	17	3	22	11	6	XVIII		O. rufipogon	China	
55	174	IRGC105375		1			22			O. rufipogon	Thailand	
56	175	AS017	3	3	III	3	1	III		O. rufipogon	Bangladesh	
57	176	AS051								O. rufipogon	Nepal	

58	177	AS062	3	3	III	3	1	III	O. rufipogon	Laos
59	178	AS065	27	6	23				O. rufipogon	Indonesia
60	179	AS068					23		O. rufipogon	Bangladesh
61	180	AS074					11		O. rufipogon	India
62	181	AS078					24		O. rufipogon	Cambodia
63	182	AS083					25		O. rufipogon	Vietnam
64	183	AS084	28	9	24	8	9	XVIII	O. rufipogon	China
65	184	AS088	29	1	25	13	16	XVII	O. rufipogon	China
66	185	AS089	30	3	26	15	6	XXII	O. rufipogon	Indonesia
67	186	AS092	31	10	27	9	18		O. rufipogon	Vietnam
120	187	W0106	36	8	26	12	27	XX	O. rufipogon	India
121	188	W0120				13	12	XXI	O. rufipogon	India
122	189	W0157	32	1	27	9	9	XVIII	O. rufipogon	India
119	190	W0574					1		O. rufipogon	
123	191	W0593	18	12	28				O. rufipogon	Malaysia
124	192	W0630					3		O. rufipogon	Myanmar
125	193	W1294				12	28		O. rufipogon	Philippines
126	194	W1666	33	11	29	14	12	XXII	O. rufipogon	India
127	195	W1715	19	13	30				O. rufipogon	China
128	196	W1807	20	3	31	15	13	XXIII	O. rufipogon	Sri Lanka
129	197	W1865	34	3	32	15	13	XXIII	O. rufipogon	Thailand
130	198	W1943	37	1	33	16	8	XXIV	O. rufipogon	China
131	199	W1944	21	1	34	16	8	XXV	O. rufipogon	China
132	200	W1981	22	6	35				O. rufipogon	Indonesia
133	201	W2003	23	6	36				O. rufipogon	India
134	202	W2014	35	1	37	16	8	XXVI	O. rufipogon	India
135	203	W2263					9	XVIII	O. rufipogon	Cambodia
136	204	W2265	24	10	38		26	XXI	O. rufipogon	Laos
