# Supplementary material for: Natural Variation of the RICE FLOWERING LOCUS T 1 Contributes to Flowering Time Divergence in Rice
Source: PLoS One. 2013 Oct 1;8(10):e75959. doi: 10.1371/journal.pone.0075959 (PMC3788028; doi:10.1371/journal.pone.0075959)
Supplement: Table S4 — Tajima’s relative rate test for Hd3a and RFT1 using BradHd3a ( Brachypodium distachyon ) DNA sequence as an out group. (PDF) [file pone.0075959.s015.pdf]

Table S4: Tajima's Relative Rate Test for *Hd3a* and *RFT1* with *BradHd3a* (*Brachypodium distachyon*) DNA sequences

Accession	Outgroup	Identical sites	Unique sites	Unique Sites			$\chi 2$	dN/dS	
				Hd3a	*RFT1*	Outgroup		*Hd3a*	*RFT1*
101508	Bradi	439	14	18	22	41	0.4000	0.1422	0.1508
105908	Bradi	441	11	17	18	47	0.0286	0.1561	0.1471
A001	Bradi	444	11	14	17	48	0.2903	0.1478	0.1471
A002	Bradi	443	13	16	19	43	0.2571	0.1305	0.1473
A006	Bradi	444	11	14	17	48	0.2903	0.1478	0.1471
A008	Bradi	444	11	14	17	48	0.2903	0.1478	0.1470
A011	Bradi	444	11	14	17	48	0.2903	0.1478	0.1471
A014	Bradi	445	11	14	16	48	0.1333	0.1478	0.1405
A015	Bradi	444	11	14	17	48	0.2903	0.1478	0.1471
A016	Bradi	444	11	14	17	48	0.2903	0.1478	0.1471
A018	Bradi	443	13	16	19	43	0.2571	0.1305	0.1473
A019	Bradi	443	13	16	19	43	0.2571	0.1305	0.1473
A020	Bradi	444	11	14	17	48	0.2903	0.1478	0.1470
A021	Bradi	444	11	14	17	48	0.2903	0.1478	0.1471
A022	Bradi	444	11	14	17	48	0.2903	0.1478	0.1471
A024	Bradi	444	11	14	17	48	0.2903	0.1478	0.1471
A025	Bradi	442	13	16	20	43	0.4444	0.1305	0.1540
A027	Bradi	442	13	16	20	43	0.4444	0.1305	0.1540
A028	Bradi	446	11	12	17	48	0.8621	0.1341	0.1471
A030	Bradi	446	11	12	17	48	0.8621	0.1341	0.1471
A032	Bradi	444	11	14	17	48	0.2903	0.1478	0.1470
A035	Bradi	444	11	14	17	48	0.2903	0.1478	0.1471
A036	Bradi	444	11	14	17	48	0.2903	0.1478	0.1471
A037	Bradi	444	11	14	17	48	0.2903	0.1478	0.1471
A039	Bradi	444	11	14	17	48	0.2903	0.1478	0.1470
A040	Bradi	445	11	14	16	48	0.1333	0.1478	0.1405
A041	Bradi	442	13	17	19	43	0.1111	0.1373	0.1473
A045	Bradi	443	13	16	19	43	0.2571	0.1305	0.1473
A047	Bradi	442	13	16	20	43	0.4444	0.1305	0.1540
A048	Bradi	444	11	14	17	48	0.2903	0.1478	0.1471
A049	Bradi	444	11	14	17	48	0.2903	0.1478	0.1471
A050	Bradi	443	13	16	19	43	0.2571	0.1305	0.1473
A053	Bradi	443	13	16	19	43	0.2571	0.1305	0.1473
A054	Bradi	445	11	14	16	48	0.1333	0.1478	0.1405
A055	Bradi	442	13	16	20	43	0.4444	0.1305	0.1540
A056	Bradi	444	11	14	17	48	0.2903	0.1478	0.1471
A059	Bradi	444	11	14	17	48	0.2903	0.1478	0.1471
A063	Bradi	446	11	12	17	48	0.8621	0.1341	0.1470
A064	Bradi	444	11	14	17	48	0.2903	0.1478	0.1471
A074	Bradi	443	13	16	19	43	0.2571	0.1305	0.1473
A082	Bradi	444	11	14	17	48	0.2903	0.1478	0.1470
A086	Bradi	439	14	18	22	41	0.4000	0.1422	0.1508
A087	Bradi	439	14	18	22	41	0.4000	0.1422	0.1508
A091	Bradi	445	10	13	17	49	0.5333	0.1408	0.1471
A092	Bradi	441	11	17	18	47	0.0286	0.1559	0.1471
A096	Bradi	442	10	17	17	48	0.0000	0.1561	0.1405
A100	Bradi	439	13	20	20	42	0.0000	0.1451	0.1473
A103	Bradi	441	10	18	17	48	0.0286	0.1628	0.1405
A107	Bradi	439	14	18	22	41	0.4000	0.1422	0.1508
A112	Bradi	444	11	14	16	49	0.1333	0.1438	0.1470
A114	Bradi	441	14	17	19	43	0.1111	0.1438	0.1473
AS017	Bradi	444	11	14	17	48	0.2903	0.1478	0.1470
AS062	Bradi	444	11	14	17	48	0.2903	0.1478	0.1470
AS083	Bradi	443	11	16	17	47	0.0303	0.1490	0.1303
AS084	Bradi	441	11	16	17	49	0.0303	0.1628	0.1535
AS088	Bradi	439	13	20	20	42	0.0000	0.1559	0.1473
AS089	Bradi	444	11	14	16	49	0.1333	0.1438	0.1470
AS092	Bradi	440	11	19	17	47	0.1111	0.1584	0.1405
JRC012	Bradi	443	13	16	19	43	0.2571	0.1305	0.1473
JRC021	Bradi	444	11	14	17	48	0.2903	0.1478	0.1471
JRC022	Bradi	443	13	16	19	43	0.2571	0.1305	0.1473
JRC027	Bradi	443	13	16	19	43	0.2571	0.1305	0.1473
JRC033	Bradi	442	13	17	19	43	0.1111	0.1270	0.1473
JRC034	Bradi	443	13	16	19	43	0.2571	0.1305	0.1473
JRC042	Bradi	444	11	14	17	48	0.2903	0.1478	0.1471
JRC047	Bradi	443	13	16	19	43	0.2571	0.1305	0.1473
NonaBokra	Bradi	444	11	14	17	48	0.2903	0.1478	0.1471
O_brachyantha	Bradi	433	12	18	21	50	0.2308	0.1473	0.1610
O_longistaminata	Bradi	439	12	19	22	42	0.2195	0.1524	0.1442
O_meridionalis	Bradi	438	9	18	23	46	0.6098	0.1422	0.1615
O_punctata	Bradi	434	8	18	25	49	1.1395	0.1248	0.1188
W0106	Bradi	443	11	15	17	48	0.1250	0.1542	0.1471

W0157	Bradi	438	13	21	20	42	0.0244	0.1628	0.1473
W1294	Bradi	437	13	19	23	42	0.3810	0.1384	0.1787
W1666	Bradi	441	12	17	18	46	0.0286	0.1561	0.1471
W1807	Bradi	443	11	15	18	47	0.2727	0.1422	0.1470
W1865	Bradi	443	11	15	18	47	0.2727	0.1422	0.1470
W1943	Bradi	439	13	20	20	42	0.0000	0.1451	0.1473
W1944	Bradi	439	13	20	20	42	0.0000	0.1451	0.1473
W2014	Bradi	439	13	20	20	42	0.0000	0.1451	0.1473
W2265	Bradi	442	11	17	17	47	0.0000	0.1561	0.1405
WRC001	Bradi	443	13	16	19	43	0.2571	0.1305	0.1473
WRC002	Bradi	445	11	14	16	48	0.1333	0.1478	0.1405
WRC003	Bradi	446	11	12	17	48	0.8621	0.1341	0.1471
WRC004	Bradi	444	11	14	17	48	0.2903	0.1478	0.1470
WRC005	Bradi	445	11	14	16	48	0.1333	0.1478	0.1405
WRC006	Bradi	445	11	14	16	48	0.1333	0.1478	0.1405
WRC007	Bradi	444	11	14	17	48	0.2903	0.1478	0.1471
WRC009	Bradi	443	13	16	20	42	0.4444	0.1288	0.1473
WRC010	Bradi	443	13	16	19	43	0.2571	0.1305	0.1473
WRC011	Bradi	445	11	14	16	48	0.1333	0.1478	0.1405
WRC013	Bradi	444	11	14	17	48	0.2903	0.1478	0.1471
WRC014	Bradi	443	13	16	19	43	0.2571	0.1305	0.1473
WRC015	Bradi	445	11	14	16	48	0.1333	0.1478	0.1405
WRC016	Bradi	444	11	14	17	48	0.2903	0.1478	0.1471
WRC017	Bradi	444	11	14	17	48	0.2903	0.1478	0.1471
WRC019	Bradi	445	11	14	16	48	0.1333	0.1478	0.1405
WRC020	Bradi	444	11	14	17	48	0.2903	0.1478	0.1471
WRC021	Bradi	442	13	17	19	43	0.1111	0.1270	0.1473
WRC022	Bradi	443	13	16	19	43	0.2571	0.1305	0.1473
WRC023	Bradi	443	13	16	19	43	0.2571	0.1305	0.1473
WRC024	Bradi	443	13	16	19	43	0.2571	0.1305	0.1473
WRC025	Bradi	445	11	14	16	48	0.1333	0.1478	0.1405
WRC026	Bradi	445	11	14	16	48	0.1333	0.1478	0.1405
WRC027	Bradi	444	11	14	17	48	0.2903	0.1478	0.1470
WRC028	Bradi	444	11	14	17	48	0.2903	0.1478	0.1470
WRC029	Bradi	445	11	14	16	48	0.1333	0.1478	0.1405
WRC030	Bradi	444	11	14	17	48	0.2903	0.1478	0.1470
WRC031	Bradi	445	11	14	16	48	0.1333	0.1478	0.1405
WRC033	Bradi	445	11	14	16	48	0.1333	0.1478	0.1405
WRC034	Bradi	445	11	14	16	48	0.1333	0.1478	0.1405
WRC035	Bradi	445	11	14	16	48	0.1333	0.1478	0.1405
WRC036	Bradi	444	11	14	17	48	0.2903	0.1478	0.1470
WRC037	Bradi	445	11	14	16	48	0.1333	0.1478	0.1405
WRC038	Bradi	445	11	14	16	48	0.1333	0.1478	0.1405
WRC039	Bradi	444	11	14	17	48	0.2903	0.1478	0.1470
WRC040	Bradi	444	11	14	17	48	0.2903	0.1478	0.1470
WRC042	Bradi	443	13	16	19	43	0.2571	0.1305	0.1473
WRC043	Bradi	443	13	16	19	43	0.2571	0.1305	0.1473
WRC044	Bradi	443	13	16	19	43	0.2571	0.1305	0.1473
WRC046	Bradi	442	13	16	20	43	0.4444	0.1305	0.1540
WRC047	Bradi	443	13	16	19	43	0.2571	0.1305	0.1473
WRC048	Bradi	442	13	16	20	43	0.4444	0.1305	0.1540
WRC049	Bradi	443	13	15	19	44	0.4706	0.1305	0.1540
WRC050	Bradi	445	11	14	16	48	0.1333	0.1478	0.1405
WRC051	Bradi	443	13	16	19	43	0.2571	0.1305	0.1473
WRC053	Bradi	442	13	17	19	43	0.1111	0.1373	0.1473
WRC055	Bradi	442	13	17	19	43	0.1111	0.1373	0.1473
WRC057	Bradi	442	13	17	19	43	0.1111	0.1270	0.1473
WRC058	Bradi	446	11	12	17	48	0.8621	0.1341	0.1471
WRC059	Bradi	444	11	14	17	48	0.2903	0.1478	0.1471
WRC060	Bradi	444	11	14	17	48	0.2903	0.1478	0.1471
WRC061	Bradi	445	11	14	16	48	0.1333	0.1478	0.1405
WRC062	Bradi	444	11	14	17	48	0.2903	0.1478	0.1471
WRC064	Bradi	445	11	13	17	48	0.5333	0.1305	0.1471
WRC065	Bradi	444	11	14	17	48	0.2903	0.1478	0.1471
WRC066	Bradi	445	11	14	16	48	0.1333	0.1478	0.1405
WRC067	Bradi	443	13	16	19	43	0.2571	0.1305	0.1473
WRC068	Bradi	443	13	16	19	43	0.2571	0.1305	0.1473
WRC096	Bradi	444	11	14	17	48	0.2903	0.1478	0.1471
WRC097	Bradi	442	13	16	20	43	0.4444	0.1305	0.1540
WRC098	Bradi	444	11	14	17	48	0.2903	0.1478	0.1471
WRC099	Bradi	444	11	14	17	48	0.2903	0.1478	0.1471
WRC100	Bradi	444	11	14	17	48	0.2903	0.1478	0.1471
