# Supplementary material for: Natural Variation of the RICE FLOWERING LOCUS T 1 Contributes to Flowering Time Divergence in Rice
Source: PLoS One. 2013 Oct 1;8(10):e75959. doi: 10.1371/journal.pone.0075959 (PMC3788028; doi:10.1371/journal.pone.0075959)
Supplement: Table S5 — Tajima’s relative rate test for Hd3a and RFT1 using SbHd3a ( Sorghum bicolor ) DNA sequence as an out group. (PDF) [file pone.0075959.s016.pdf]

Table S5: Tajima's Relative Rate Test for *Hd3a* and *RFT1* with *SbHd3a* (*Shorgum bicolor*) DNA sequences

Accession	Outgroup	Identical sites	Unique sites	Unique Sites			$\chi 2$	dN/dS	
				Hd3a	*RFT1*	Outgroup		*Hd3a*	*RFT1*
101508	Sb	437	11	26	17	43	1.8837	0.1484	0.1973
105908	Sb	437	10	22	14	51	1.7778	0.1570	0.1927
A001	Sb	440	10	19	13	52	1.1250	0.1497	0.1927
A002	Sb	438	10	23	15	48	1.6842	0.1376	0.1985
A006	Sb	440	10	19	13	52	1.1250	0.1497	0.1927
A008	Sb	440	10	19	13	52	1.1250	0.1497	0.1926
A011	Sb	440	10	19	13	52	1.1250	0.1497	0.1927
A014	Sb	441	10	19	12	52	1.5806	0.1497	0.1850
A015	Sb	440	10	19	13	52	1.1250	0.1497	0.1927
A016	Sb	440	10	19	13	52	1.1250	0.1497	0.1927
A018	Sb	438	10	23	15	48	1.6842	0.1376	0.1985
A019	Sb	438	10	23	15	48	1.6842	0.1376	0.1985
A020	Sb	440	10	19	13	52	1.1250	0.1497	0.1926
A021	Sb	440	10	19	13	52	1.1250	0.1497	0.1927
A022	Sb	440	10	19	13	52	1.1250	0.1497	0.1927
A024	Sb	440	10	19	13	52	1.1250	0.1497	0.1927
A025	Sb	437	10	23	16	48	1.2564	0.1376	0.2065
A027	Sb	437	10	23	16	48	1.2564	0.1376	0.2065
A028	Sb	441	9	18	13	53	0.8065	0.1412	0.1927
A030	Sb	441	9	18	13	53	0.8065	0.1412	0.1927
A032	Sb	440	10	19	13	52	1.1250	0.1497	0.1926
A035	Sb	440	10	19	13	52	1.1250	0.1497	0.1927
A036	Sb	440	10	19	13	52	1.1250	0.1497	0.1927
A037	Sb	440	10	19	13	52	1.1250	0.1497	0.1927
A039	Sb	440	10	19	13	52	1.1250	0.1497	0.1926
A040	Sb	441	10	19	12	52	1.5806	0.1497	0.1850
A041	Sb	437	10	24	15	48	2.0769	0.1437	0.1985
A045	Sb	438	10	23	15	48	1.6842	0.1376	0.1985
A047	Sb	437	10	23	16	48	1.2564	0.1376	0.2065
A048	Sb	440	10	19	13	52	1.1250	0.1497	0.1927
A049	Sb	440	10	19	13	52	1.1250	0.1497	0.1927
A050	Sb	438	10	23	15	48	1.6842	0.1376	0.1985
A053	Sb	438	10	23	15	48	1.6842	0.1376	0.1985
A054	Sb	441	10	19	12	52	1.5806	0.1497	0.1850
A055	Sb	437	10	23	16	48	1.2564	0.1376	0.2065
A056	Sb	440	10	19	13	52	1.1250	0.1497	0.1927
A059	Sb	440	10	19	13	52	1.1250	0.1497	0.1927
A063	Sb	441	9	18	13	53	0.8065	0.1412	0.1926
A064	Sb	440	10	19	13	52	1.1250	0.1497	0.1927
A074	Sb	438	10	23	15	48	1.6842	0.1376	0.1985
A082	Sb	440	10	19	13	52	1.1250	0.1497	0.1926
A086	Sb	437	11	26	17	43	1.8837	0.1484	0.1973
A087	Sb	437	11	26	17	43	1.8837	0.1484	0.1973
A091	Sb	442	10	17	13	52	0.5333	0.1473	0.1927
A092	Sb	437	10	22	14	51	1.7778	0.1568	0.1927
A096	Sb	439	10	21	13	51	1.8824	0.1612	0.1850
A100	Sb	436	11	25	17	45	1.5238	0.1548	0.1985
A103	Sb	438	10	22	13	51	2.3143	0.1674	0.1850
A107	Sb	437	11	26	17	43	1.8837	0.1484	0.1973
A112	Sb	441	10	18	13	52	0.8065	0.1537	0.1926
A114	Sb	437	12	23	15	47	1.6842	0.1459	0.1985
AS017	Sb	440	10	19	13	52	1.1250	0.1497	0.1926
AS062	Sb	440	10	19	13	52	1.1250	0.1497	0.1926
AS083	Sb	439	10	21	13	51	1.8824	0.1506	0.1724
AS084	Sb	437	9	22	13	53	2.3143	0.1631	0.1957
AS088	Sb	435	11	26	16	46	2.3810	0.1568	0.1985
AS089	Sb	441	10	18	13	52	0.8065	0.1537	0.1926
AS092	Sb	436	10	24	13	51	3.2703	0.1589	0.1850

JRC012	Sb	438	10	23	15	48	1.6842	0.1376	0.1985
JRC021	Sb	440	10	19	13	52	1.1250	0.1497	0.1927
JRC022	Sb	438	10	23	15	48	1.6842	0.1376	0.1985
JRC027	Sb	438	10	23	15	48	1.6842	0.1376	0.1985
JRC033	Sb	437	10	24	15	48	2.0769	0.1341	0.1985
JRC034	Sb	438	10	23	15	48	1.6842	0.1376	0.1985
JRC042	Sb	440	10	19	13	52	1.1250	0.1497	0.1927
JRC047	Sb	438	10	23	15	48	1.6842	0.1376	0.1985
NonaBokra	Sb	440	10	19	13	52	1.1250	0.1497	0.1927
O_brachyantha	Sb	439	8	25	18	44	1.1395	0.1775	0.2370
O_longistamina	Sb	438	11	27	15	43	3.4286	0.1538	0.1948
O_meridionalis	Sb	435	9	22	19	49	0.2195	0.1484	0.1833
O_punctata	Sb	435	8	21	22	48	0.0233	0.1608	0.1489
W0106	Sb	439	10	20	13	52	1.4848	0.1554	0.1927
W0157	Sb	434	11	27	16	46	2.8140	0.1631	0.1985
W1294	Sb	433	11	25	19	46	0.8182	0.1409	0.2448
W1666	Sb	436	10	23	14	51	2.1892	0.1530	0.1927
W1807	Sb	439	10	20	14	51	1.0588	0.1446	0.1926
W1865	Sb	439	10	20	14	51	1.0588	0.1446	0.1926
W1943	Sb	436	11	25	17	45	1.5238	0.1548	0.1985
W1944	Sb	436	11	25	17	45	1.5238	0.1548	0.1985
W2014	Sb	436	11	25	17	45	1.5238	0.1548	0.1985
W2265	Sb	438	10	22	13	51	2.3143	0.1570	0.1850
WRC001	Sb	438	10	23	15	48	1.6842	0.1376	0.1985
WRC002	Sb	441	10	19	12	52	1.5806	0.1497	0.1850
WRC003	Sb	441	9	18	13	53	0.8065	0.1412	0.1927
WRC004	Sb	440	10	19	13	52	1.1250	0.1497	0.1926
WRC005	Sb	441	10	19	12	52	1.5806	0.1497	0.1850
WRC006	Sb	441	10	19	12	52	1.5806	0.1497	0.1850
WRC007	Sb	440	10	19	13	52	1.1250	0.1497	0.1927
WRC009	Sb	438	10	23	16	47	1.2564	0.1362	0.1985
WRC010	Sb	438	10	23	15	48	1.6842	0.1376	0.1985
WRC011	Sb	441	10	19	12	52	1.5806	0.1497	0.1850
WRC013	Sb	440	10	19	13	52	1.1250	0.1497	0.1927
WRC014	Sb	438	10	23	15	48	1.6842	0.1376	0.1985
WRC015	Sb	441	10	19	12	52	1.5806	0.1497	0.1850
WRC016	Sb	440	10	19	13	52	1.1250	0.1497	0.1927
WRC017	Sb	440	10	19	13	52	1.1250	0.1497	0.1927
WRC019	Sb	441	10	19	12	52	1.5806	0.1497	0.1850
WRC020	Sb	440	10	19	13	52	1.1250	0.1497	0.1927
WRC021	Sb	437	10	24	15	48	2.0769	0.1341	0.1985
WRC022	Sb	438	10	23	15	48	1.6842	0.1376	0.1985
WRC023	Sb	438	10	23	15	48	1.6842	0.1376	0.1985
WRC024	Sb	438	10	23	15	48	1.6842	0.1376	0.1985
WRC025	Sb	441	10	19	12	52	1.5806	0.1497	0.1850
WRC026	Sb	441	10	19	12	52	1.5806	0.1497	0.1850
WRC027	Sb	440	10	19	13	52	1.1250	0.1497	0.1926
WRC028	Sb	440	10	19	13	52	1.1250	0.1497	0.1926
WRC029	Sb	441	10	19	12	52	1.5806	0.1497	0.1850
WRC030	Sb	440	10	19	13	52	1.1250	0.1497	0.1926
WRC031	Sb	441	10	19	12	52	1.5806	0.1497	0.1850
WRC033	Sb	441	10	19	12	52	1.5806	0.1497	0.1850
WRC034	Sb	441	10	19	12	52	1.5806	0.1497	0.1850
WRC035	Sb	441	10	19	12	52	1.5806	0.1497	0.1850
WRC036	Sb	440	10	19	13	52	1.1250	0.1497	0.1926
WRC037	Sb	441	10	19	12	52	1.5806	0.1497	0.1850
WRC038	Sb	441	10	19	12	52	1.5806	0.1497	0.1850
WRC039	Sb	440	10	19	13	52	1.1250	0.1497	0.1926
WRC040	Sb	440	10	19	13	52	1.1250	0.1497	0.1926
WRC042	Sb	438	10	23	15	48	1.6842	0.1376	0.1985
WRC043	Sb	438	10	23	15	48	1.6842	0.1376	0.1985

WRC044	Sb	438	10	23	15	48	1.6842	0.1376	0.1985
WRC046	Sb	437	10	23	16	48	1.2564	0.1376	0.2065
WRC047	Sb	438	10	23	15	48	1.6842	0.1376	0.1985
WRC048	Sb	437	10	23	16	48	1.2564	0.1376	0.2065
WRC049	Sb	438	10	22	15	49	1.3243	0.1376	0.2065
WRC050	Sb	441	10	19	12	52	1.5806	0.1497	0.1850
WRC051	Sb	438	10	23	15	48	1.6842	0.1376	0.1985
WRC053	Sb	437	10	24	15	48	2.0769	0.1437	0.1985
WRC055	Sb	437	10	24	15	48	2.0769	0.1437	0.1985
WRC057	Sb	437	10	24	15	48	2.0769	0.1341	0.1985
WRC058	Sb	441	9	18	13	53	0.8065	0.1412	0.1927
WRC059	Sb	440	10	19	13	52	1.1250	0.1497	0.1927
WRC060	Sb	440	10	19	13	52	1.1250	0.1497	0.1927
WRC061	Sb	441	10	19	12	52	1.5806	0.1497	0.1850
WRC062	Sb	440	10	19	13	52	1.1250	0.1497	0.1927
WRC064	Sb	440	9	19	13	53	1.1250	0.1376	0.1927
WRC065	Sb	440	10	19	13	52	1.1250	0.1497	0.1927
WRC066	Sb	441	10	19	12	52	1.5806	0.1497	0.1850
WRC067	Sb	438	10	23	15	48	1.6842	0.1376	0.1985
WRC068	Sb	438	10	23	15	48	1.6842	0.1376	0.1985
WRC096	Sb	440	10	19	13	52	1.1250	0.1497	0.1927
WRC097	Sb	437	10	23	16	48	1.2564	0.1376	0.2065
WRC098	Sb	440	10	19	13	52	1.1250	0.1497	0.1927
WRC099	Sb	440	10	19	13	52	1.1250	0.1497	0.1927
WRC100	Sb	440	10	19	13	52	1.1250	0.1497	0.1927
