# Supplementary material for: Natural Variation of the RICE FLOWERING LOCUS T 1 Contributes to Flowering Time Divergence in Rice
Source: PLoS One. 2013 Oct 1;8(10):e75959. doi: 10.1371/journal.pone.0075959 (PMC3788028; doi:10.1371/journal.pone.0075959)
Supplement: Table S6 — SNP information for Haploview plot. (PDF) [file pone.0075959.s017.pdf]

Table S6 : SNP information

SNP name	Chromosome	Positions	RefBase	SNPBase	RefNumber	SNPNumber
PeakSNP	6	2912415	A	G	531	419
interval	6	2922659	T	A	512	433
RFT1pro_1	6	2924005	T	A	514	436
RFT1pro_2	6	2924583	G	A	822	112
RFT1pro_3	6	2924782	A	G	829	114
RFT1pro_4	6	2924932	G	A	648	294
RFT1pro_5	6	2924990	G	C	645	298
RFT1pro_6	6	2925031	G	A	834	110
RFT1pro_7	6	2925070	C	T	531	420
RFT1pro_8	6	2925147	T	C	531	414
RFT1pro_9	6	2925171	G	A	573	300
RFT1pro_10	6	2925202	G	A	532	413
RFT1pro_11	6	2925233	G	C	532	416
RFT1pro_12	6	2925353	T	A	642	300
RFT1pro_13	6	2925508	G	A	837	110
RFT1pro_14	6	2925518	C	T	645	294
RFT1pro_15	6	2925543	G	A	835	112
RFT1pro_16	6	2925695	A	G	519	402
RFT_exon1	6	2925914	G	T	653	10
RFT_exon1_2	6	2926161	T	C	529	415
RFT1_V>A	6	2926172	T	C	530	415
RFT1intron1	6	2926389	T	A	661	234
RFT1_P>S	6	2926623	C	T	935	10
RFT1intron3_1	6	2926689	C	A	926	8
RFT1intron3_2	6	2927035	A	G	829	63
RFT1intron3_3	6	2927060	T	C	366	16
RFT1_E105K	6	2927179	G	A	695	169
Hd3apro_1	6	2937180	G	C	369	15
Hd3apro_2	6	2937333	A	G	511	425
Hd3apro_3	6	2938175	C	A	927	14
Hd3apro_4	6	2938295	C	T	834	114
Hd3apro_5	6	2938469	C	T	508	436
Hd3apro_6	6	2938488	C	A	636	305
Hd3apro_7	6	2938653	T	C	622	237
Hd3apro_8	6	2938739	A	G	511	434
Hd3apro_9	6	2938839	C	A	532	411
Hd3a_exon1_1	6	2939078	C	A	644	302
Hd3a_exon1_2	6	2939131	A	T	509	436
Hd3a_intron1_1	6	2939396	G	T	833	115
Hd3a_intron1_2	6	2939505	T	G	527	422
Hd3a_intron3_1	6	2940098	A	T	511	422
Hd3a_intron3_2	6	2940201	T	C	507	440
Hd3a_intron3_3	6	2940270	T	C	833	114
Hd3a_intron3_4	6	2940347	T	G	834	114
Hd3a_intron3_5	6	2940864	C	A	640	288
Hd3a_intron3_6	6	2941053	C	T	508	441
Hd3a_P179N	6	2941150	A	C	147	70
Hd3a_3UTR	6	2941433	A	T	511	424

*RefBase: refererence genome base (IRGSP Build 4.0)
