# Supplementary material for: Natural Variation of the RICE FLOWERING LOCUS T 1 Contributes to Flowering Time Divergence in Rice
Source: PLoS One. 2013 Oct 1;8(10):e75959. doi: 10.1371/journal.pone.0075959 (PMC3788028; doi:10.1371/journal.pone.0075959)
Supplement: Table S7 — Primer sequences of newly designed DNA markers used for linkage mapping. Amplified fragment size differences are shown in the “Name” column. Target SNPs and restriction enzymes used to detect polymorphism are shown for CAPS markers. SNP-1, 2 and 3 are SNP marker. These target SNPs are shown as red character in Note/SNP column. These SNPs were detected by using Acyclo-Prime FP detection kit (PerkinElmer Life Science). Primers for expression analysis were previously described [14,19]. (PDF) [file pone.0075959.s018.pdf]

Table S7

Primer sequences for genotyping

Name	Forward	Reverse	Note / SNP (Kos/Non)
InDel-1	GTGTTGCAGCTTGAGGATGA	TGTATTGGCCGTAATCGTGA	Electrophoresis
InDel-2	TACTGCAGCTGCTGATTGCT	GCGGGATACCATGGTCTAAA	Electrophoresis
SNP-1	AACTTCCGGACAACACTTGG	CAAGCAATCCAAGCAACTGA	TAAAATCATTAGTCCCTTAAC<br>TAAAATCATTTGTCCCTTAAC
InDel-3	CTCTTGTGGTTGGCAGGAT	GGTCTACCATCACCTGTAGG	Electrophoresis
InDel-4	CCTGTCACTGTTTGGCTAG	GTCAAATTAATAACCTCTAACTAA	sequencing
InDel-5	TTATTACCTTGGTCCTACCCC	CTAGGGGTAGACCCTCCTG	sequencing
CAPs-1	ATTTGAAGGATAGGGCTGTAC	ATACAGCTAGGCAGGTCTCA	SmaI digestion
CAPs-2	TACGGGCTACGGACATAACA	CGCTCAGCAACGAGTTTC	TaqI digestion
SNP-3	CAGGTCCTGGCATTCAAAAA	AATGAAACGCCGACGATATT	TGCATAAAAGCGTGTTGTTTA<br>TACATAAAAGTGTGTTGTTTA
SNP-2	CTCGTCCACACGTACAGGAA	AGGTGACCTTGAGGTTGGTG	TTGCTCCCTGCAACTTGCTGC<br>TTGCTCCCTGAAACTTGCTGC
CAPs-3	TCA GAACTTCAACACCAAGG	ACCTTAGCCTTGCTCAGCTA	HaeIII digestion

Primer sequences for *RFT1* and *Hd3a* sequencing

Name	Forward	Reverse
RFT1_5_3	CCATCCCTGTCCAATAATGC	TTTTACAAGCTTCATCTCTC
RFT1_5_1	CCACCACACCTTTCAAGACA	ACCATGTCTCATGCAAGACC
RFT1_5_2	CTGGGCACATCATAGTAAGG	AGGTTAGTGATCCGGACGAA
RFT1_exon1	TGGCTAGCTTAACCTTCCTG	GTCTACCATCACCTGTAGGT
RFT1_exon2	TATCTTGGATGCAGAGACCC	ATGGGGTAGGACCCAAGGTAA
RFT1_intron_1	TCAATTTCGATGTAACAGCA	TTATCTACGTTATTCACCAT
RFT1_exon4	CGGAGGGAGTATCTATTTTG	CACACTTAAGAGCCTGCATG
Hd3a_5_4	CACAGCTAGAGATGGTATCG	ATGAAATCATAATTAAAAGG
Hd3a_5_3	CTTAAATTTTGGATGTGTGT	CCGCAACTTATCAACCCGTA
Hd3a_5_1	CGGAAATGGTATAAAACGGG	TCTGCATCGTCTTCCTGTAC
Hd3a_5_2	GCTCGATCATATCCCATCTC	TAGGTGACCTTGAGGTTGGT
Hd3a_exon1	CAGCTGCAGCTAGTAAGCAA	TCTGCACCAACTACGACACA
Hd3a_exon2	TGTGTCGTAGTTGGTGCAGA	CCCCCAAACAATAGTATGGA
Hd3a_exon4	AGCTGTTGCACATGCTCATG	CTGAACCTGCAATGTATAGC

Primer sequences for *RFT1(E105K)* construction

Name	
RFT1-XhoI-F	CTCGAGCCTGTCACTGTTTGGCTAG
RFT1-E105K-R	CACCTTTTGCCCAAATGTTGCTCCAGTG
RFT1-E105K-F	TTGGGCAAAAGGTGATGTGCTACGAGAG
RFT1-NotI-R	CGCGGCCGCCTCGAGCCAGGGGTAGACCCTCCTG
