# Supplementary material for: Natural Variation of the RICE FLOWERING LOCUS T 1 Contributes to Flowering Time Divergence in Rice
Source: PLoS One. 2013 Oct 1;8(10):e75959. doi: 10.1371/journal.pone.0075959 (PMC3788028; doi:10.1371/journal.pone.0075959)
Supplement: File S1 — Supplementary References. (DOCX) [file pone.0075959.s019.docx]

# Supplementary References

1. Network 4.611. Available: <http://www.fluxus-engineering.com/sharenet.htm> Accessed 2013 Aug. 29.

2. R(qtl). Available: <http://www.rqtl.org/> Accessed 2013 Aug. 29.

3. Barrett JC, Fry B, Maller J, Daly MJ (2005) Haploview: analysis and visualization of LD and haplotype maps. Bioinformatics 21: 263–265.
